# Use of circulating tumour DNA to prospectively guide a switch from targeted to immune therapy in *BRAF* mutant advanced melanoma: the randomised phase II CAcTUS trial

**DOI:** 10.1038/s41467-026-72735-8

**Published:** 2026-05-21

**Authors:** Rebecca J. Lee, Dominic G. Rothwell, Nigel Smith, Shien Chow, Juan Delgado-SanMartin, Hitesh Mistry, Yvonne Sylvestre, Shih-Chieh Chiang, Harry Clarke, Gabriela Gremel, Avinash Gupta, Kimberley Hockenhull, Noel Kelso, Rohit Kochhar, Damian Mullan, Ruth Plummer, Patricio Serra, Heather Shaw, Holly Summersgill, Samra Turajlic, Florent Mouliere, Richard Marais, Caroline Dive, Paul Lorigan

**Affiliations:** 1 Division of Cancer Sciences, School of Medical Sciences, Faculty of Biology, Medicine and Health, University of Manchester, Manchester, UK; 2 The Christie NHS Foundation Trust, Manchester, UK; 3 Cancer Research UK National Biomarker Centre, University of Manchester, Manchester, UK; 4 Department of Medical Oncology, Clatterbridge Cancer Centre, Liverpool, UK; 5 NHLI Data Science, Imperial College London, London, UK; 6 Systems Forecasting, Manchester, UK; 7 Division of Population Health, Health Services Research & Primary Care, Faculty of Biology, Medicine and Health, University of Manchester, Manchester, UK; 8 Cancer Inflammation and Immunity Group, Cancer Research UK Manchester Institute, The University of Manchester, Manchester, UK; 9 Molecular Oncology Group, Cancer Research UK Manchester Institute, Manchester, UK; 10 Sir Bobby Robson Cancer Trials Research Centre, Northern Centre for Cancer Care, Freeman Hospital, Newcastle upon Tyne, UK; 11 Newcastle University, Newcastle upon Tyne, UK; 12 Department of Oncology, University College London Hospitals NHS Foundation Trust, London, UK; 13 Skin and Renal Units, The Royal Marsden NHS Foundation Trust, London, UK; 14 Cancer Dynamics Laboratory, The Francis Crick Institute, London, UK; 15 Oncodrug Ltd, Macclesfield, UK

**Keywords:** Melanoma, Cancer therapeutic resistance, DNA sequencing

## Abstract

Checkpoint inhibitor immunotherapy (CPI) for *BRAF* mutant advanced melanoma first-line results in a better long-term survival compared to targeted therapy (TT), however TT induction may benefit poor prognosis groups. The parallel-arm, randomised phase II, multicentre, feasibility CAcTUS trial (Clinicaltrials.gov NCT03808441) randomised 21 patients to receive standard of care investigators choice TT or CPI, switching to the alternative upon progression (*n* = 10), or commencing TT and switching to CPI upon an ≥80% reduction of *BRAF* variant allele frequency *(*VAF) in circulating tumour DNA (ctDNA; n = 11). The study achieved its primary endpoints with 100% (95% confidence interval [CI]: 94-100%) of critical results provided within 7 days to inform a decision to switch and 100% of patients commencing TT achieving an ≥80% reduction of *BRAF* VAF (95% CI: 80-100%). Secondary outcomes included progression-free survival and overall survival. No new safety signals were observed for TT/CPI. Post-hoc analysis of clinical features, circulating cytokines and chemokines at ctDNA nadir following TT induction suggested a more favourable profile prior to CPI initiation. Longitudinal ctDNA dynamics revealed ctDNA provided an early signal of CPI benefit and that rechallenge with TT following CPI progression resulted in a further ctDNA response. These data support the utility of ctDNA to guide treatment decision-making within a clinically relevant timeframe to optimise treatment scheduling strategies.

## Abstract (web summary)

Targeted therapy (with BRAF and MEK inhibitors) and immune checkpoint blockade have improved survival for patients with advanced BRAF-mutant cutaneous melanoma, however, the optimal scheduling for these treatments remain to be further refined in poor prognosis groups. Here the authors present the results of a feasibility trial to determine the role of circulating tumour DNA in guiding a switch between targeted therapy and immune therapy in patients with advanced melanoma.

## Introduction

The advent of targeted therapy (TT; BRAF + MEK inhibitors) and CPI (anti-PD-1 ± anti-CTLA-4) have significantly improved overall survival (OS) for patients with advanced *BRAF* mutant cutaneous melanoma^1–3^. More recently, several trials and pre-clinical studies have examined the optimal scheduling of these treatments. The DREAMSeq trial showed that CPI with standard dose ipilimumab plus nivolumab (N + I) first line and TT with dabrafenib plus trametinib as second line in patients progressing on CPI, was associated with a 20% absolute improvement in 2-year OS (72% (95% CI: 62.5 to 79.1) vs. 52% (95% CI: 41.7 to 60.4), *p *= 0.010) compared to the reverse schedule^4^. Studies have shown that when tumours are responding to TT, the tumour microenvironment can have increased T-cell infiltration, improved T-cell recognition of melanoma-associated antigens and reduced production of immunosuppressive cytokines^5–7^. Mechanistic pre-clinical data have shown that in melanoma with acquired resistance to TT, a decrease in CD103+ dendritic cells is observed, resulting in inferior antigen presentation and response to subsequent CPI^8^. Thus, there appears to be an ‘optimum point’ where patients responding to TT are also most likely to derive benefit from switching to CPI, before the tumour becomes resistant to TT and thus less likely to respond to immunotherapy. Based on this concept, the SECOMBIT study (NCT02631447) examining optimal treatment scheduling of TT and CPI in advanced melanoma, included an experimental arm where N + I was preceded by an 8-week run-in of TT with encorafenib plus binimetinib (E + B), with patients reverting to E + B if progression occurred on N + I^9^. SECOMBIT showed that 3-year OS for N + I first and the experimental arm were comparable, whilst patients starting on TT and switching to CPI on progression had a poorer outcome^9^. The EBIN study (NCT03235245) evaluated a fixed dose run-in with 12 weeks of targeted therapy E + B and elective switch to immunotherapy, compared to upfront immunotherapy with N + I^10^. There was no difference in progression-free survival (PFS) between N + I first and the induction TT then N + I (HR: 0.87, 95% CI: 0.67–1.12, *p* = 0.36) for the whole trial population^10^. However, in a pre-specified sub-group analysis of patients with adverse prognostic factors, there was a benefit for the induction in patients with LDH >2xULN (HR: 0.46; 95% CI: 0.21–1.03) and in those with liver metastasis (HR: 0.48; 95% CI: 0.28–0.80, *p* value for interaction 0.008)^10^. Both studies used a pre-defined TT induction period in the experimental arm, though the optimal length of the induction period, if any, remains unclear.

We and others have shown that circulating tumour DNA (ctDNA) can be used to accurately follow tumour burden over time and that ctDNA monitoring is acceptable to patients^11–16^. Critically, by analysing tumour-specific mutations in the blood, we were able to identify treatment response and disease progression at an early stage^13,15^. We therefore hypothesised that ctDNA could be used to tailor the duration of TT and identify the optimum time to switch to CPI, when the tumour was responding to TT, and therefore most likely to respond to CPI therapy. Informed by pre-clinical data (Supplementary Fig. 1a) and the variance criteria for the test, we determined that even those patients with high disease burden were likely to have an ≥80% reduction of *BRAF* variant allele frequency (VAF) early on TT, although the response might be short-lived. Not all patients completely cleared their ctDNA at maximal response. Defining complete ctDNA clearance as the time point to switch would prevent some patients from switching to immunotherapy early in the course of their treatment, risking the emergence of targeted therapy-resistant clones, which would be less likely to respond to CPI therapy. We therefore designed the CAcTUS trial to test whether personalising a treatment switch from TT to CPI guided by an ≥80% reduction of *BRAF* VAF would enable us to better define that ‘optimum point’ between response and resistance to TT and so individualise the decision to change treatment electively.

## Results

### Real-time clinical decision-making based on circulating tumour DNA level is deliverable

To enable individualised monitoring of response to treatment we developed and validated (to good clinical practice regulations, see ‘Validation of droplet digital PCR (ddPCR) BRAF V600 assay for the CAcTUS trial’ report Supplementary Information) droplet digital assays (ddPCR) for the detection of *BRAF* V600E, K and R mutations. We used these assays to detect an 80% *BRAF* VAF drop in ctDNA when blood was longitudinally sampled. Assay specificity was demonstrated previously for all ddPCR *BRAF* assays using healthy volunteer circulating free DNA (cfDNA) (HNV), where all samples were found to be negative^11^. We confirmed assay specificity using cell line DNA with known BRAF status, where a VAF of 0% was observed in all negative controls (*n* = 3 HNV in triplicate, *n* = 2 experiments, Supplementary Data, negative in all cell lines without the mutation present, Supplementary Table 1). Assay reproducibility (%VAF difference between wells of the same experiment) and repeatability (between 3 different experiments) was demonstrated to be within ±5% of the determined %VAF (Supplementary Tables 2 and 3) for all assays. To demonstrate the VAF lower limit of detection (LLOD) and lower limit of quantitation (LLOQ), mutant positive cell-line DNA was mixed with wild-type cell-line DNA in serial dilution and a standard curve produced (Supplementary Table 3, Supplementary Fig. 1b, c). All wildtype controls had 0.0% VAF. Using this, we determined that the lowest input for the assay to detect ≥3 mutant positive droplets (LLOD) was 0.40 ng and 2.12 ng to detect ≥10 mutant positive droplets (LLOQ), and therefore we planned to have a minimum of 5 ng input cfDNA to reliably determine an 80% *BRAF* VAF drop with treatment.

**Fig. 1:**
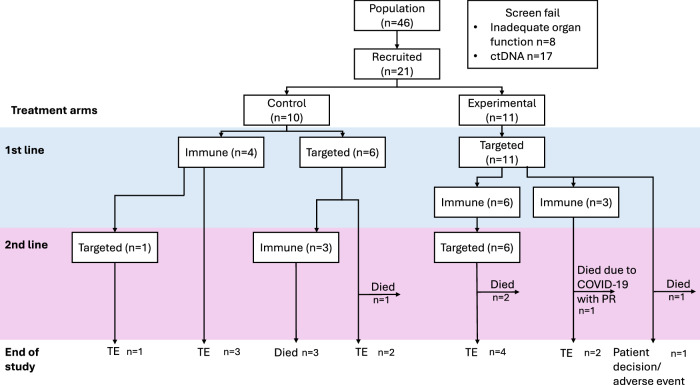
Consort diagram. End of study reasons: patient withdrawal (*n* = 1), died (*n* = 8), TE; Alive at end of trial follow-up (*n* = 12). Immune: nivolumab plus ipilimumab. Targeted: dabrafenib plus trametinib.

Having demonstrated the reliability of the assay, between 2nd May 2019 and 30th May 2022, we randomised 21 patients to the trial (Consort diagram, Fig. 1). The control arm (*n* = 10) consisted of either investigators’ choice first-line immunotherapy with nivolumab 1 mg/kg and ipilimumab 3 mg/kg given 3-weekly for 4 cycles then nivolumab maintenance 3 mg/kg 4-weekly, (N + I) followed on progression by second-line TT (dabrafenib 150 mg bd + trametinib 2 mg od), (D + T), or the reverse schedule (Supplementary Fig. 1d). In the experimental arm of ctDNA guided therapy (*n* = 11), patients were commenced on D + T, had 2 weekly ctDNA assessments for the first 4 weeks then 4-weekly based on standard of care visit schedules and switched to N + I when an ≥80% decrease in *BRAF* VAF from the baseline pre-treatment sample was observed. If the ≥80% decrease in *BRAF* VAF was not achieved, patients would continue on TT. To reduce the incidence of ‘crossover’ toxicity when switching treatments, we stipulated a wash-out period based on drug half-lives. Trametinib was stopped 7 days prior to first cycle of N + I, and dabrafenib stopped the day before. If patients subsequently progressed on CPI, they could then recommence D + T, under the premise that resistance to D + T had not developed, as they were initially switched when still responding (Supplementary Fig. 1d).

**Fig. 2:**
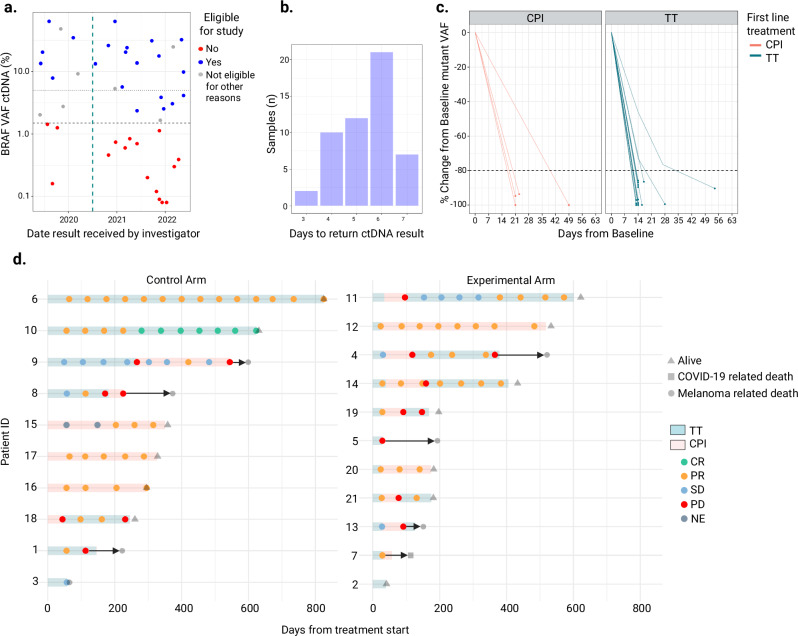
Real-time clinical decision-making based on circulating tumour DNA is deliverable. **a** Comparison of different screening inclusion criteria ctDNA VAF cut-offs. Distribution of screening ctDNA samples (average of 2 separate ctDNA tests) over time and eligibility of patients (*n* = 44, *n* = 16 not eligible, *n* = 21 eligible and *n* = 7 not eligible for other reasons) to enter the study pre and post protocol amendment, depending on inclusion criteria cut-off of 5% and 1.5% VAF. Dotted line relates to the original cut-off of 5%, and the dashed line the amended cut-off value of 1.5%, green dotted line represents the date of protocol amendment. **b** Distribution of days to return sample reports for critical samples for *n* = 44 patients. Time to report critical samples defined as time required for a clinical decision to be made. **c** Initial percentage change from baseline in ctDNA variant allele frequency (VAF) for each patient (*n* = 21), depending on initial treatment commenced (targeted therapy [TT, teal] *n* = 17 or immune therapy [CPI, salmon] *n* = 4), with 80% threshold shown (dashed line). TT visits were 2 weekly, and CPI were 3 weekly according to treatment schedules. Source data are provided as a Source data file. **d** Swimmer plot for *n* = 21 patients (10 control, 11 experimental arm), showing treatment administered (immune therapy [CPI, pink] vs. targeted therapy [TT, blue]), response type (CR complete response = green, PR partial response = orange, SD stable disease = blue, PD progressive disease = red, NE not evaluated = grey) and events (alive = triangle, COVID-19 related death = square and melanoma death = circle).

We initially screened 10 patients with a *BRAF* VAF of ≥5% as the inclusion criteria, based on previous literature^17^ that this would identify patients with poorer prognosis disease with conventional treatment who might therefore benefit from alternative treatment strategies. Screening was performed analysing duplicate experiments using cfDNA pooled from 2 separate tubes taken at the same time, with a concordance of 0.99. We found that a cut-off of ≥5% *BRAF* VAF resulted in exclusion of some patients who still had poor prognostic disease (high LDH, multiple disease sites; Fig. 2a). Using the screening data collected (Supplementary Data), we reassessed the LLOD and LLOQ and determined that the inclusion VAF could be reduced to ≥1.5% without compromising the assay performance. Overall, analysis of the 44 screening samples showed that a threshold of ≥5% would lead to 45% of patients being included, whereas a ≥ 1.5% threshold would enable inclusion of 65% of patients (Fig. 2a). Despite lowering the threshold to ≥1.5% *BRAF* VAF, we still selected for a cohort which had features associated with a poorer prognosis compared to contemporary trials^4,9,18^ with the majority of patients having M1c disease, raised LDH and a median number of ≥3 disease sites (Table 1). Patients who were excluded due to a VAF of ≤1.5% were associated with lower baseline LDH (median 324.5 IU/L [interquartile range: 297.3–537.0] CAcTUS included patients (*n* = 21) vs. 218.0 [207.7–255.2] (*n* = 10) for <1.5% VAF excluded patients *p* = 0.0068 (Wilcoxon test)) with 76% (16/21) having >3 sites of disease for CAcTUS included patients vs. 30% (3/10) in those excluded due to <1.5% VAF.

**Table 1:** Patients’ characteristics at baseline

Variable	Level	Control arm (*N* = 10)	Experimental arm (*N* = 11)
Biological sex (*N*)	Male	6	6
	Female	4	5
Age (median [range])		62 [34–80]	67 [39–81]
ECOG (*N*)	0	6	7
	1	4	4
M classification (*N*)	Ia	2	2
	Ib	0	2
	Ic	7	7
	Id	1	0
Point Mutation (*N*)	V600E	9	9
	V600K	1	1
	V600R	0	1
No of disease sites (median [range])		4 [2–6]	3 [2–9]
RECIST sum of diameters in mm (median [range])		95 [49–241]	106 [51–158]
LDH (IU/L)	Normal	1	2
	≥x1ULN	6	6
	≥x2ULN	3	3
Albumin (g/L) (median [range])		44 [34–48]	45 [42–50]
CRP (mg/L) (median [range])^a^		20 [1–130]	5 [2–217]
ANC (10^9^/L) (median [range])		7.5 [1.1–10.9]	5.2 [3.4–12.5]
Lymphocyte (10^9^/L) (median [range])		1.2 [1–2.1]	1.4 [0.5–2.0]
ANC/Lymphocyte ratio (median [range])		5.5 [1.1–10.9]	3.4 [2.0–21.0]
Platelet (10^9^/L) (median [range])		339 [160–497]	253 [205–362]
ctDNA (*N*)	<1.5%	0	0
	1.5 to ≤10%	4	5
	>10 to ≤20%	3	0
	>20%	3	6
1st line treatment choice in control arm (*N*)	Dabrafenib + Trametinib	6	-
	Nivolumab + Ipilimumab	4	-

*ULN* upper limit of normal.

^a^One patient did not have baseline/screening for CRP.

Of note, the median % *BRAF* VAF was higher in the experimental arm (20.34% [range: 2.36–34.65%]) than control (13.4% [range: 2.53–63.48%]). In addition, in the control arm, the baseline *BRAF* VAF for those patients commencing CPI first was lower than those commencing TT (median 3.46% [range: 2.53–17.67%] vs. 16.91% [5.67–63.48%], respectively, *p* = 0.067), likely reflecting emerging evidence guidelines that CPI was associated with a better outcome for the majority of patients and first line targeted therapy should be reserved for those patients with symptomatic or poor prognosis disease requiring a rapid response to treatment.

Essential to the delivery of the study was the ability to report ctDNA results quickly. A key primary endpoint for feasibility was therefore to assess whether ≥95% of samples could be processed and reported following quality assurance approval within 7 working days. Of the 344 samples collected, 61 were designated as critical, i.e. would inform clinical decision-making. For these 61 samples, the mean processing time was 5.4 ± 1 working days (with median of 6 and range of 3–7 working days), with 100% (95% CI: 94–100%) critical results reported within 7 days (Fig. 2b).

The primary endpoint of CAcTUS was to assess whether we would observe an ≥80% decrease in mutant *BRAF* VAF. All the patients who initially received TT across both arms (control arm; *n* = 6, experimental arm; *n* = 11), were observed to have an ≥80% reduction in % *BRAF* VAF from baseline (95% CI: 80–100%), taking a median of 15 days (range: 13–54 days) to reach the 80% reduction threshold (Fig. 2c). In addition, all patients (*n* = 4) who initially received CPI, were observed to have an ≥80% reduction of % *BRAF* VAF with a median of 22 days to reach ≥80% reduction; range: 21–49 days (Fig. 2c). Of note, 3 of these 4 patients had a durable PR until trial end, suggesting the ctDNA reduction was associated with good outcomes to CPI. Of note, the progressing patient 18 had a rise in ctDNA before scan progression.

### Switch at time of ctDNA drop is associated with features of immunotherapy response

The treatments received and time on treatment are shown in the consort diagram (Fig. 1) and swimmers plot (Fig. 2d). Of the 6 patients who started targeted therapy first and who were treated to resistance on the control arm, 3 progressed on TT and received second line immunotherapy but all progressed quickly and died, 1 had progressive disease (PD) on TT and was not fit to receive CPI then died, and 2 patients responded until trial end. For those on the control arm who had CPI first, 3 had durable partial responses, and 1 progressed and switched to targeted therapy but subsequently further progressed. Of those on the experimental arm receiving initial treatment with D + T, 1 patient died due to disease progression before they could be switched, and 1 patient withdrew consent and switched to E + B due to toxicity and remained on this. The rest (*n* = 9/11) were switched from TT to CPI based on an ≥80% *BRAF* VAF drop. Of these 9 patients, 1 died from COVID-19, although was responding to treatment, 2 had a durable response to immunotherapy, and 6 subsequently progressed on CPI and switched back to targeted therapy. Two of these 6 patients died subsequently due to disease progression, and 4 were alive at trial end, with 2 continuing to respond to TT. Progression-free (including both TT and CPI treatment lines) and overall survival were not significantly different between both arms (median overall survival control arm 605 days [95% CI: 377–NR] vs. experimental arm 511 days [95% CI: 197–NR], *p* = 0.707 [Supplementary Fig. 1e, f]). Treatment-related toxicity was higher in the experimental arm than control, with 4 patients in the control arm and 9 in the experimental arm experiencing CTCAE v5 grade 3/4 toxicity. These were in line with the known toxicities for these regimens (Supplementary Tables 4–6).

**Fig. 3:**
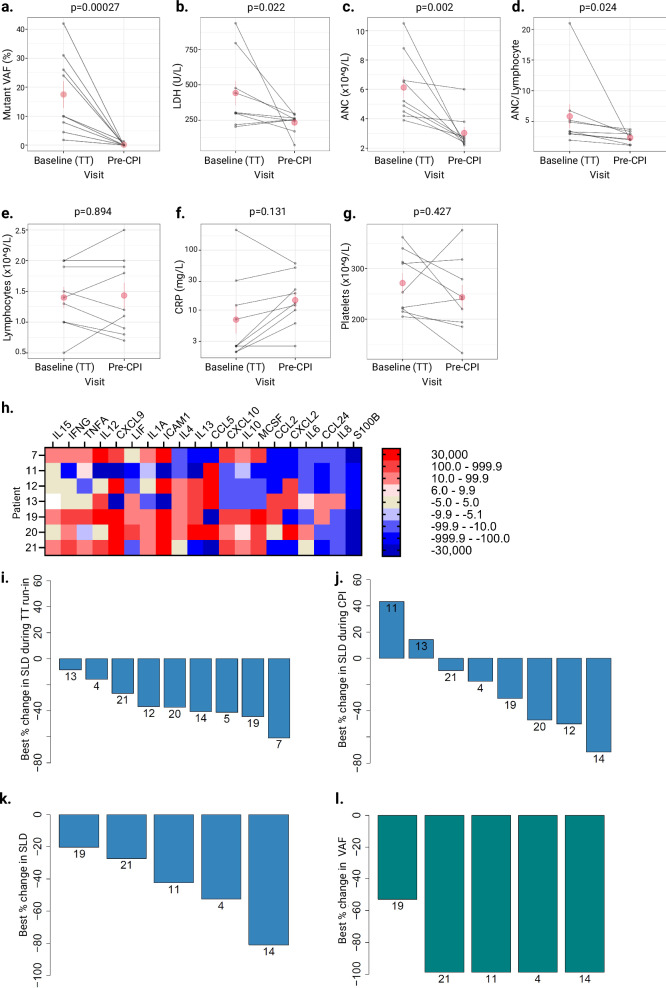
Switch at time of ctDNA drop is associated with features of immunotherapy response. **a**–**g** Difference in prognostic parameters between patients at C1D1 IO following the initial TT run-in vs. baseline in patients on the experimental arm. **a** ctDNA variant allele frequency (VAF). **b** Lactate dehydrogenase (LDH) **c**. Absolute neutrophil count (ANC) **d** ANC/lymphocyte ratio. **e** Lymphocyte count. **f** C-reactive protein (CRP). **g** Platelet count. Wilcoxon two-sided signed-rank test *p* values shown, *n* = 13 patients with 1 technical replicate, whisker plots represent the mean ± Standard Error of the Mean. **h** Heatmap showing difference in plasma pg/ml of circulating proteins between patients (numbered on each row, *n* = 7) at C1D1 IO following the initial TT run-in vs. baseline on the experimental arm. Red = increased and blue=decreased compared to baseline in patients on the experimental arm. Waterfall plots showing best % change in sum of lesion diameters (SLD) from **i** baseline to the switch (TT run-in, *n* = 9 patients) and **j** from the switch to end of trial or progression (CPI component, *n* = 8 patients) on the experimental arm. Waterfall plots showing best % change in **k** sum of lesion diameters (SLD, *n* = 5 patients) and **l** ctDNA VAF from C1D1 of restart of TT (following CPI progression, *n* = 5 patients) to end of trial or progression. Source data are provided as a Source data file.

To determine whether the targeted therapy run-in and switch at time of ctDNA drop could potentially affect response to immunotherapy, we examined features known to be associated with CPI response in patients on the experimental arm, from baseline to the start of immunotherapy. We observed a significant decrease in lactate dehydrogenase (LDH), neutrophils and neutrophil:lymphocyte ratio (Wilcoxon rank-sum test *p* < 0.05 for all), a non-significant decrease in platelets, and increase in C-reactive protein (CRP) as well as heterogeneous changes in lymphocytes (Fig. 3a–g). Furthermore, analysis of circulating cytokines/chemokines in patients on the experimental arm from baseline to cycle 1 day 1 of immunotherapy revealed an increase in a number of inflammatory mediators commonly associated with CPI response such as IFNg, TNFa, IL12, CXCL9, ICAM-1 and a reduction in those mediators associated with poorer outcomes such as IL6 and IL8 (Fig. 3h). We also observed a decrease in the melanoma-associated protein S100B, which correlated with the ctDNA decrease (Fig. 3h). When we compared those patients on the control arm commencing CPI first line with those patients on the experimental arm commencing CPI following the targeted therapy run-in, we observed lower LDH, neutrophils, platelets and neutrophil:lymphocyte ratio, with higher CRP (Supplementary Fig. 2a–f; statistical analysis not performed due to inadequate power) at the time of commencing CPI treatment in the experimental arm patients. Furthermore, we observed higher levels of circulating IFNg, CXCL10 and lower IL6 and IL8 in patients on the experimental arm vs. control arm prior to CPI (Supplementary Fig. 2g–r). To assess if the depth of radiological target lesion response reflected ctDNA response in the experimental arm, we examined the waterfall plots of best radiological response based on % change in sum of target lesion diameters (SLD) and the maximum ctDNA % change in VAF, as well as their best overall response determined by RECIST v1.1 evaluation (colour-coded bars). Of note, an additional scan was performed at the time of switch to CPI in the experimental arm. This revealed that the magnitude of ctDNA response (best % change in VAF) did not correlate well with the radiological response (best % change in SLD) due in part to the increased frequency of ctDNA assessments compared to scan assessments particularly in those patients with aggressive/poor prognosis disease (Supplementary Fig. 2s–v). To assess the kinetics of response to different treatment types in patients in the experimental arm, we assessed the best radiological response from baseline to the scan at switch (TT component) and from the scan at switch until PD or end of trial (CPI component). Excluding the 2 patients that progressed on immunotherapy, both components of the treatment strategy contributed to the response in the remaining patients (Fig. 3i, j). Finally, to assess whether retreatment with TT following progression on immunotherapy would restore disease control in the experimental arm, we assessed both the best radiological response (SLD) and the ctDNA response in patients restarting TT. We observed that all patients re-challenged with TT responded both radiologically and in ctDNA (Fig. 3k, l, Supplementary Table 7).

### Longitudinal ctDNA monitoring through multiple treatment lines provides early signals of treatment efficacy

**Fig. 4:**
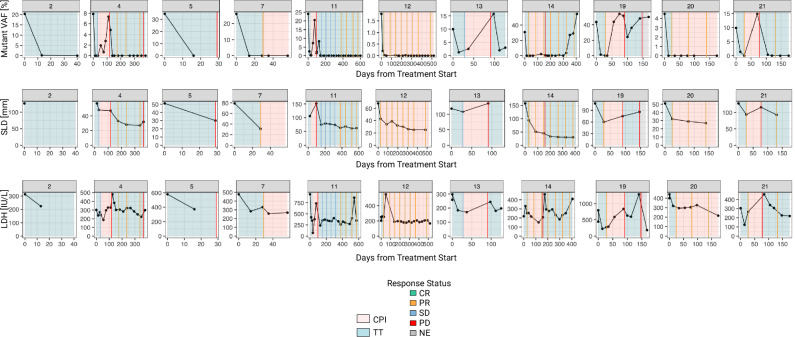
Circulating tumour DNA can track tumour response over multiple lines of treatment in the experimental arm. Levels of ctDNA, lactate dehydrogenase (LDH), sum of lesion diameters (SLD) and RECIST v1.1 response categories for each individual patient (total *n* = 11) in the experimental arm with treatment type overlaid (CPI vs. TT) and with vertical lines relating to overall visit response as per RECIST v1.1 evaluation. CR complete response = green, PR partial response = orange, SD stable disease = blue, PD progressive disease = red, NE not evaluated = grey. CPI: nivolumab plus ipilimumab. TT: dabrafenib plus trametinib.

To further assess the dynamics of ctDNA during treatment on each arm, we plotted the ctDNA level alongside the LDH and SLD (control arm Supplementary Fig. 3a, experimental arm Fig. 4). In both arms, ctDNA decrease was associated with response in SLD (e.g. patient 12, 15, 16 and 17). The ctDNA response sometimes preceded a CR in SLD (e.g. patient 10) and also differentiated true response from pseudoprogression as previously reported^19^. Patient 12 had an increase in size of a ureteric nodule, but the ctDNA remained <LLOD - this patient went on to have a metabolic CR on PET scan. We observed that ctDNA levels were a more accurate measure of tumour behaviour than LDH, which sometimes lagged behind (e.g. patient 8) or was increased due to other causes, e.g. patient 12 who had immune-related hepatitis at the time of LDH increase, but ctDNA/SLD remained in response. Of interest, the median time from start of TT until the ctDNA rise in the control arm was 156 days (range: 60–315) and therefore, for some patients, resistance would have occurred prior to the end of the 84-day TT induction period prescribed in EBIN^10^. In support of our washout strategy stopping trametinib 7 days prior and dabrafenib 1 day prior to switch to N + I in the experimental arm, we did not observe a rise in ctDNA during the washout period (Supplementary Fig. 3b). Critically, when switching to CPI in the experimental arm, we observed that ctDNA in the patients who did not respond to N + I rose quickly (e.g. patients 11, 13 and 21) following the switch, whereas in those who responded, the ctDNA level remained low (e.g. patients 7, 12, 20), suggesting that ctDNA levels could provide an early signal of efficacy of CPI in this context (Fig. 4, Supplementary Fig. 3b, Supplementary Table 8).

## Discussion

CAcTUS has shown that ctDNA can be used to prospectively guide clinical decision-making in melanoma with the aim of a ≤7-day turnaround of results. A number of retrospective studies have shown that it is possible to track response and resistance to therapy using ctDNA^13,15,20–22^. Here, we show that 100% of critical ctDNA results could be provided in a clinically relevant timeframe (≤7 working days) and acted upon quickly for treatment decision making, meeting our feasibility criteria (≥95% return of critical results within 7 days). Our choice of a ddPCR assay aided that turnaround, as it was simple to perform and did not require next-generation sequencing or an extensive bioinformatics pipeline. *BRAF* mutated melanoma is particularly suited to the tracking of one mutation, given that it is an essential driver in this disease, is rarely lost and therefore present in all clones^23,24^. In addition, we showed both in optimisation and during the trial that the assay was reproducible and repeatable, with the screening samples having a correlation of 0.99 between duplicate experiments. Although the breadth of information available from the test was limited, e.g. we could not identify early resistance mechanisms when patients were progressing on immune therapy, it was sufficient for this clinical application. In the future, it is possible that with more automation and the use of assays such as nanopore sequencing, which can be turned around in 24 h, a greater amount of data regarding subclonal resistance mechanisms could be inferred from each blood draw within a clinically relevant timeframe.

We showed that all patients who initially received TT across both arms (*n* = 17) had an ≥80% reduction of *BRAF* VAF, thus achieving the protocol-specified feasibility criteria. There were differences in patients 5, 11 and 18 in terms of the maximal ctDNA response and the RECIST/SLD response due to the timing of when tests were performed. Both patients 11 and 18 had a rise in ctDNA prior to the PD scan result, with patient 5 unevaluable due to further bloods being omitted because of their rapid deterioration. This suggests that ctDNA can capture very early responses even in those with rapidly resistant disease, due to the ability to assess more frequently than scans. However, as a marker of best overall response, it is less discriminatory than maximal percentage change of radiologically measured target lesions (SLD) and RECIST evaluation of best overall response (CR/PR/SD/PD). Therefore, the utility of ctDNA is likely to be greater in longitudinal monitoring of response and determining early molecular progression rather than as an overall response assessment. Larger studies are needed to assess how good a surrogate molecular PFS is at signalling potential OS benefit for different treatment modalities. Of note, although all the patients on the study initially had a ctDNA drop of ≥80% of *BRAF* VAF, the time to achieve that reduction ranged from 13 to 54 days. Although further validation in larger cohorts is required, these data suggest that achieving an initial ≥80% drop in *BRAF* VAF (regardless of time to that drop) could be an important target for new treatments seeking an early signal of efficacy in advanced melanoma. Furthermore, additional biomarkers of tumour burden or those associated with immune activation within a composite multiparameter assay may improve the ability to more accurately predict response.

To enable the strategy of ctDNA-informed treatment switch, we required sufficient amounts of ctDNA present at baseline to accurately measure the ≥80% drop in *BRAF* VAF. However, the use of a ≥5% VAF inclusion greatly reduced the number of patients eligible for the trial and selected for patients with more aggressive disease and poorer outcomes. Reducing the inclusion criteria to ≥1.5% still enabled us to accurately quantify the drop, and this resulted in a more representative patient population. Assays with greater sensitivity might increase the dynamic range to enable the VAF inclusion criteria to be reduced further. Alternatively, we have found that when using a ddPCR assay, quantifying the amount of ctDNA present as mutant copies/ml plasma results in a lower variance when there are low levels of ctDNA and a greater dynamic range as the relative nature of VAF constrains its range to typically a fraction of a % up to a few tens of percent, whereas copies/ml can range from one to thousands (Supplementary Fig. 4). Further, using copies/ml is less confounded by fluctuations in the background of normal cell free DNA. We have utilised this in the DyNAMIc trial (NCT06470880), which uses ctDNA levels to guide an adaptive treatment strategy using TT to delay the emergence of resistance. This trial will also better define thresholds of increased disease activity and resistance on targeted therapy in advanced melanoma.

The DREAMSeq study showed that for the majority of eligible patients, first-line combination immunotherapy with N + I is superior to D + T^4^. However, within the first year of treatment, there were some patients who had a worse outcome with CPI compared to those receiving TT. This indicates that there are some patients, often with rapidly progressive disease and other adverse prognostic features, who respond poorly to immunotherapy and obtain a greater benefit from TT, albeit relatively short-lived. The EBIN study further characterised a group of patients, i.e. those with liver metastasis and LDH ≥x2 ULN, who may benefit from a brief treatment with TT prior to an elective switch to immunotherapy^10^. PFS and OS were not significantly different in both arms in our study; however, these analyses were limited by small numbers as well as the impact of COVID-19. We have shown that the majority of patients had a reduction in ctDNA of ≥80% within 2 weeks of starting TT. This was reflected in a reduction in several other adverse features (LDH, NLR, IL6, IL8), which usually predict a poor response to CPI^25–27^. This suggests that a short induction treatment with TT may result in rapid tumour control, modifying immunosuppressive features and potentially providing symptomatic benefit. For the majority of patients, this is 2–3 weeks of treatment, which is much shorter than the EBIN and SECOMBIT studies^9,10^. For some patients, the potential window where targeted therapy is controlling the tumour and priming the immune system is likely short. Given the small sample size and exploratory nature of these analyses, statistically significant findings should be interpreted cautiously. These results are intended to inform hypothesis generation for future validation, rather than to support confirmatory inference. Although CAcTUS was designed around technical endpoints, it highlights the utility of ctDNA to optimise scheduling treatment strategies before expanding to larger cohorts where correlations with patient outcomes can be tested.

Using a short, ctDNA-guided, targeted therapy induction, we were able to switch 9/11 patients with poor-prognosis disease (1 patient died before the switch) to CPI. Therefore, this short TT induction in patients with symptomatic disease, who would benefit from rapid disease control, reassuringly does not prevent them from being able to receive CPI. We also found that a proportion of patients responding to induction TT in the experimental arm had a further radiological response in target lesions to CPI. This suggests a potential additive benefit when switching to CPI in patients responding to TT. However, not unexpectedly, a number of those responding to TT progressed when switched to CPI; this was also seen in approximately 30% of patients in the SECOMBIT and EBIN studies^9,10^. This CPI primary refractory group were not able to be converted to responders by the TT induction and therefore needs alternative strategies. The small number of patients included means we are not able to comment on whether there was any clinical benefit with the short induction treatment. In the EBIN study, there was a clear advantage in terms of PFS at 12 weeks; the PFS was 99% (95% CI: 95–100%) in the induction arm versus 73% (95% CI: 64–80%) in the control arm. At 6 months, when patients in the induction arm had switched from TT to CPI, the estimated difference between the two arms was smaller than initially, with a PFS rate of 62% (95% CI: 53–70%) versus 56% (95% CI: 47–64%)^10^. The estimated curves crossed around the 10-month mark^10^. Of interest, some patients, e.g. 11 progressing on CPI, subsequently achieved a good PFS benefit from TT rechallenge, despite high baseline VAF levels suggesting a poor prognosis. We observed a rise in ctDNA in all of the CPI non-responders at a median of 41 days (range: 21–84, Supplementary Table 8), which was confirmed radiologically. This suggests that ctDNA may identify patients not responding to CPI earlier than conventional imaging, enabling a rapid switch to alternative treatments if available, though further trials are needed to assess this.

The CAcTUS Study has shown that ctDNA can be utilised prospectively for clinical decision-making in advanced melanoma, and results can be provided in a clinically meaningful timeframe to inform treatment decisions. In addition, we showed that an ≥80% decrease in *BRAF* VAF is a realistic target to determine ctDNA response in first-line advanced melanoma treatment. Through longitudinal tracking of *BRAF* VAF levels, we have gained insights on the rapidity of response to TT, how poorer prognosis patients could potentially be optimised for CPI and have shown that individualised treatment decisions can be made based on ctDNA changes. Furthermore, ctDNA identified early those patients who were not responding to immunotherapy and captured their response to TT rechallenge. Thus, ctDNA can be used prospectively throughout multiple treatment lines to provide information on tumour activity in a clinically meaningful timeframe.

## Methods

### Trial oversight

The study design and conduct complied with all relevant regulations regarding the use of human study participants and was conducted in accordance with the criteria set by the Declaration of Helsinki. The protocol and amendments for this trial were reviewed by the North West Greater Manchester Central research ethics committee and were granted favourable opinion (ref. ^19^/NW/0046). The trial was conducted in accordance with Good Clinical Practice guidelines and the Medicines for Human Use (Clinical Trials) Regulations 2004 (SI 2004/1031), amended regulations (SI 2006/1928). All patients provided written informed consent before enrolment. The Manchester Clinical Trials Unit were assigned to oversee the trial by the study Sponsor, The Christie NHS Foundation Trust. Additional oversight was provided by the Trial Management Group and the Trial Steering Committee, which also covered the remit of an Independent Data Monitoring Committee. Additional ethical approval for the translational analysis was obtained through the Manchester Cancer Research Centre (MCRC) Biobank ethics application 23_RELE_01 and approval for the work under MCRC Biobank Access Committee application ref: 22/NW/0237. The trial was registered on Clinicaltrials.gov NCT03808441.

### Patients

Eligible patients were aged ≥18 years with histological confirmation of stage III unresectable/IV cutaneous melanoma. A *BRAF* p.V600E/K/R mutation confirmed in tissue, with the exact point mutation known. Patients had to have at least one target lesion measurable by CT or MRI as per RECIST 1.1, adequate organ function and Eastern Cooperative Oncology Group (ECOG) performance status 0/1/2. Prior radiotherapy or radiosurgery must have been completed at least 2 weeks prior to the first dose of study drug. Patients had to have a *BRAF* VAF level of ≥5% when the trial was initiated; however, this was changed to ≥1.5% following a review of the data from the first 10 patients screened. Patients of any sex could be included (determined based on self-report); however, due to the size of the study, no specific analyses based on sex were carried out. Key exclusion criteria included patients with a condition requiring systemic treatment with either corticosteroids (>10 mg daily prednisone equivalent) or other immunosuppressive medications within 14 days of study drug administration. Patients with brain metastases and leptomeningeal metastases were excluded unless asymptomatic and untreated at presentation, or if *s*ymptomatic, the lesions had to have been definitively treated with surgery or stereotactic surgery and did not require steroids for control of symptoms. See protocol (Supplemental information) for other exclusion criteria. Patients were enrolled between 2nd May 2019 and 30th May 2022, and the trial completed when the last patient finished their follow-up.

### Trial design and endpoints

CAcTUS was designed as a parallel arm randomised phase II, multicentre, feasibility trial. It was conducted across 12 UK centres. Initially, 40 patients were planned to be randomised 1:1 to either control arm (investigator choice) or experimental arm (ctDNA-guided switch). The primary feasibility endpoints of the study were i. ≥95% return of critical (red) ctDNA results returned within 7 working days of samples being received in the laboratory ii. ≥80% of patients with a decrease in ctDNA level of mutant BRAF ≥ 80% on targeted therapy. The number of patients was later reduced to 21, as in discussion with the trial steering committee, we observed that all patients commencing TT were reaching the primary endpoint of 80% VAF drop, and therefore the trial was closed to recruitment. The original study sample size target was 40 patients (20 in each arm) based on true feasibility sample size parameters of a *i*. 95% (or greater) return rate of critical clinical decision dependent blood samples; *ii*. 80% (or greater) success rate of participants on targeted therapy achieving ≥80% *BRAF* VAF decrease in ctDNA levels enabling estimation of **i**. the return rate with a two-sided 95% confidence interval of width no more than 12% points (i.e. 87–99%) assuming at least 60 critical red blood samples (one sample from each of the 20 control arm participants and an average of at least two samples from each of the 20 the experimental arm participants); **ii**. the success rate of participants on targeted therapy achieving ≥80% *BRAF* VAF decrease in ctDNA levels with a two-sided 95% confidence interval of width equal to 30% points (i.e. 62%, 92%), assuming that 25% of the participants in the control arm will receive D + T as first line treatment (i.e. 25 participants across arms; 5 in the control arm and 20 in the experimental arm). On interim review of the return rate, there was good evidence of a high return rate, and hence a 95% confidence interval of similar width (14%) could be obtained from a sample of 21 or more participants (resulting in at least 32 critical clinical decision dependent samples, assuming one sample from control arm participants and at least two samples from experimental arm participants). Moreover, 32 samples provided 80% confidence that the lower confidence limit was at least 90% if the true return rate was at least 98%. Likewise, with a sample size of 17 participants with ctDNA reduction data, we could be confident that at least 80% of participants would achieve a reduction in ctDNA levels of at least 80% from baseline, assuming that the true ‘success rate’ percentage was 95% (or greater).

Randomisation was performed centrally via the King’s College London independent randomisation service. Patients were equally randomised to either treatment arm using minimisation with a random element controlling for LDH (<ULN vs. ≥ULN), disease site (<3 sites vs. ≥3 sites), ctDNA VAF (1.5 to ≤10%, >10 to ≤20%, >20%) and M stage (stage III unresectable plus M1a, M1b, M1c and M1d).

In the control arm, the choice was between either dabrafenib 150 mg twice daily plus trametinib 2 mg once daily or nivolumab 1 mg/kg plus ipilimumab 3 mg/kg Q3wk followed by 480 mg nivolumab Q4wk first line, then switching on progression to the other treatment second line until disease progression or unacceptable toxicity. The experimental arm commenced dabrafenib 150 mg twice daily with trametinib 2 mg once daily. Following decrease of ctDNA *BRAF* VAF by ≥80%, trametinib was stopped for 7 days with dabrafenib continuing until the day before switching treatment. A CT scan was performed to correlate radiological imaging with ctDNA response. The patient was then switched to nivolumab 1 mg/kg plus ipilimumab 3 mg/kg Q3wks followed by 480 mg nivolumab Q4wk. Then, if progression was observed, they were switched back to dabrafenib 150 mg twice daily plus trametinib 2 mg once daily until disease progression or unacceptable toxicity.

The co-primary endpoints were the number of critical (defined as required for a clinical decision) ctDNA results returned within 7 working days of samples being received in the laboratory and the number of patients commencing TT first in whom we observed an ≥80% decrease in *BRAF* VAF.

### Assessments

Response and disease progression were assessed by radiologists at each site according to RECIST guidelines (version 1.1). Toxicity was assessed according to Common Terminology Criteria for Adverse Events guidelines (CTCAE version 5).

### Collection of blood samples and plasma separation

Blood samples were collected at pre-specified timepoints according to the protocol schedule (Supplemental information). Four tubes of 10 ml of venous blood were collected into Streck Cell-Free DNA BCT tubes and transported at ambient temperature. For ctDNA assays, double spun (DS) plasma was separated within 96 h of blood draw by centrifugation at 2000 × *g* for 10 min, followed by a second centrifugation at 2000 × *g* for 10 min, then stored at −80 °C until required. For Luminex assays, double spun plasma from EDTA tubes was separated within 1 h by centrifugation at 2000 × *g* for 10 min, followed by a second centrifugation at 2000 × *g* for 10 min and stored at −80 °C until required.

### Droplet digital PCR

Cell-free DNA was isolated from the entire DS plasma using the QIAsymphony DSP DNA Kit (Qiagen, Cat No: 1091063) as per the manufacturer’s instructions. cfDNA was isolated from 6 ml batches of DS plasma, each eluted in 60 μl of kit elution buffer (Qiagen). These were pooled and stored at −20 °C until required.

ddPCR was performed using the QX200-AutoDG Droplet Digital PCR System (Bio-Rad) as per the manufacturer’s instructions. All preparation and reaction set-up was performed in a dedicated pre-PCR room and PCR cabinet. One of three *BRAF* 20x primer-probe mutation detection assays (Bio-Rad) was used to measure %VAF; *BRAF* p.V600E c.1799T>A (Assay ID: dHsaMDV2010027), *BRAF* p.V600K c.1798_1799GT>AA (Assay ID: dHsaMDV2010035), and *BRAF* p.V600R c.1798_1799GT>AG (Assay ID: dHsaMDV2010037).

To improve quantification of the 80% drop in %VAF from screening/baseline (i.e. clinical decision making), critical timepoints (D + T week 2 and D + T week 4) were run in 8 wells. Other timepoints were run in 4 wells. Each 22 μL ddPCR reaction contained 11 μL of 2x ddPCR SuperMix for probes (no dUTP) (Bio-Rad), 9.9 μL of template DNA, and 1.1 μL of 20x primer-Probe mutation detection assays (Bio-Rad). Reactions were prepared in Bio-Rad ddPCR 96-well plates and droplets generated using the AutoDG. Plates were sealed with a pierceable foil heat seal (Bio-Rad) and PCR performed on a C1000 Touch thermal cycler (Bio-Rad) as per the manufacturer’s instructions. Amplifications were performed using the following cycling conditions: 1 cycle of 95 °C (2.5 C/s ramp) for 10 min, 40 cycles of 94 °C (2.5 C/s ramp) for 30 s and 55 °C for 1 min, followed by 1 cycle of 98 °C (2.5 C/s ramp) for 10 min. The sample was held at 4 °C until further processing. After PCR, the plates were read using a QX200 droplet reader (Bio-Rad).

Positive and negative controls were used to manually set thresholds, which were applied to all samples in the assay. QuantaSoft™ software (version 1.7.4) was used to assign positive/negative droplets. Counts were then converted to copies/ml and %VAF. The %VAF was quantified if the sample contained more than 10 mutant droplets and recorded as detected is it contained between 3 and 9 mutant positive droplets. Samples containing <3 mutant positive droplets were below the limit of detection.

The entire sample analysis workflow was validated and performed to ICH GCP E6, and a quality-assured %VAF result was reported.

### Luminex analysis

Analysis was performed according to manufacturer’s instructions using the human Luminex Discovery assay panels on an xMAP INTELLIFLEX^®^ machine. Analysis was performed using Quantist v1.0.1. Further analysis/heatmap visualisation was performed using Prism v10.2.2.

### Statistical analysis, visualisations, and data engineering

Data engineering was performed using R packages tableone (v0.13.2), tidyverse (v2.0.0), and lubridate (v1.9.2). Data visualisations were generated using ggplot2 (v3.4.2). For statistical analyses, differences in central tendency between groups or timepoints were assessed using Wilcoxon rank-sum test or a Wilcoxon signed rank test when values could be paired. All biomarker and exploratory efficacy analyses were hypothesis-generating. Unadjusted *p* values are reported descriptively alongside effect sizes and confidence intervals; no formal adjustment for multiple testing was applied.

### Reporting summary

Further information on research design is available in the Nature Portfolio Reporting Summary linked to this article.

## Supplementary information

Supplementary Information

Description of Additional Supplementary Information

Supplementary Data 1 and 2

Reporting Summary

Transparent Peer Review File

## Source data

Source data

## Data Availability

The study protocol is available in the Supplementary Information. Individual patient-level clinical data cannot be provided for ethical reasons. Sharing of additional clinical data not within the manuscript or in supplementary materials will be considered upon request to the Sponsor (The Christie NHS Foundation Trust) and Chief Investigator Prof. Paul Lorigan. The remaining data are available within the article, Supplementary Information or Source data file. Source data are provided with this paper.

## Code availability

The code is available here: 10.5281/zenodo.19337944.
