# Supplementary material for: Use of circulating tumour DNA to prospectively guide a switch from targeted to immune therapy in BRAF mutant advanced melanoma: the randomised phase II CAcTUS trial
Source: Nat Commun. 2026 May 21;17:6850. doi: 10.1038/s41467-026-72735-8 (PMC13389006; doi:10.1038/s41467-026-72735-8)
Supplement: Supplementary file 1 — Supplementary Information [file 41467_2026_72735_MOESM1_ESM.pdf]

Supplementary Figure 1.

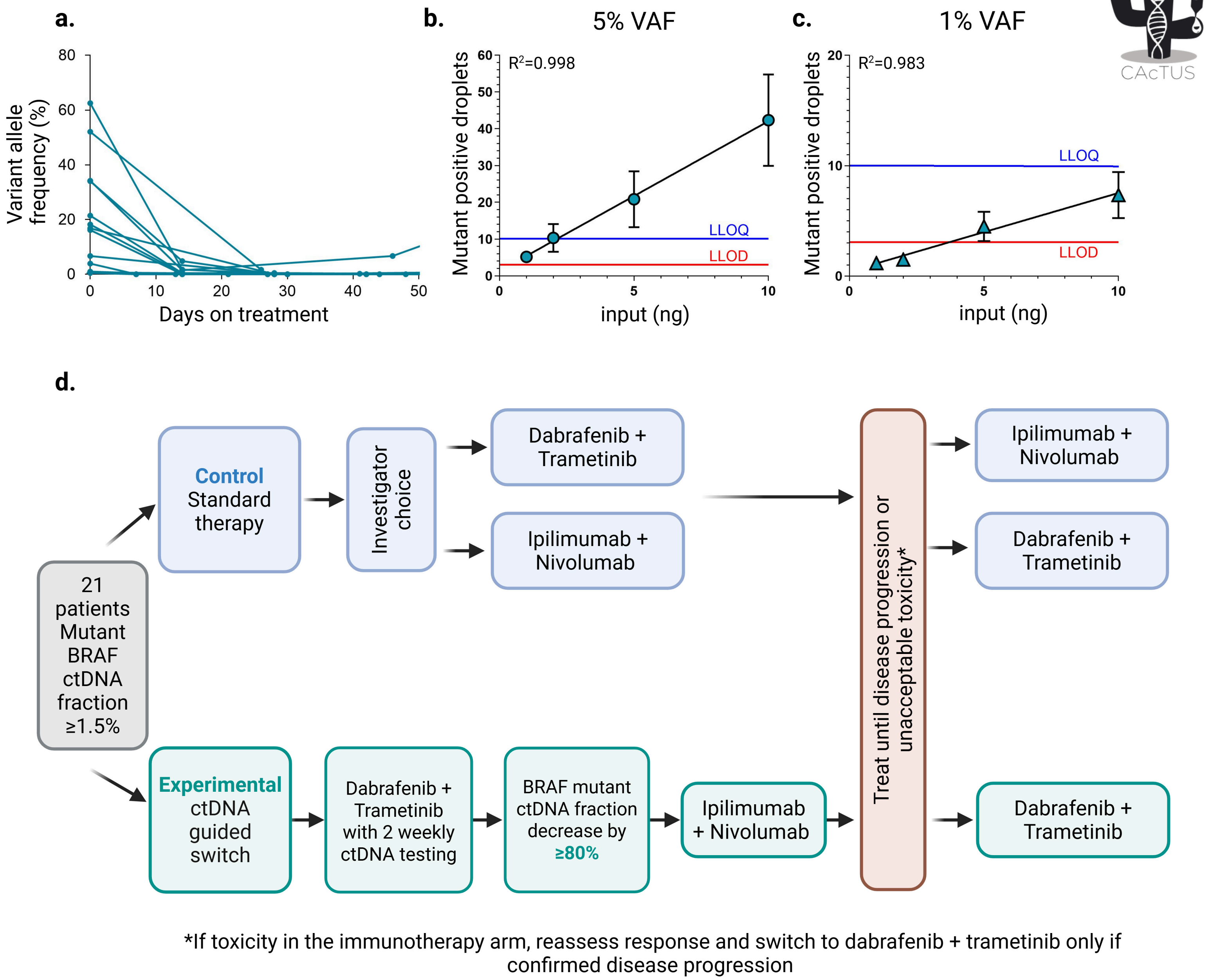

Clinical trial delivery and outcomes

a. Retrospective ddPCR analysis of *BRAF* mutation in ctDNA showing that the majority of patients are observed to have an ≥80% decrease in VAF from baseline when treated with targeted therapy (dabrafenib n=13, dabrafenib + trametinib n=8). **b&c.** Standard curves of number of mutant droplets generated depending on DNA inputs at **b.** 5% (the initial inclusion criteria) equation of the line of best fit $y=4.068x + 1.361$, $R^2=0.998$ and **c.** 1% VAF equation of the line of best fit $y=0.7092x + 0.4337$, $R^2=0.983$. LLOQ and LLOD are indicated on each graph with blue and red lines respectively. The equation of the line of best fit was used to extrapolate input amounts required for ≥3 and ≥10 mutant positive droplets at each VAF%. Source data are provided as a Source Data file, n=3 replicates.

d. Trial schema. Patients were randomised to Control Arm investigator choice nivolumab 1mg/kg when combined with ipilimumab 3mg/kg 3 weekly for 4 doses followed by nivolumab maintenance 480mg 4 weekly (N+I) or dabrafenib 150mg twice daily plus trametinib 2mg once daily (D+T). Upon progression they could switch to the opposite therapy. Experimental Arm patients commenced D+T then when ctDNA had dropped by 80%, they switched to N+I. Patients could switch back to D+T upon progression. Created in BioRender. Lee, R. (2026) https://BioRender.com/0lgnfrz

Supplementary Figure 1. continued

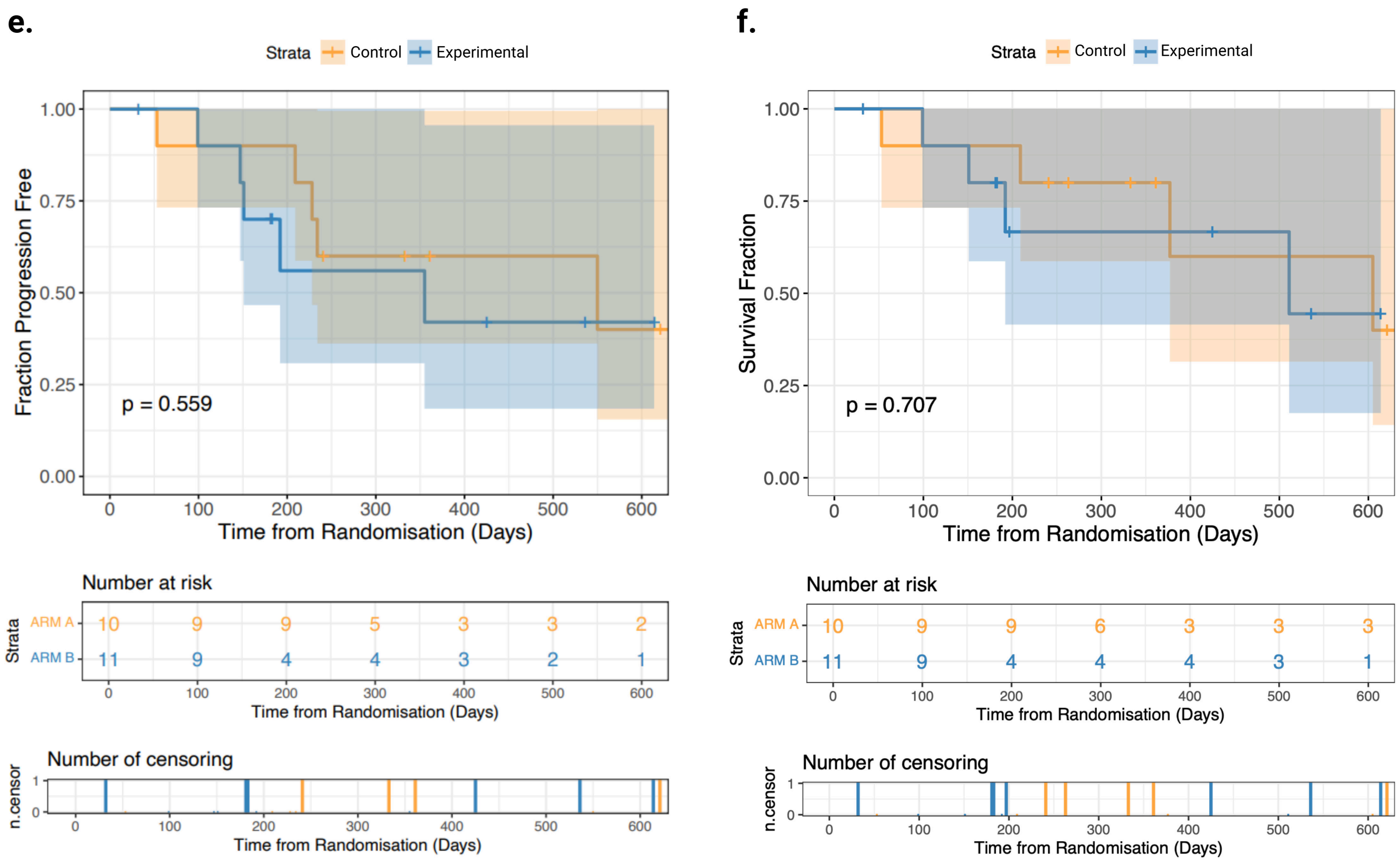

Kaplan Meier estimate of patients on Control Arm (orange, n=10) vs. Experimental Arm (blue, n=11) **e.** progression-free survival of the entire intended treatment (Control Arm either CPI or TT followed by CPI or TT; Experimental Arm TT run-in then switch to CPI then TT) **f.** overall survival. Differences in survival determined by log-rank test.

Supplementary Figure 2.

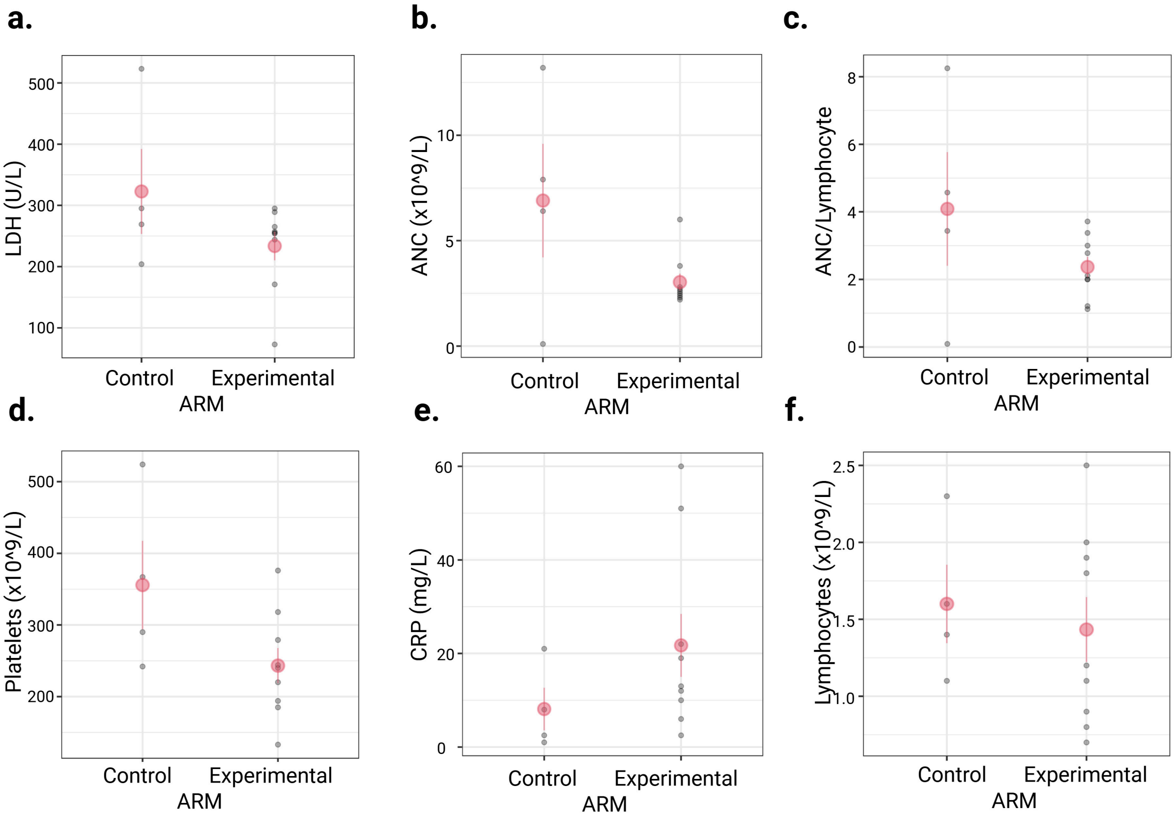

Association of prognostic variables in control vs. experimental arm commencing CPI. Difference in prognostic parameters between patients starting CPI first on the control arm (n=4) with C1D1 immune therapy of the experimental arm (n=9) following the initial TT run-in.

a. Lactate dehydrogenase (LDH)

b. Absolute neutrophil count (ANC) **c.** ANC/lymphocyte ratio

d. Platelet count

e. C-reactive protein (CRP)

f. Lymphocyte count.

g-r. Difference in prognostic parameters between patients starting CPI first on the control arm (n=4) with C1D1 immune therapy of the experimental arm (n=7) following the initial TT run-in. Source data are provided as a Source Data file.

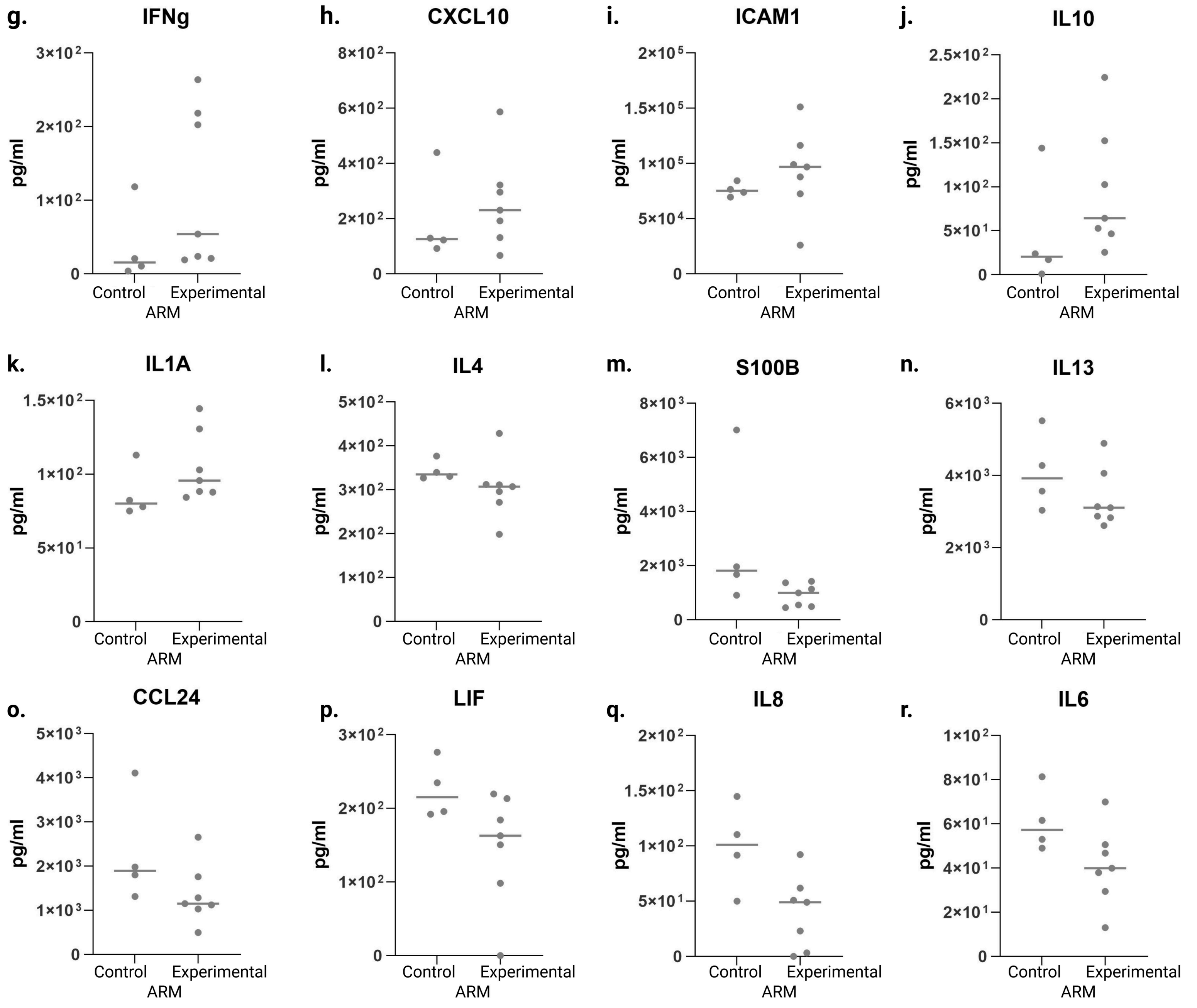

Supplementary Figure 2. continued

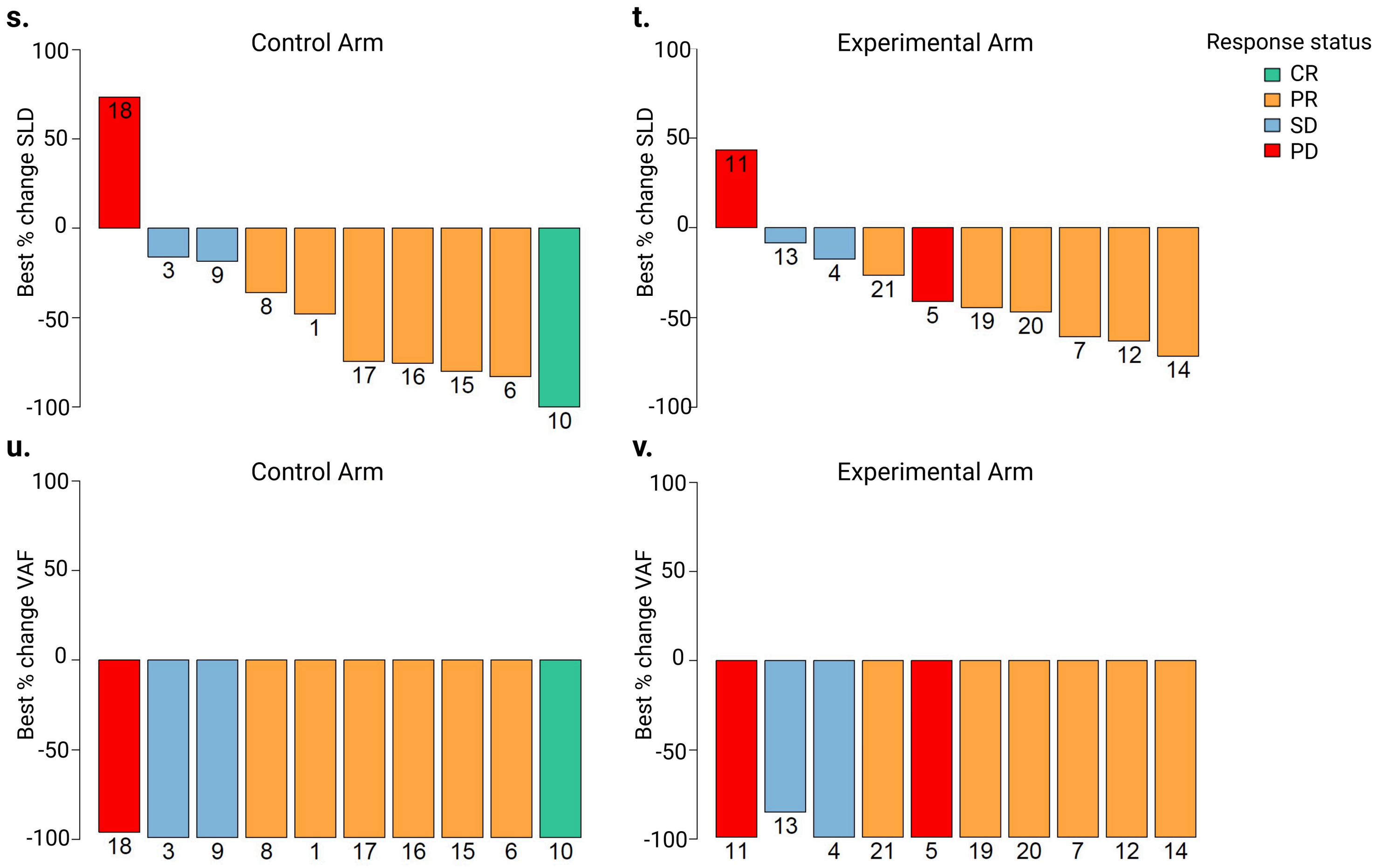

s-v. Waterfall plots comparing best radiological response; % change in sum of target lesion diameters (SLD) in **s**. Control Arm (n=10) and **t.** Experimental Arm (first-line=TT run-in + CPI, n=10) compared to best ctDNA response (VAF) in **u.** Control Arm (n=10) and **v.** Experimental Arm (n=10). Of note, patient 5 had new lesions on scan therefore despite decrease in the target lesions, had PD.

Supplementary Figure 3.

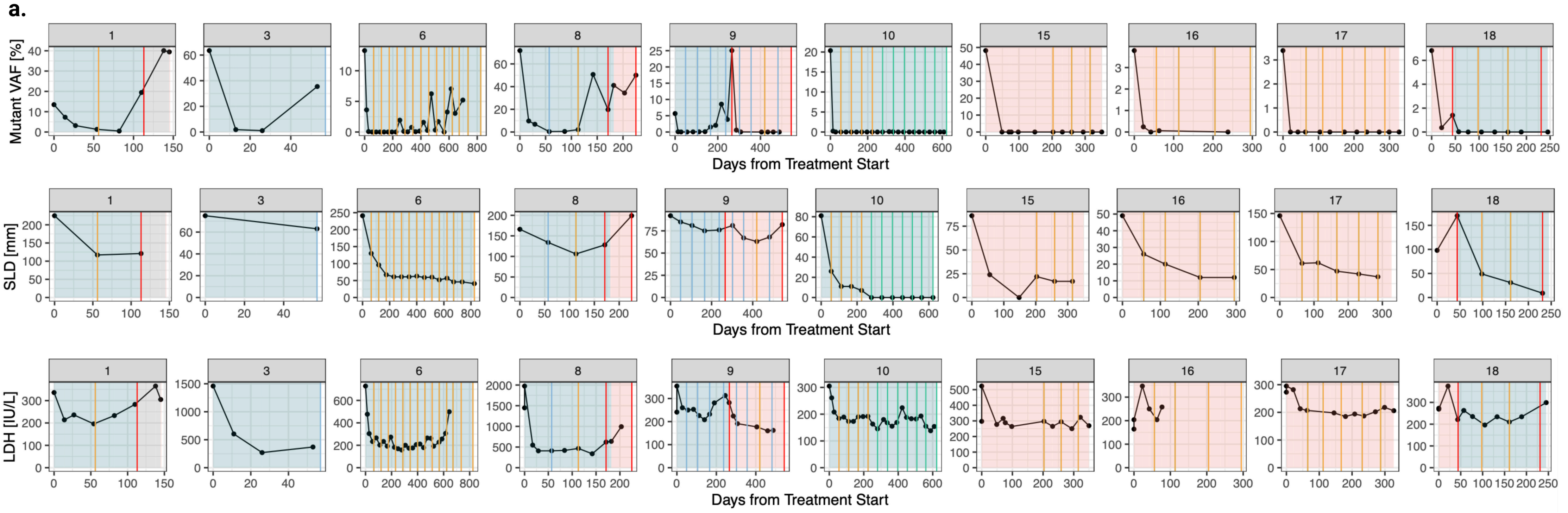

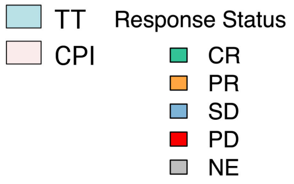

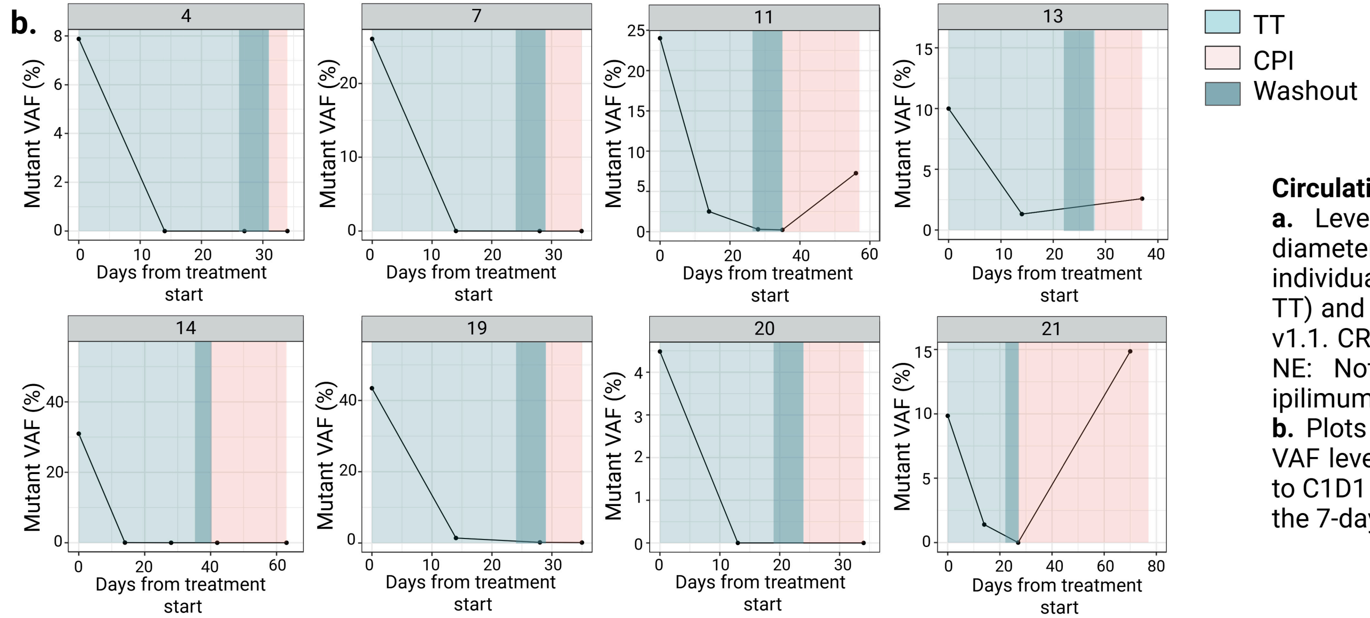

Circulating tumour DNA can track tumour response

a. Levels of ctDNA, lactate dehydrogenase (LDH), sum of lesion diameters (SLD) and RECIST v1.1 imaging response categories for each individual patient in Control Arm (n=10) with treatment type (CPI and TT) and vertical lines relating to overall imaging response as per RECIST v1.1. CR: Complete Response, PR: Partial Response, SD: Stable Disease, NE: Not Evaluated, PD: Progressive Disease. CPI: nivolumab plus ipilimumab. TT: dabrafenib plus trametinib.

b. Plots for individual patients in Experimental Arm (n=8) showing the VAF level change from the 80% drop timepoint (i.e. the switch decision) to C1D1 of CPI (middle dark blue shaded region in each plot relates to the 7-day washout period).

Supplementary Figure 4.

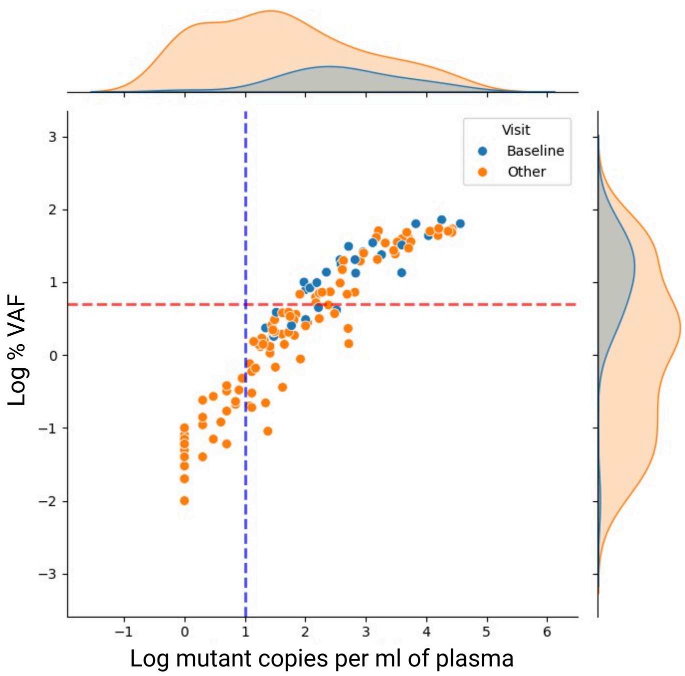

Plot of circulating tumour DNA log % VAF compared to log mutant copies per millilitre (ml) of plasma (n=262 samples from n=21 patients) with marginal distributions shown. Vertical blue line shows 10 copies/ml, horizontal orange line shows 5% VAF.

Supplementary tables

Supplementary table 1. Summary of the specificity results

ddPCR Assay	Control Cell Line	A375	RM44	M381	RM59
	Mutation Status	(BRAF V600E)	(BRAF V600K)	(BRAF V600R)	(BRAF V600WT)
BRAF V600E	Determined %VAF	100.00	0.00	0.00	0.00
	SD %VAF	0.00	0.00	0.00	0.00
	Acceptance Range	>90%	<1%	<1%	<1%
	Outcome	Pass	Pass	Pass	Pass
BRAF V600K	Determined %VAF	0.00	78.77	0.00	0.00
	SD %VAF	0.00	0.54	0.00	0.00
	Acceptance Range	<1%	>75%, <85%	<1%	<1%
	Outcome	Pass	Pass	Pass	Pass
BRAF V600R	Determined %VAF	0.00	0.00	33.48	0.00
	SD %VAF	0.00	0.00	1.76	0.00
	Acceptance Range	<1%	<1%	>28%, <38%	<1%
	Outcome	Pass	Pass	Pass	Pass
BRAF V600plex (E + K +R)	Determined %VAF	100.00	78.39	32.15	0.00
	SD %VAF	0.00	0.85	1.78	0.00
	Acceptance Range	>90%	>75%, <85%	>28%, <38%	<2%
	Outcome	Pass	Pass	Pass	Pass

Three replicates of each QC (cell line) for each assay, performed in three experiments.

Supplementary table 2. Summary of reproducibility and repeatability results with the acceptance criteria

Sample	ddPCR Assay		Experiment Number			Inter-assay %VAF
			359782009	359782010	359782011	
A375 (BRAF V600E)	BRAF V600E	Determined %VAF	100.00	100.00	100.00	100.00
		Measured Range	100.00 to 100.00	100.00 to 100.00	100.00 to 100.00	100.00 to 100.00
		Acceptance Range*	95.00 - 100.00	95.00 - 100.00	95.00 - 100.00	95.00 - 100.00
		Outcome	Pass	Pass	Pass	Pass
RM44 (BRAF V600K)	BRAF V600K	Determined %VAF	78.50	79.39	78.43	78.77
		Measured Range	75.89 to 80.21	77.67 to 80.66	77.91 to 78.72	78.43 to 79.38
		Acceptance Range*	73.77 - 83.77	73.77 - 83.77	73.77 - 83.77	73.77 - 83.77
		Outcome	Pass	Pass	Pass	Pass
M381 (BRAF V600R)	BRAF V600R	Determined %VAF	33.51	31.70	35.22	33.48
		Measured Range	30.06 to 35.26	30.84 to 32.36	34.73 to 35.64	31.70 to 35.22
		Acceptance Range*	28.48 - 38.48	28.48 - 38.48	28.48 - 38.48	28.48 - 38.48
		Outcome	Pass	Pass	Pass	Pass
A375 (BRAF V600E)	BRAF V600plex (E + K +R)	Determined %VAF	100.00	100.00	100.00	100.00
		Measured Range	100.00 to 100.00	100.00 to 100.00	100.00 to 100.00	100.00 to 100.00
		Acceptance Range*	95.00 - 100.00	95.00 - 100.00	95.00 - 100.00	95.00 - 100.00
		Outcome	Pass	Pass	Pass	Pass
RM44 (BRAF V600K)	BRAF V600plex (E + K +R)	Determined %VAF	78.10	79.35	77.72	78.39
		Measured Range	77.45 to 78.72	77.84 to 81.18	76.24 to 79.36	77.72 to 79.35
		Acceptance Range*	73.77 - 83.77	73.77 - 83.77	73.77 - 83.77	73.77 - 83.77
		Outcome	Pass	Pass	Pass	Pass
M381 (BRAF V600R)	BRAF V600plex (E + K +R)	Determined %VAF	32.97	33.37	30.11	32.15
		Measured Range	29.37 to 35	30.38 to 36.13	27.38 to 31.97	30.11 to 33.37
		Acceptance Range*	28.48 - 38.48	28.48 - 38.48	28.48 - 38.48	28.48 - 38.48
		Outcome	Pass	Pass	Pass	Pass
*Acceptance Range is ±5 of the determined inter-assay %VAF for that QC / Assay						

Three replicates of each QC (cell line) for each assay, performed in three experiments.

Supplementary table 3. Number of mutant positive droplets generated for known VAF controls with reducing cfDNA inputs

	Number of Mutant Positive Droplets at various cfDNA input concentrations			
**% VAF**	**10 ng**	**5 ng**	**2 ng**	**1 ng**
**20%**	161	91	36	15
**10%**	73	43	22	8
**5%**	42	21	10	5
**1%**	7	5	2	1
**0%**	0	0	0	0
Values in green are ≥10 Mutant Positive Droplets				
Values in Orange are ≥3 but <10 Mutant Positive Droplets				
Values in Red are <3 Mutant Positive Droplets				

A single replicate at each input / %VAF was run in two experiments.

Supplementary table 4. Overall CTCAE treatment related graded toxicities by treatment arm

**Adverse event**	**Arm A (n=10)**		**Arm B (n=11)**	
	**Any grade**	**Grade 3 or 4**	**Any grade**	**Grade 3 or 4**
**Dry skin**	0	0	1	0
**Maculopapular rash**	2	0	3	1
**Pruritus**	1	0	2	0
**Rash**	3	0	2	0
**Vitiligo**	1	0	0	0
**Hyperthyroidism**	3	0	1	0
**Hypothyroidism**	1	0	2	1
**Abdominal pain**	1	0	0	0
**Constipation**	4	0	2	0
**Diarrhoea**	5	1	4	0
**Nausea**	3	0	3	0
**Vomiting**	1	0	4	0
**Weight loss**	0	0	1	0
**Anaemia**	1	0	0	0
**Hyperglycaemia**	1	1	0	0
**Hyperkalaemia**	0	0	1	0
**Decrease LVEF**	1	1	0	0
**Hypotension**	1	0	1	1
**Elevated ALT**	1	1	2	2
**Elevated AST**	0	0	3	2
**Headache**	3	0	3	0
**Arthralgias**	0	0	1	0
**Dyspnoea**	1	0	1	1
**Fatigue**	4	0	6	0
**Fever**	6	0	4	1

Supplementary table 5. Overall CTCAE v5 targeted treatment related graded toxicities by treatment arm

Adverse event	Arm A (n=10)		Arm B (n=11)	
	Any grade	Grade 3 or 4	Any grade	Grade 3 or 4
**Rash**	1	0	0	0
**Constipation**	3	0	1	0
**Diarrhoea**	1	0	2	0
**Nausea**	3	0	2	0
**Vomiting**	1	0	4	0
**Weight loss**	0	0	1	0
**Elevated ALT**	1	1	0	0
**Elevated AST**	0	0	1	1
**Headache**	3	0	2	0
**Dyspnoea**	0	0	1	1
**Fatigue**	1	0	4	0
**Fever**	4	0	2	1

Supplementary table 6. Overall CTCAE v5 CPI treatment related graded toxicities by treatment arm

**Adverse event**	**Arm A (n=10)**		**Arm B (n=11)**	
	**Any grade**	**Grade 3 or 4**	**Any grade**	**Grade 3 or 4**
**Dry skin**	1	0	0	0
**Maculopapular rash**	2	1	2	0
**Pruritus**	2	0	1	0
**Rash**	2	0	2	0
**Vitiligo**	0	0	1	0
**Hyperthyroidism**	1	0	3	0
**Hypothyroidism**	2	1	1	0
**Abdominal pain**	0	0	1	0
**Diarrhoea**	2	0	3	1
**Nausea**	1	0	0	0
**Vomiting**	1	0	1	0
**Hyperglycaemia**	0	0	1	1
**Hyperkalaemia**	1	0	0	0
**Elevated ALT**	2	2	0	0
**Elevated AST**	2	1	0	0
**Headache**	1	0	0	0
**Arthralgias**	1	0	0	0
**Fatigue**	2	0	2	0
**Fever**	1	0	1	0

Supplementary table 7. Arm B ctDNA change when targeted therapy restarted

ID	Re-started Targeted	VAF – pre restart of TT	Lowest VAF-on TT following re-start	% decrease
4	Yes	4.86	0	100
11	Yes	8.32	0	100
13	Yes	15.73	2.06	87
14	Yes	0.66	0	100
19	Yes	51.58	24.32	53
21	Yes	14.86	0	100

Supplementary table 8. Arm B ctDNA rise following switch to CPI and radiological result

ID	ctDNA rise	Time between ctDNA rise & subsequent scan (days)	Scan result
2*			
4	Yes	63	PD
5**			
7	No		
11	Yes	40	PD
12	No		***
13	Yes	54	PD
14	Yes	22/33	PR/PD[$]
19	Yes	35	PD
20	No		***
21	Yes	7	PD

* patient withdrew before CPI was given and patient was still alive

** patient died before CPI

***PR/CR

[$] First scan 22 days after ctDNA rise showed PR however following scan after 33 days showed PD

CAcTUS

Circul**A**ting **T**umour DNA g**U**ided therapy **S**witch

A parallel arm, biomarker driven, phase II feasibility trial to determinethe role of circulating tumour DNA in guiding a switch between targeted therapy and immune therapy in patients with advanced cutaneous melanoma.

Chief Investigator:	Professor Paul Lorigan
Sponsor:	The Christie NHS Foundation Trust
Funders:	Bristol Myers Squibb
	The Christie Charities

PROTOCOL

Version:	6.0
Date:	05 July 2022
IRAS Number:	227794
Clinicaltrials.gov Number:	NCT03808441
BMS Reference:	BMS CA209-951
Sponsor Reference:	CFTSp129

The undersigned confirm that the following protocol has been agreed and accepted and that the Chief Investigator agrees to conduct the trial in compliance with the approved protocol and will adhere to the principles outlined in the Medicines for Human Use (Clinical Trials) Regulations 2004 (SI 2004/1031), amended regulations (SI 2006/1928) and any subsequent amendments of the clinical trial regulations, GCP guidelines, the Sponsor's (and any other relevant) SOPs, and other regulatory requirements as amended.

I agree to ensure that the confidential information contained in this document will not be used for any other purpose other than the evaluation or conduct of the clinical investigation without the prior written consent of the Sponsor.

I also confirm that I will make the findings of the trial publically available through publication or other dissemination tools without any unnecessary delay and that an honest accurate and transparent account of the trial will be given; and that any discrepancies and serious breaches of GCP from the trial as planned in this protocol will be explained.

For and on behalf of the Trial Sponsor:

Signature: Date:

..../....../......

Name (please print):

....

Position:

....

Chief Investigator:

Signature: Date:

..../....../......

Name: (please print):

....

Statistician(s):

Signature: Date:

..../....../......

Name: (please print):

....

Position: Supervising Statistician

……………………………………..

Signature: Date:

..../....../......

Name: (please print):

....

Position: Trial Statistician

....

SIGNATURE PAGE

Principal Investigator

I, ___________as Principal Investigator for the CAcTUS trial, confirm that I will be responsible to ensure that all members of the local clinical trial team are appropriately trained on the trial protocol and have the relevant qualifications and experience to carry out their role in accordance with the trial protocol.

Name	Site	Signature	Date

I. Key Trial Contacts

Randomisation details	Visit **http://ctu.co.uk**

Chief Investigator	Paul Lorigan	Tel: 0161 446 8002<br>Email: Paul.lorigan@christie.nhs.uk
Co-investigators (translational)	Richard Marais	Tel: 0161 306 6017<br>Email: Richard.marais@manchester.ac.uk
Co-investigators (translational)	Caroline Dive	Tel: 0161 306 6260<br>Email: Caroline.dive@manchester.ac.uk
Co-investigators (clinical research fellow)	Rebecca Lee	Tel: 07764801907<br>Email: beckilee@doctors.org.uk
Sponsor	The Christie NHS Foundation Trust	Tel: 0161 918 2480<br>Email: the-christie.sponsoredresearch@nhs.net
Clinical Trials Unit	Manchester Clinical Trials Unit	Tel: 0161 306 3150<br>Email: cactus@manchester.ac.uk
Trials Pharmacist	Alkesh Patel	Tel: 0161 918 2380<br>Email: Alkesh.patel@nhs.net

II. Peer Review

This protocol has been peer reviewed by 2 independent reviewers.

III. Contents

I. Key Trial Contacts 5
II. Peer Review 5
III. Contents 6
IV. Abbreviations and glossary 10
V. Trial Summary 14
VI. Funding 20
VII. Lay Summary 21
1 Introduction and study rationale 23
1.1 Effect of MAP kinase pathway inhibition on the tumour microenvironment 24
1.2 Use of circulating tumour DNA to define optimal response 26
1.3 Trial rationale 27
2 Research Hypotheses 31
3 Objectives and outcome measures 31
3.1 Trial objectives 31
3.1.1 Primary objectives 31
3.1.2 Secondary objectives 32
3.1.3 Exploratory objectives 32
3.1.4 Translational objectives 33
3.2 Trial endpoints 33
3.2.1 Primary endpoints 33
3.2.2 Secondary clinical endpoints 33
3.2.3 Exploratory efficacy endpoints 34
4 Study design 34
5 Statistics and data analysis 37
5.1 Sample size 37
5.2 Trial efficacy measurement 38
5.2.1 Secondary outcomes 38
5.2.2 Exploratory outcomes 39
5.3 Statistical analysis principles 41
5.4 Summary of baseline data and flow of patients 41
5.5 Primary analysis 42
5.6 Secondary analyses 42
5.7 Subgroup analyses 43
5.8 Adjusted analyses 43
5.9 Other analyses 43
5.9.1 Exploratory 43

5.9.2 *Sensitivity* ... *44*
5.10 Interim analysis and criteria for the premature termination of the trial ... 44
5.11 Patient population ... 45
5.12 Procedures to account for missing or spurious data ... 45

6 Trial ... 46
7 Participant eligibility ... 48
7.1 Inclusion criteria ... 48
7.2 Exclusion criteria ... 49

8 Trial Procedures ... 52
8.1 Patient Identification ... 52
8.2 Informed Consent ... 52
8.3 Screening Procedures ... 53
8.4 Randomisation ... 54
8.4.1 *Sequence generation* ... *54*
8.4.2 *Allocation concealment mechanism and implementation of the allocation sequence.* *54*
8.5 Blinding ... 55
8.6 Treatment discontinuation ... 55
8.7 Trial withdrawal ... 56
8.8 Withdrawing consent from ancillary studies ... 56
8.9 Trial closure ... 56
8.10 Notification of deaths ... 57
8.11 Lost to follow up ... 57

9 Treatment schedules ... 58
9.1 Scheduled Visits ... 58
9.2 Screening visit ... 59
9.3 Baseline visit ... 60
9.4 On-Treatment Procedures ... 61
9.5 Schedule of trial treatments and assessments ... 64

10 Collection, processing and storage of biospecimens ... 73
10.1 Collection of biospecimens ... 73
10.2 Sample collection and handling ... 73
10.3 Mandatory samples ... 73
10.3.1 *Blood* ... *73*
10.3.2 *Tissue* ... *74*
10.4 Optional samples ... 75

10.4.1 Blood 75
10.4.2 Tissue 75
10.4.3 Microbiome sample collection (stool) 75
10.5 Storage and disposal of biospecimens 75
10.6 Sharing of samples with other centres 76

11 Circulating tumour DNA processing, results and clinical decision making 77
11.1 Prioritisation of bloods 77
11.2 DNA extraction and analysis using droplet digital 77
11.3 Reporting of ctDNA result and clinical decision procedure 77

12 Trial Medication 79
12.1 Study drugs and administration 79

13 Management of toxicities, dose delays and modifications 81
13.1 Restrictions 81
13.2 Dabrafenib and trametinib 81
13.2.1 General Guidelines for Clinically Significant Toxicities (D+T) 81
13.3 Nivolumab and Ipilimumab 82
13.3.1 Dose delay criteria 82
13.3.2 Discontinuation Criteria 83
13.4 Drug supply and distribution 85
13.5 Storage and dispensing 85
13.6 Treatment adherance 85
13.7 Concomitant medications 86
13.8 Prohibited Medications 86
13.8.1 Medications specifically prohibited on dabrafenib and trametinib 86
13.8.2 Medications specifically prohibited on nivolumab and ipilimumab 86
13.9 Radiation therapy 86
13.10 Surgical resection following initial response 87
13.11 Permitted therapy 87
13.12 Treatment of Study Drug Overdose 88

14 Safety reporting and pharmacovigilance 89
14.1 Detecting and recording AEs and SAEs 90
14.2 Reporting procedures 90
14.2.1 Adverse Events 90
14.2.2 Serious Adverse Events and Notable Events 91
14.2.3 SAE Processing and Notable Event Reporting at the Manchester CTU92

14.2.4 SUSAR Processing and Reporting at the Manchester CTU 94
14.3 Pregnancy reporting 96
14.4 Overdose reporting 96
14.5 New Safety Findings 97
14.6 Urgent Safety Measures 97
14.7 Periodic Safety Reports 98
14.8 Responsibilities 98
15 Data Handling 100
15.1 Data collection tools and source document identification 100
15.2 CRFs as Source Documents 100
15.3 Data Handling and Record Keeping 100
15.4 Access to Data 101
15.5 Archiving 101
16 Trial Monitoring and Management 102
16.1 Monitoring 102
16.1.1 Remote Monitoring 102
16.1.2 On-Site Monitoring 102
16.1.3 Access to data 103
16.2 Trial committees and management 103
16.3 Public and patient involvement 104
17 Ethical and regulatory considerations 105
17.1 Research Ethics Committee Review 105
17.2 Regulatory compliance 105
17.3 Data protection and patient confidentiality 105
17.4 Protocol compliance 106
17.5 Notification of Serious Breaches to GCP and/or the protocol 107
17.6 Indemnity 107
18 Publication Policy 108
19 References 109
20 Appendices 114
Appendix A. Response criteria (Recist v1.1) 114
Appendix B. ECOG performance status 115
Appendix C. Cockcroft Gault Formula and Wright Formula 116
Appendix D. WOCBP and male participants sexually active with WOCBP 117
Appendix E. Medications specifically prohibited on dabrafenib and trametinib 119
Appendix F. Management of immune related adverse events 122

IV. Abbreviations and glossary

Abbreviation	Definition
AE	Adverse Event
ALT	Alanine Transaminase
ANC	Absolute Neutrophil Count
AR	Adverse Reaction
AST	Aspartate Aminotransferase
BID	Twice daily
BRAFi	BRAF inhibitor
CBC	Cancer Biomarker Centre (formally known as CEP)
cfDNA	Cell free DNA
CI	Chief Investigator
CR	Complete response
CRF	Case Report Form
CRUK	Cancer Research UK
CT	Computerised tomography
CTC	Circulating tumour cells
CTCAE	Common Terminology Criteria for Adverse Events
ctDNA	Circulating tumour DNA
CTM	Clinical Trial Manager
CTU	Clinical Trials Unit
DNA	Deoxyribonucleic acid
ddPCR	Droplet digital PCR
D+T	Dabrafenib +Trametinib

ECG	Electrocardiograph
ECOG	Eastern Cooperative Oncology Group
EMA	European Medicines Agency
EudraCT	European Clinical Trials Database
FACS	Fluorescence-activated cell sorting
FDA	Food and Drug Administration
FFPE	Formalin fixed paraffin embedded
GCP	Good Clinical Practice
HRA	Health Research Authority
IB	Investigator Brochure
INR	International Normalised Ratio
ICF	Informed Consent Form
ICH	International Conference on Harmonisation of technical requirements for registration of pharmaceuticals for human use
IDMC	Independent data monitoring committee
ISF	Investigator site file
ISRCTN	International Standard Randomised Controlled Trials Number
IV	Intravenous
KCL	Kings College London
LDH	Lactate dehydrogenase
LIMS	Laboratory Information Management System
LLN	Lower Limit of Normal
Manchester CTU	Manchester Clinical Trials Unit
MAPK	Mitogen-activated Protein Kinase
MCRC	Manchester Cancer Research Centre
MEKi	MEK inhibitor

MHRA	Medicines and Healthcare products Regulatory Agency
MRI	Magnetic resonance imaging
MTA	Material transfer agreement
N+I	Nivolumab + Ipilimumab
NK	Natural killer
OS	Overall survival
PBMC	Peripheral Blood Mononuclear Cell
PD	Progressive disease
PFS	Progression free survival
PI	Principal Investigator
PIS	Participant Information Sheet
PR	Partial response
PT	Prothrombin Time
PTT	Partial Thromboplastin Time
RCT	Randomised Controlled Trial
REC	Research Ethics Committee
RECIST	Response Evaluation Criteria in Solid Tumours
RNA	Ribonucleic acid
SAE	Serious Adverse Event
SAP	Statistical Analysis plan
SAR	Serious Adverse Reaction
SD	Stable disease
SDV	Source Data Verification
SOP	Standard Operating Procedure
SmPC	Summary of Product Characteristics

SUSAR	Suspected Unexpected Serious Adverse Reaction
TMF	Trial Master File
TMG	Trial Management Group
TSC	Trial Steering Committee
TTP	Time to progression
ULN	Upper Limit of Normal
VAF	Variant allele frequency
WOCBP	Women of child bearing potential

V. Trial Summary

Trial Title	A parallel arm, biomarker driven, phase II feasibility trial to determine the role of circulating tumour DNA in guiding a switch between targeted therapy and immune therapy in patients with advanced cutaneous melanoma
Short title	Circulating tumour DNA guided therapy switch
Primary Objectives	Feasibility:<br>• To estimate the proportion of critical (red) blood samples returned within 7 working days.<br>• To assess whether a decrease in ctDNA levels of mutant *BRAF* by ≥80% on targeted therapy is an appropriate cut off for switching to immune therapy
Secondary Objectives	Feasibility<br>• To assess whether *BRAF* VAF (within the ctDNA) of ≥1.5% is an appropriate target for study inclusion (by assessing the number and proportion of screen failures due to *BRAF* VAF <1.5%)<br>Efficacy<br>• To explore whether PFS at 12 months would improve in patients switching from targeted to immune therapy on response to treatment as guided by ctDNA levels of mutant BRAF VAF<br>• To explore whether survival outcomes would improve in patients switching from targeted to immune therapy on response to treatment as guided by ctDNA levels of mutant BRAF VAF.
Exploratory Objectives	• To define the level of ctDNA level of mutant BRAF VAF rise that determines ctDNA progression and relapse on treatment<br>• To compare time to ctDNA first progression between study arms<br>• To compare time to ctDNA second progression between study arms<br>• To estimate mean change in ctDNA level of mutant BRAF VAF during washout period from targeted to immune therapy switch in arm B

	• To compare duration in ctDNA level of mutant BRAF VAF response to targeted therapy between study arms<br>• To compare duration of mutant BRAF VAF ctDNA response to immune therapy between study arms<br>• To explore the relationship between observing a rise in ctDNA level of mutant *BRAF* VAF and progressive disease observed from scheduled scan results<br>• To explore whether switching from targeted to immune therapy on treatment response as guided by ctDNA levels of mutant BRAF VAF will increase response to therapy.<br>• To assess the time taken for mutant ctDNA levels of BRAF VAF to reach ≥80% decrease on first line immune therapy (Arm A).
Translational Objectives	• Samples will be collected for future work including tumour biopsies and blood. In addition stool samples will be taken for microbiome analyses.
Primary Endpoints and Outcomes	Feasibility<br>• ctDNA result critical (red) blood samples returned within 7 working days of samples being received in the laboratory<br>• Decrease in ctDNA level of mutant BRAF≥80% on targeted therapy
Secondary endpoints and outcomes	Feasibility<br>• Screen failure due to ctDNA levels of mutant BRAF VAF <1.5%<br>Efficacy<br>• First progression free survival (PFS) at 12 months<br>• First progression free survival<br>• Second progression free survival<br>Overall survival (OS)
Exploratory Efficacy Endpoints	• Time to ctDNA first progression<br>• Time to ctDNA second progression<br>• Increase in ctDNA levels of BRAF VAF during washout period from targeted to immune therapy switch in arm B

	• Duration of mutant BRAF VAF (within ctDNA) response to targeted therapy<br>• Duration of mutant BRAF VAF (within ctDNA) response to immune therapy<br>• Time between observing rise in ctDNA levels of mutant *BRAF* VAF and progressive disease observed on scheduled scan<br>• Time taken for mutant ctDNA levels of mutant BRAF VAF to reach ≥80% decrease on targeted therapy<br>• Time taken for mutant ctDNA levels of BRAF VAF to reach ≥80% decrease on first line immune therapy.<br>• ctDNA level of mutant BRAF VAF (at each follow-up assessment time point)<br>• Best overall response rate to immune therapy<br>• Duration of response to immune therapy<br>• Progression-free survival on immune therapy from date of commencement of immune therapy
Trial Design	Parallel arm randomised phase II feasibility trial
Trial Participants	Patients over 18 years old with stage III unresectable/IV cutaneous melanoma with *BRAF* mutation and mutant ctDNA levels of BRAF VAF ≥1.5%
Planned Sample Size	Originally 40 patients but following interim review 21 deemed sufficient
Participating sites	This is a multi-centre study. Some of the sample collection and translational research is applicable only to the Christie NHS Foundation Trust.

Inclusion criteria	1. Patient capable of giving written informed consent<br>2. Patients must be willing and able to comply with scheduled visits, treatment schedule, laboratory tests and other requirements of the study.<br>3. Histological confirmation of cutaneous melanoma<br>4. ≥ 18 years<br>5. Stage III un-resectable/ IV disease<br>6. *BRAF* p.V600E/K/R mutation confirmed (exact point mutation must be provided to the investigators)<br>7. At least one target lesion measurable by CT or MRI as per RECIST 1.1<br>8. Screening ctDNA (as defined by the mutant *BRAF* VAF in plasma) ≥1.5%<br>9. Adequate organ function<br>10. ECOG performance status 0/1/2<br>11. Prior radiotherapy or radiosurgery must have been completed at least 2 weeks prior to the first dose of study drug<br>12. Women of childbearing potential (WOCBP) must have a negative serum or urine pregnancy test (minimum sensitivity 25 IU/L or equivalent units of HCG) within 24 hours prior to the start of study drug.<br>13. WOCBP must agree to follow instructions for method(s) of contraception for the duration of treatment with study drugs plus 5 half-lives of the drugs they are taking at treatment completion (5 times the half-life = 125 days [nivolumab]; 5 times the half-life = 90 days [ipilimumab]; 5 times the half life = 40 hours [dabrafenib]; 5 times the half life = 50 days [trametinib]) plus 30 days (duration of ovulatory cycle).<br>14. Males who are sexually active with WOCBP must agree to follow instructions for method(s) of contraception for the duration of treatment plus 5 half-lives of the study drug as above plus 90 days (duration of sperm turnover).<br>15. Azoospermic males and WOCBP who are continuously not

	heterosexually active are exempt from contraceptive requirements. However, participating WOCBP must still undergo pregnancy testing (see Appendix D)
Exclusion criteria	1. Prior systemic anti-cancer treatment (immune therapy, targeted therapy, vaccine therapy, or investigational treatment) for unresectable Stage III or Stage IV melanoma.<br>2. Prior adjuvant therapy with combination PD-1 inhibitor plus CTLA-4 inhibitor.<br>- Received prior adjuvant therapy with PD-1 inhibitor treatment, which was stopped due to grade 3 or 4 toxicity.<br>- Received prior adjuvant therapy with BRAF/MEK inhibitor **if** relapse occurred < 6 months from discontinuation of treatment or treatment stopped due to grade 3 or 4 toxicity.<br>3. Current use of a prohibited medication<br>4. History of another malignancy. **Exception:** patients who have been disease-free for 3 years, (i.e. patients with second malignancies that are indolent or definitively treated at least 3 years ago) or patients with a history of completely resected non-melanoma skin cancer no additional therapy should be required whilst the patient is on study.<br>5. Any serious or unstable pre-existing medical conditions (aside from malignancy exceptions specified above), psychiatric disorders, or other conditions that could interfere with the patient's safety, obtaining informed consent, or compliance with study procedures.<br>6. Known Human Immunodeficiency Virus (HIV), Hepatitis B Virus (HBV), or Hepatitis C Virus (HCV) infection.<br>7. A history of glucose-6-phosphate dehydrogenase (G6PD) deficiency.<br>8. Patients with active, known or suspected autoimmune disease. Patients with type 1 diabetes mellitus, hypothyroidism only requiring hormone replacement, skin disorders (such as vitiligo,

	psoriasis or alopecia) not requiring systemic treatment, or conditions not expected to recur in the absence of an external trigger will be permitted to enrol.
Exclusion criteria (Cont.)	9. Patients with a condition requiring systemic treatment with either corticosteroids (>10 mg daily prednisone equivalent) or other immunosuppressive medications within 14 days of study drug administration. Inhaled or topical steroids and adrenal replacement steroid doses > 10 mg daily prednisone equivalent are permitted in the absence of active autoimmune disease.<br><br>10. Patients with interstitial lung disease that is symptomatic or may interfere with the detection or management of suspected drug-related pulmonary toxicity.<br>11. Brain metastases and leptomeningeal metastases are excluded unless:<br>o Asymptomatic and untreated at presentation, OR<br>o Symptomatic lesions have been definitively treated with surgery or stereotactic surgery (whole-brain radiation may be given as adjuvant treatment), and do not require steroids for control of symptoms<br>o Symptomatic metastases, treated or untreated, or metastases requiring steroids to control symptoms, are excluded<br><br>12. No enzyme inducing anticonvulsants for ≥ 4 weeks prior to randomisation<br><br>13. Coronary syndromes (including myocardial infarction within 6 months or unstable angina)<br><br>14. A history or evidence of current ≥ Class II congestive heart failure as defined by the New York Heart Association (NYHA) guidelines with an ejection fraction of <50%<br>15. Treatment refractory hypertension defined as a blood pressure of systolic> 150 mmHg and/or diastolic > 95 mm Hg on >3 occasions which cannot be controlled by anti-hypertensive

	therapy;<br>16. Known cardiac metastases;<br>17. Uncorrectable electrolyte abnormalities (e.g. hypokalaemia, hypomagnesaemia, hypocalcaemia), long QT syndrome or taking medicinal products known to prolong the QT interval.<br>18. A history or current evidence/risk of retinal vein occlusion (RVO) or central serous retinopathy (CSR) including presence of predisposing factors to RVO or CSR (e.g., uncontrolled glaucoma or ocular hypertension, uncontrolled hypertension, uncontrolled diabetes mellitus, or a history of hyperviscosity or hypercoagulability syndromes)<br>19. Known immediate or delayed hypersensitivity reaction or idiosyncrasy to drugs chemically related to the study treatments including monoclonal antibodies, their excipients, and/or dimethyl sulfoxide (DMSO) and/or Polysorbate-80-containing infusions.<br>20. Females who are breast-feeding.<br>21. Prisoners or patients who are involuntarily incarcerated.<br>22. Patients who are compulsorily detained for treatment of either a psychiatric or physical (e.g., infectious disease) illness.
Treatment duration	Until second progression.
Follow-up duration	3 months after the last patient has been randomised.
Planned Trial Period	2 years recruitment plus last patient 3 months follow up, plus a further 3 years remote survival data collection

VI. Funding

This trial is funded by Bristol Myers Squibb Pharmaceuticals Limited and The Christie Charities.

VII. Lay Summary

Melanoma is an aggressive type of skin cancer. When it cannot be removed by surgery or has spread to different parts of the body, then there are several treatments currently available, which can help to improve symptoms and may extend life.

There are two main types of treatment, which are commonly used to treat melanoma that has spread;

1. **Immune therapy** (using the immune system to kill cancer cells) and,
2. **Targeted therapy** (affects how cancer is able to grow and survive).

Presently, it is not clear whether we should give immune therapy or targeted therapy first. This trial is investigating whether we should use targeted therapy prior to immune therapy in order to boost the immune response.

In our healthy body we have immune cells, which fight off infections. They work by acting on proteins, which are on the surface of all cells. We can boost their effect with certain immune therapy drugs. The **immune therapy** drugs we plan to use in the trial are Nivolumab (Opdivo®) and Ipilimumab (Yervoy®).

Nivolumab works by blocking a protein called PD-1 on the surface of certain immune cells. Blocking this protein activates the immune cells to find and kill cancer cells. Immune cells also have another protein on their surface called CTLA-4. This protein tells the immune cell when to switch off, which normally helps prevent the immune system from attacking normal cells. Ipilimumab blocks the CTLA-4 protein so that the immune cells stay switched on, and active to attack cancer cells.

The **targeted therapy** drugs we plan to use in the trial are called dabrafenib (Tafinlar®) and trametinib (Mekinist®). In about 5 out of 10 people (about half), the melanoma makes too much of a protein called BRAF. This is due to a change in the *BRAF* gene. The BRAF protein tells the cells to grow and survive. Targeted therapy

only works if the change in the *BRAF* gene is present. The targeted therapy then acts to stop the cells from growing. When the two drugs are combined they are better at stopping the cells from growing.

All these drugs can be very effective, however they do not work for all patients and some melanomas can become resistant. Therefore, we need to find ways to improve their efficacy. One approach is to use some of the properties of targeted therapy to improve the effect of immune therapy. There is some evidence that if we treat patients with targeted therapy and they are responding to treatment, this can potentially make the immune therapy more effective. This is not the case when the cancer has become resistant to treatment. Some patients only respond to targeted therapy for a short time and so we need an accurate and dynamic way of monitoring the response to treatment. Traditionally, therapy response has been evaluated through CT scans, every few months. We have developed a blood test, which gives a picture of how the cancer is responding to treatment and can be taken more frequently. The blood test looks for pieces of DNA that are known to have come from the cancer. If the cancer is responding to treatment, we have observed that the amount of cancer DNA in the blood decreases.

The aim of this feasibility study is to test whether we can use the blood test to inform us whether the melanoma is responding to targeted therapy and therefore to switch to immune therapy in response. We need to make sure we switch treatment at the right level of cancer DNA and the trial will provide us the information to make this decision. Furthermore, we will obtain an initial indication as to how well patients respond to this treatment schedule. However, to test this properly we will need to perform a future trial using the information obtained from this study.

1 Introduction and study rationale

The optimal scheduling of targeted and immune therapies in metastatic melanoma is unknown (1–3). Durable responses have been seen with immune therapy and many clinicians have favoured treatment with immune therapy first-line (4). This is mainly due to the association of immune therapy with a subset of patients who are long-term survivors (5). However, subgroup analysis of the Combi-V study has shown that patients with good prognostic factors, i.e. normal lactate dehydrogenase (LDH), good performance status etc, have a 3-year survival of 63% on dabrafenib plus trametinib (6). Therefore targeted therapy may also be associated with significant duration of response in selected populations (6). Non-randomised evidence has suggested that there may be little difference in median survival between targeted and immune combination therapies, although survival data for combination immune therapy is not yet mature, so this may be revised (Figure 1) (3,7).

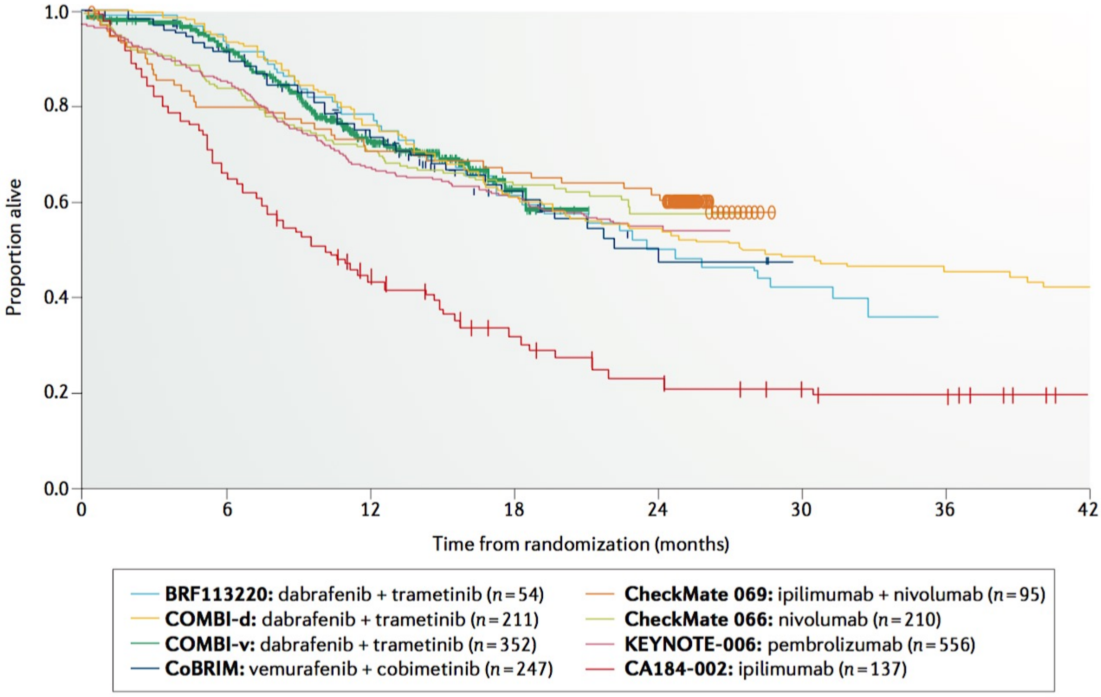

Figure 1 Summary of survival by Kaplan Meir analysis across seminal clinical trials in patients with advanced stage melanoma (3).

Patients with baseline poor prognostic features such as elevated LDH, ECOG performance status >0 and visceral disease continue to have inferior outcomes for both treatment modalities (3). It is clear that further research is required to optimise

the scheduling of these treatments in order to improve survival for patients, especially for those with more aggressive disease.

Studies examining combination targeted and immune therapyhave been largely negative with no significant PFS difference seen for combination of spartalizumab, dabrafenib and trametinib (D+T) vs. D+T in a phase III trial (8). Only a small benefit was seen for PFS as per investigator assessment from 10.6 months (95% CI 9.3–12.7) in the control group (vemurafenib plus cobimetinib) to 15.1 months (11.4–18.4) in the triplet group (atezolizumab, vemurafenib plus cobimetinib; HR 0.78; 95% CI 0.63–0.97; log-rank p=0.025 which was not confirmed by independent assessment (9). Neither trial compared triplet therapy with anti-PD-1 single agent or combination N+I which would be more in line with standard of care in this group of patients. . The results of a study examining dabrafenib and trametinib followed by ipilimumab and nivolumab or ipilimumab and nivolumab followed by dabrafenib and trametinib in patients with stage III-IV *BRAF* V600 melanoma (NCT02224781) are awaited. This trial switches patients on treatment progression rather than at response to either therapy. An alternative approach is to switch patients when they are responding to targeted therapy as this can result in changes to the tumour and the microenvironment that facilitates immune response. The SECOMBIT study and the EBIN studies are both assessing whether a pre-specified period of induction targeted therapy (12 weeks for EBIN and 8 weeks for SECOMBIT) results in improved survival (10,11). Early results from SECOMBIT showed promising results for the induction arm of the trial (10). However, these strategies do not personalise treatment to the individual response of a patient and are at risk (particularly with 12 weeks of therapy) of patients already developing resistant clones which are more immune suppressive due to the length of the induction period.

1.1 Effect of MAP kinase pathway inhibition on the tumour microenvironment

Pre-clinical data has revealed that BRAF inhibition results in an environment that can enhance immune responses (12,13). Tumours responding to BRAF inhibitors (BRAFi) have been shown to have increased T cell infiltration, improved T cell recognition of melanoma associated antigens and reduced production of immunosuppressive

cytokines (3,13–15). Micropthalmia-associated transcription factor (MITF) is up-regulated following BRAF inhibition, resulting in increases in the expression of lineage antigens, such as gp100, melan-A, and tyrosinase-related proteins 1 and 2 (3,16). PD-L1 expression has also been shown to increase following 10-14 days of BRAFi therapy, which could enhance the effect of PD-1 inhibitors (16,17).

In addition, a mouse model examining the anti-tumour effect of dabrafenib plus anti-PD-1 with or without trametinib, showed improved responses with the triplet (13,18). Although MEK inhibitors (MEKi) have been shown to decrease dendritic cell function and T cell activity (13,19), in the context of treatment with BRAFi they may have an immune enhancing function. This could be due to the prevention of BRAFi induced paradoxical activation of tumour associated macrophages, which impair effector T cell entry into tumours and drive melanoma cell growth (20). Further investigation as to the mechanisms behind targeted and immune therapy synergy is required, which will form part of the translational work involved in this trial.

There is evidence that this immune-promoting picture is lost as the melanoma becomes resistant to targeted therapy. Hugo *et al* analysed tumours from patients who had developed resistance to BRAF inhibition and showed that there were decreased numbers of $CD8^+$ T cells, they had impaired function and had lost the ability to recognise antigen (21). Tumours that are resistant to MAP kinase treatment have decreased expression of MITF and associated suppression of melanocyte-lineage antigen expression(3).

Although further research is required to understand the effects of BRAF/MEK inhibition on tumour-immune interactions, this initial data suggests that the optimal time for scheduling of immune therapy post targeted therapy is while the tumour remains sensitive to treatment, rather than when the tumour has become resistant. Furthermore, immune therapy has been shown to have better responses in patients with a lower tumour burden and in whom LDH is normal (22–25). A review by leading experts proposed that high LDH was a key aspect of resistance to immune therapy as it may impair T cell function (26). In mouse models it has been shown that neutralising tumour acidity improves response to immune therapy (27). Lactate dehydrogenase A (LDHA) associated lactate acid production has been shown to impair activation of

natural killer (NK) and T cells (28). Therefore, reducing LDH through targeted therapy before treating with immune therapy could improve responses.

In addition, there is increasing evidence that intermittent schedules or “drug holidays” may delay the onset of resistance to targeted therapy, therefore stopping targeted therapy in response and restarting if there is progression on immune therapy may also prolong response to targeted therapy (29,30). Taken together, these studies suggest that inducing a response with targeted therapy, and then switching to immune therapy when the tumour is still responding could be a promising approach.

1.2 Use of circulating tumour DNA to define optimal response

A precise definition of response is required in order to decide upon a switch to immune therapy. A radiological definition of response is currently the standard assessment. However a scan at a fixed time point of 2 or 3 months does not reflect the wide range of response dynamics. The COWBOY trial (NCT02968303) is examining 6 weeks of induction therapy with vemurafenib and cobimetinib prior to switching to nivolumab plus ipilimumab. However, we feel that more personalised approaches may benefit patients in deciding when to switch treatment. Our data suggests the window of response may be shorter than 6 weeks in some patients with aggressive disease and therefore tailoring treatment to the individual’s response is required (Figure 2).

We have developed techniques using circulating tumour DNA (ctDNA) in the metastatic setting, which are able to accurately monitor tumour burden over time (31). By following tumour-specific mutations in the blood, we were able to show responses to treatment and detect disease progression at an early stage (31). Other groups have also shown that ctDNA can be used to predict outcome to treatment and follow tumour load over time (32,33). Furthermore, the fraction of mutant ctDNA correlates with LDH; patients with high LDH have higher mutant ctDNA fractions compared to those patients with normal LDH (32). We plan to use droplet digital PCR (ddPCR), which is an extremely sensitive assay reaching detection sensitivities of approximately 0.01% (34). In addition, it is relatively simple and quick to perform, with an expected time to result of 7 working days from blood being received by the laboratory. Application of ddPCR to quantify BRAF mutant ctDNA levels enables 2 - 4 weekly patient monitoring

of tumour burden to be efficiently performed, in order to achieve an accurate and dynamic view of treatment response.

1.3 Trial rationale

We propose a trial where patients will be randomised between a standard of care arm (arm A) vs. a switch from targeted to immune therapy based on response in ctDNA. The investigational arm (arm B) will treat patients with targeted therapy until response as defined by ctDNA levels of mutant BRAF VAF and switching to immune therapy (See Section 8 trial schema). If patients progress on immune therapy in arm B they will switch back to D+T as resistance will not have developed to the treatment. In Arm A, investigators, in discussion with the patient, will choose between commencing first line immune therapy with combination nivolumab plus ipilimumab followed by second line targeted therapy (D+T) upon disease progression. Alternatively investigators, in discussion with the patient, can choose first line targeted therapy (D+T) followed by second line immune therapy (N+I) upon disease progression. This is to enable the standard of care arm to mirror current practice in managing advanced BRAF mutant melanoma whereby a choice is made between targeted and immune therapy based on the clinical picture and patient preference.

In order to perform such a study, we first need a feasibility study to optimise a cut off for the mutant *BRAF* variant allele frequency (VAF) in plasma at the time of treatment switch. We propose an initial phase II feasibility trial, which will enable us to test whether a decrease in mutant BRAF VAF level of ≥80% is an appropriate target. The figure of ≥80% is based on our prospective longitudinal data using ddPCR (see figure 2. and table 1. Gremel *et al* unpublished), which shows that the majority of patients (including patients with very aggressive disease) treated first-line with a BRAF inhibitor alone will achieve at least an 80% reduction of *BRAF* VAF early (within 1 month) of treatment. This is likely to be enhanced in patients treated with D+T as deeper responses have been achieved compared to BRAF inhibition alone (35).

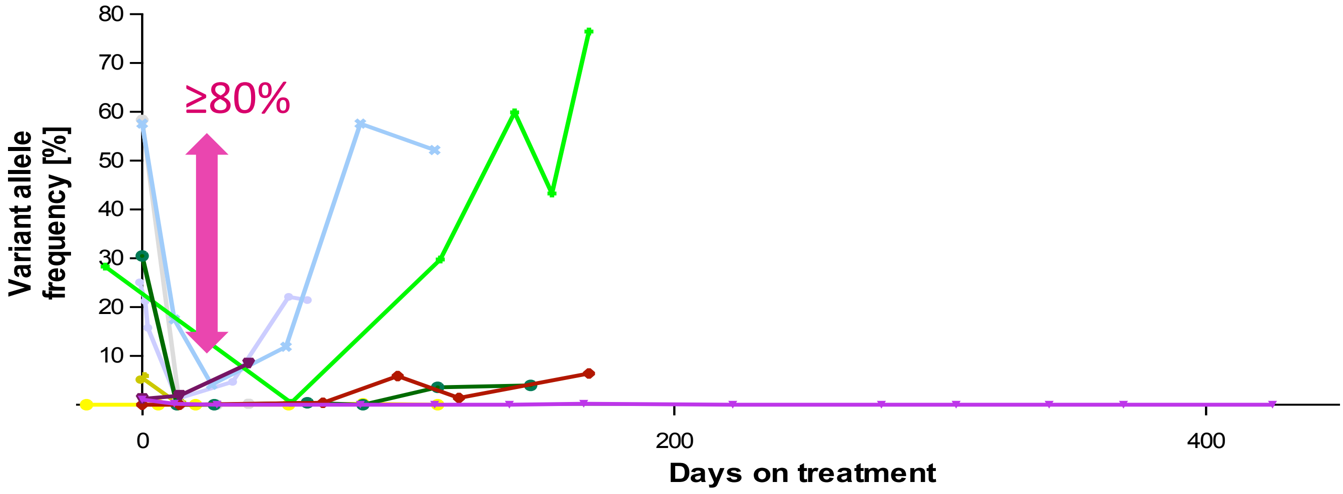

Figure 2 Longitudinal prospective data of mutant BRAF VAF ctDNA on first-line dabrafenib.

Each coloured line represents an individual patient treated with the BRAF inhibitor (Gremel *et al* unpublished). CtDNA levels rapidly decrease on targeted therapy with all patients achieving an 80% drop in ctDNA levels. VAF levels can begin to increase prior to day 90 (3 month standard radiological assessment)

In order to determine whether the 80% threshold is appropriate, we will also follow the ctDNA levels in the standard arm to examine the relative (%) decrease in ctDNA levels of *BRAF* VAF achieved for patients undergoing treatment with dabrafenib plus trametinib. Patients on Arm B will switch treatment within 10 days of ctDNA decrease by 80%. For those patients on Arm B that do not achieve an 80% decrease in BRAF VAF, they will remain on targeted therapy for as long as they are getting clinical benefit, and switch to immunotherapy.

In addition to optimising the VAF cut off for optimal response, the proposed study has logistical targets in terms of reporting of ctDNA results in a timely manner to allow treatment decisions to be made. This feasibility study will provide the opportunity to establish a stringent pipeline, which will be used for the larger Phase III trial.

We will also try to obtain a signal of efficacy through examining PFS at 12 months. A landmark analysis has been chosen, as it will provide an early readout for an efficacy

Table 1 Changes in variant allele frequency (VAF)

Patient	Baseline VAF	Lowest VAF achieved on targeted therapy	Maximum VAF decrease (%)	Time to maximal VAF decrease (days)
1	30.5	0	100	13
2	57.6	4.11	92.9	26
3	25.1	1.2	95.2	13
4	58.7	0	100	14
5	5.5	0	100	14
6	13.4	0	100	14
7	14.7	0.3	98	37

signal. The median PFS of patients receiving D+T was 9.3 and 11.4 months respectively in the COMBI-D and COMBI-V studies (6,35). The population in this trial would be expected to have a shorter PFS, as raised ctDNA is associated with poorer prognosis and therefore the majority of patients in Arm A will have progressed on treatment by 12 months.Therefore, if patients on Arm B do not progress by 12 months, this would be seen as a signal of clinical efficacy. Once the initial study has been conducted and the results analysed, we plan to have a follow-on study utilising the optimised VAF cut-off.

Rationale for change in VAF inclusion criteria level

When we initially designed the study, we based the VAF inclusion criteria on a paper from Santiago-Walker *et al,* which suggested that approximately 30% of patients with a *BRAF* V600E mutation would have a VAF of ≥5% at start of treatment and these would have a shorter progression free survival in response to D+T (36). We expected that a third of patients with *BRAF* mutant melanoma would be eligible for this study using a VAF inclusion criteria cut off of ≥5%. Patients were selected on clinical features of poorer prognosis (such as multi-site disease, visceral disease and high LDH) as these were expected to benefit the most from the treatment. However, based on results from the first ten patients screened, we found that some of the patients who had these features were being screened out due to a VAF <5%, therefore we were missing an important group of patients who might benefit from the CAcTUS strategy.

In addition, review of the lower limit of detection and the lower limit of quantification of the assay for the patients screened for the study revealed that reducing the inclusion VAF to 1.5% would enable more patients to be eligible without compromising the assay performance. Thus, the inclusion criteria has been amended to reflect these new data.

2 Research Hypotheses

1. In *BRAF* mutant melanoma the efficacy of immune therapy is enhanced by response to pre-treatment with MAPK pathway inhibition (D+T).
2. Changes in ctDNA levels can be used to accurately inform when to switch from targeted to immune therapy.

3 Objectives and outcome measures

3.1 Trial objectives

3.1.1 Primary objectives

The primary objectives relate to feasibility and are:

- To estimate the proportion of critical (red) blood samples (see Section 11.1) returned within 7 working days of samples being received in the laboratory. (Aim for 95% of critical (red) samples for a result to be returned ≤7 working days of sample being received by the laboratory.)

- To assess whether a decrease in ctDNA levels of *BRAF* VAF by ≥80% on targeted therapy is an appropriate cut off for switching to immune therapy. (Success will be demonstrated if ≥80% of the patients on targeted therapy achieve ≥80% in ctDNA levels of *BRAF* VAF decrease across the two trial arms.)

The criteria to move to a follow-on study are critical, as real-time decision-making based on ctDNA levels has never been performed and therefore we need to ensure that the ctDNA result is returned in a timely manner in order to make clinical decisions quickly. In addition, we need to ensure that the ≥80% decrease in *BRAF* VAF seen in our pre-clinical findings is a clinically relevant definition of response to targeted therapy in melanoma. Thus, for a follow on study to be progressed without further discussion we would expect that:

- ≥95% of critical (RED) sample results are returned by the laboratory within 7 working days of receipt (although we will consider, whether a trial is feasible if

a substantial majority e.g. ≥95% of results are returned within 10 working days of receipt into the laboratory) and a substantial majority of patients (≥80%) will have an ≥80% *BRAF* VAF decrease on targeted therapy.

3.1.2 Secondary objectives

Feasibility

- Assess whether *BRAF* VAF (within the ctDNA) of ≥1.5% is an appropriate target for study inclusion (by assessing the number and proportion of screen failures due to *BRAF* VAF (within the ctDNA) <1.5%)

Efficacy

- To explore whether Progression-Free Survival (PFS) at 12 months would improve in patients with unresected advanced cutaneous melanoma, switching from targeted to immune therapy on response to treatment as guided by ctDNA levels of mutant BRAF VAF.

- To explore whether survival outcomes would improve in patients switching from targeted to immune therapy on response to treatment as guided by ctDNA levels of mutant BRAF VAF.

3.1.3 Exploratory objectives

- To define the level of ctDNA BRAF VAF rise that determines ctDNA progression and relapse on treatment
- To compare time to ctDNA first progression between study arms
- To compare time to ctDNA second progression between study arms
- To estimate mean change in ctDNA levels of BRAF VAF during washout period from targeted to immune therapy switch in arm B
- To compare duration of mutant BRAF VAF (within ctDNA) response to targeted therapy between study arms

- To compare duration of mutant BRAF VAF (ctDNA) response to immune therapy between study arms
- To explore the relationship between observing a rise in ctDNA levels of mutant *BRAF* VAF and progressive disease observed scheduled scan results
- To explore whether switching from targeted to immune therapy on treatment response as guided by ctDNA levels of mutant BRAF VAF will increase response to therapy.
- To assess the time taken for mutant ctDNA levels of BRAF VAF to reach ≥80% decrease on first line immune therapy (Arm A).

3.1.4 Translational objectives

- Samples will be collected for future work including tumour biopsies and blood. In addition stool samples will be taken for microbiome analyses.
  Due to the hypothesis-generating design of translational studies, these will not be performed to GCP standard, however will be conducted with scientific rigour in accordance with principles of respect for persons, beneficence and justice.

3.2 Trial endpoints

3.2.1 Primary endpoints

Feasibility

- CtDNA result critical (red) blood sample returned within 7 working days of samples being received in the laboratory
- Decrease in ctDNA level of mutant BRAF ≥80% on targeted therapy

3.2.2 Secondary clinical endpoints

Feasibility

- Screen failure due to ctDNA levels of mutant *BRAF* VAF <1.5%

Efficacy

- First progression-free survival at 12 months
- First progression-free survival
- Second progression-free survival
- Overall survival

3.2.3 Exploratory efficacy endpoints

- Time to ctDNA first progression
- Time to ctDNA second progression
- Increase in ctDNA levels of BRAF VAF during washout period from targeted to immune therapy switch in arm B
- Duration of mutant BRAF VAF (within ctDNA) response to targeted therapy
- Duration of mutant BRAF VAF (within ctDNA) response to immune therapy
- Time between observing rise in ctDNA levels of mutant *BRAF* VAF and progressive disease observed on scheduled scan
- Time taken for mutant ctDNA levels of mutant BRAF VAF to reach ≥80% decrease on targeted therapy
- Time taken for mutant ctDNA levels of BRAF VAF to reach ≥80% decrease on first line immune therapy.
- ctDNA level of mutant BRAF VAF (at each follow-up assessment time point)
- Best overall response rate to immune therapy
- Duration of response to immune therapy
- Progression-free survival on immune therapy from date of commencement of immune therapy

4 Study design

Protocol waivers or exemptions are not allowed with the exception of Urgent Safety Measures, please see Section 14.6 for further details. Therefore, adherence to the study design requirements including those specified in the schedule of visits, are essential and required for study conduct.

This is a feasibility study to determine whether a decrease in BRAF VAF (within ctDNA) of ≥80% is an appropriate cut-off for a decision to switch from targeted to immune therapy. In addition it will determine whether ctDNA levels of BRAF VAF≥1.5% is an appropriate target for study inclusion. We expect 30-40% of patients with *BRAF* mutant melanoma would be eligible for this study using a VAF inclusion criteria cut off of ≥1.5%. A follow-on trial will be performed, provided that feasibility criteria are met (see Section 3.1.1) and that a signal of efficacy is seen.

Patients with histologically confirmed cutaneous melanoma that is either unresectable Stage III or Stage IV disease will be screened for eligibility (see inclusion/exclusion criteria). Eligible patients must be BRAF p.V600E/K/R mutation positive (determined by a CPP accredited laboratory) and the exact point mutation must be identified in order to perform the correct analysis. Patients who have had prior systemic MAPK targeted therapy or immune therapy will not be eligible. Blood will be taken at the screening visit in order to determine the BRAF VAF in plasma ctDNA.

In patients where there is rapid disease progression or the patient does not wish to delay start of D+T until the ctDNA result is confirmed, then immediate commencement of D+T is permitted on the day of screening. The ctDNA result and patient randomisation must occur prior to the next scheduled visit (2 weeks after start of D+T) for the patient to be eligible for the study (hence the preference for results to be returned within 7 days, but potential feasibility if this is within 10 days, allowing 3 days for the sample to arrive in the lab). If the *BRAF* ctDNA VAF is ≥1.5% then the patient will be randomised into the study, however if *BRAF* ctDNA VAF is <1.5% they will not enter the study.

For patients who choose to start immune therapy first it is recommended (but not mandatory) to book a chair and inform the laboratory/MCTU of the intended date of treatment. The patient should be informed that this may be postponed if they randomise to Arm B. In order to have immune therapy first on study the patient must have received the result of their *BRAF* ctDNA VAF (screen in ≥1.5%) and have been

randomised to Arm A. Patients with lower disease burden and no clinical need or preference to start treatment straight away can also wait for the ctDNA result (7 days + time for the blood to arrive in the laboratory: estimated at maximum total 10 days). If the *BRAF* ctDNA VAF is ≥1.5% then the patient will be consented to the study and randomised, however if ctDNA VAF is <1.5% they will not enter on to the study.

Patients will be randomised to either Arm A (Standard arm) or Arm B (ctDNA guided switch). Treatment will be as follows:

- **ARM A Standard arm**: Investigator choice between dabrafenib plus trametinib or nivolumab plus ipilimumab first line then switching on progression to the other treatment second line.

Patients commencing dabrafenib plus trametinib first line

Dabrafenib 150mg twice daily with trametinib 2mg once daily. On disease progression or unacceptable toxicity, washout of 7 days for trametinib and dabrafenib to stop the day before commencement of nivolumab in combination with ipilimumab. Then switch to nivolumab 1 mg/kg administered as an intravenous infusion over 60 minutes every 3 weeks for the first 4 doses in combination with 3 mg/kg ipilimumab administered intravenously over 90 minutes. Then followed by a second phase in which 480mg flat dose of nivolumab is administered as an intravenous infusion over 60 minutes every 4 weeks until disease progression or unacceptable toxicity.

Patients commencing nivolumab plus ipilimumab first line

Nivolumab 1 mg/kg administered as an intravenous infusion over 60 minutes every 3 weeks for the first 4 doses in combination with 3 mg/kg ipilimumab administered intravenously over 90 minutes. Then followed by a second phase in which 480mg flat dose of nivolumab is administered as an intravenous infusion over 60 minutes every 4 weeks. On disease progression or unacceptable toxicity, washout of 7 days then commence dabrafenib 150mg twice daily with trametinib 2mg once daily until disease progression or unacceptable toxicity.

- **ARM B ctDNA guided switch:** Dabrafenib 150mg twice daily with trametinib 2mg once daily. Following decrease of ctDNA levels of mutant *BRAF* VAF by 80% or more, trametinib will be stopped for 7 days with dabrafenib continuing until the day before switching treatment. A CT scan/ dual phase MRI scan will be performed in order to correlate radiological imaging with ctDNA response (additional blood test taken on day of imaging). The patient will then commence nivolumab 1 mg/kg administered as an intravenous infusion over 60 minutes every 3 weeks for the first 4 doses in combination with 3 mg/kg ipilimumab administered intravenously over 90 minutes. Then followed by a second phase in which 480mg flat dose of nivolumab is administered as an intravenous infusion over 60 minutes every 4 weeks. If there is disease progression as per RECIST 1.1, then switch back to dabrafenib 150mg twice daily with trametinib 2mg once daily. Investigator assessed clinical progression must be discussed with the Chief Investigator. If unacceptable toxicity then re-assess disease and if disease progression after at least 4 weeks following start of N+I treatment, switch back to dabrafenib plus trametininb.

5 Statistics and data analysis

5.1 Sample size

The original study sample size target was 40 patients (20 in each arm) which would suffice for the main research questions about feasibility to be answered based on true feasibility sample size parameters of a:

i. 95% (or greater) return rate of critical (red) blood samples;

ii. 80% (or greater) success rate of participants on targeted therapy achieving ≥80% *BRAF* VAF decrease in ctDNA levels

enabling estimation of:

i. the return rate with a two-sided 95% confidence interval of width no more than 12% points (i.e. 87% to 99%) assuming at least 60 critical red blood samples (one sample from each of the 20 Arm A participants and an average of at least two samples from each of the 20 Arm B participants) –see Section 10.1.

ii. the success rate of participants on targeted therapy achieving ≥80% *BRAF* VAF decrease in ctDNA levels with a two-sided 95% confidence interval of width equal to 30% points (i.e., 62%, 92%), assuming that 25% of the participants in arm A will receive D+T as first line treatment (i.e., 25 participants across arms; 5 in Arm A and 20 in Arm B)

On interim review of the return rate, there was good evidence of a high return rate and hence a 95% confidence interval of similar width (14%) could be obtained from a sample of 21 or more participants (resulting in at least 32 critical red blood samples, assuming one sample from Arm A participants and at least two samples from Arm B participants). Moreover, with a 'red' sample size of (at least 32) samples provides 80% confidence that the lower confidence limit is at least 90% if the *true* return rate is at least 98% (i.e. we can be 80% confident that at least 95% of the 'red' samples will be returned in a timely manner. Likewise, with a sample size of 17 participants with ctDNA reduction data, we can be 80% confident that at least 80% of participants will achieve a reduction in ctDNA levels of at least 80% from baseline (i.e. at least 80% 'success rate'), assuming that the true 'success rate' percentage is 95% (or greater). There will therefore be a reasonable degree of scientific evidence for the feasibility objectives based on a sample size of 21 participants (10 Arm A, of whom 6 receive D+T, and 11 Arm B, all of whom receive D+T and provide at least two 'red' blood samples).
It was therefore deemed appropriate to revise the target sample size from 40 to 21 randomised participants.

5.2 Trial efficacy measurement

5.2.1 Secondary outcomes

i. Progression free survival (PFS) at 52 (+2) weeks, defined as the time interval from randomisation until confirmed first disease progression according to RECIST v1.1 or death, whichever occurs first. Survival times will be censored at the 52 (+2) weeks trial visit if patients have not progressed, and at the observed time known to be progression-free if participants have a follow up period of less than 52 (+2) weeks, are withdrawn or are lost to follow up.

ii. First Progression Free Survival (PFS), defined as the time interval from randomisation until confirmed first disease progression according to RECIST v1.1 or death, whichever occurs first.

iii. Second Progression Free Survival (PFS) defined as the time interval from randomisation until confirmed second disease progression according to RECIST v1.1 or death, whichever occurs first.

iv. Overall survival (OS), defined as the time interval from randomisation until the documented date of death due any cause.

Outcomes ii to iv will be censored at the observed date known the patient "to be alive" or "progression free" if the event of interest is not observed at the end of the follow up period, or patients are withdrawn or lost to follow up.

5.2.2 Exploratory outcomes

i. Time to ctDNA first progression, defined as the time interval from the date of commencement on targeted therapy until the date of confirmed first ctDNA progression or death, whichever occurs first.

ii. Time to ctDNA second progression, defined as the time interval from the date of commencement on targeted therapy until the date of confirmed second ctDNA progression or death, whichever occurs first.

iii. Duration of mutant BRAF VAF (within ctDNA) response on targeted therapy, defined as the time interval from the first recorded date of observing a decrease in mutant BRAF VAF from the date of commencement on targeted therapy until the first recorded date of observing a rise in mutant BRAF VAF, while the patient is on targeted therapy or within 30 days of termination of targeted therapy due to any cause.

iv. Duration of mutant BRAF VAF (within ctDNA) response on immune therapy, defined as the time interval from the first recorded date of observing a decrease in

mutant BRAF VAF until the first recorded date of observing a rise in mutant BRAF VAF, while the patient is on immune therapy or within 30 days of termination of immune therapy due to any cause.

v. Time taken for mutant ctDNA levels of mutant BRAF VAF to reach ≥80% decrease on targeted therapy, defined as the interval time from the date of commencement on targeted therapy until the date in which patients achieve a reduction of at least 80% in ctDNA levels of mutant BRAF VAF or death, whichever occurs first.

vi. Time taken for mutant ctDNA levels of mutant BRAF VAF to reach ≥80% decrease on first line immune therapy, defined as the interval time from the date of commencement on immune therapy until the date in which patients achieve a reduction of at least 80% in ctDNA levels of mutant BRAF VAF or death, whichever occurs first.

vii. Time between observing rise in ctDNA levels of mutant *BRAF* VAF and progressive disease observed on scheduled scan or death, whichever occurs first.

viii. Best overall response (BOR) to immune therapy, defined as the best confirmed response according to RECIST v1.1 that is achieved during immune therapy or within 30 days of any termination of immune therapy due to any cause (e.g., toxicity). Complete response (CR) and partial response (PR) determinations will be included in the BOR assessment.

ix. Duration of response (DoR) to immune therapy, defined as the time interval from initial response (CR or PR whichever status occurs first) according to RECIST v1.1 that is recorded during immune therapy or within 30 days of any termination of immune therapy due to any cause, until the date of confirmed disease progression.

x. Progression free survival (PFS) on immune therapy, defined as the time interval from the date of commencement on immune therapy until the date of confirmed disease progression according to RECIST v1.1 or death, whichever occurs first.

Survival times will be censored at the observed date known the patient “to be alive”, “responding to therapy” or “progression free” if the event of interest is not observed at the end of the follow up period, or patients are withdrawn or lost to follow up.

5.3 Statistical analysis principles

The results of the analyses will be reported following the principle of the ICH E3 guidelines on the Structure and Content of Clinical Study Reports and will also adhere to the CONSORT 2010 guidelines and the CONSORT 2010 extention for reporting pilot and feasibility studies.

As this is a phase II feasibility study, the emphasis will be on producing relevant data summaries with very little role for hypothesis testing. Where appropriate, statistical tests will use a 2-sided p value of 0.05. All confidence intervals presented will be 95% and 2-sided unless otherwise specified.

A detailed Statistical Analysis Plan (SAP) will be developed for approval by the TSC and finalised before the first analysis of unblinded data commences or database lock, whichever occurs first.

All statistical analyses will be performed using Stata (StataCorp, College Station TX, USA).

5.4 Summary of baseline data and flow of patients

A CONSORT diagram (Schulz, et al., 2010) will be used to describe the course of patients through the trial from initial screening for eligibility to completion of the primary outcome assessment.

Demographic and other baseline characteristics will be summarised by randomised groups. Summary measures for the baseline characteristics of each group will be presented as mean and standard deviations for continuous (approximately) normally distributed variables (after log transformation if necessary), median and interquartile ranges for non-normally distributed variables, and frequencies and percentages for categorical variables.

5.5 Primary analysis

The study main feasibility outcomes are:

- ctDNA result critical (red) blood sample returned within 7 working days of samples being received in the laboratory.

- Decrease in ctDNA levels of mutant BRAF ≥80% on targeted therapy.

We will summarise the number (%) of critical (red) blood samples returned by the number of working days since the samples are received in the laboratory. Success will be demonstrated if at least 95% of the critical (red) samples are returned within 7 working days of the samples being received by the laboratory. A 95% confidence interval for the 'success' rate will be presented. Additionally, we will plot a histogram showing the distribution of working days taken for the samples to be returned.

ctDNA levels of BRAF VAF will be summarised (as mean and standard deviations or median and interquartile ranges depending on the distribution of the data) by the number of days patients are on targeted therapy treatment.

We will also show graphically the median ctDNA levels by targeted therapy treatment day with superimposed bars showing the IQR for all patients across treatment days.

Success will be demonstrated if ≥80% of the patients on first line targeted therapy achieve ≥80% BRAF VAF decrease across the two arms. We will present a 95% confidence interval for the 'success' rate.

5.6 Secondary analyses

Feasibility

We will summarise the number (%) of screen failures due to mutant BRAF VAF <1.5% and present a 95% confidence interval for the 'failure' rate. A boxplot will be produced to graphically illustrate the results.

In addition, we will summarise the number (%) of screen failures due to mutant BRAF VAF levels ranging from 1.5% to 5%, in increments of 0.5%, along with their 95% confidence intervals to further assess different cut-off points for study inclusion. Boxplots will also be produced plot the results.

Efficacy

For each of the secondary outcomes; a) PFS at 52(+2) weeks, b) PFS, c) second PFS and d) OS we will estimate the survival curves, median survival times and survival probabilities with their 95% CI for each treatment group using the Kaplan-Meier method.

Differences between treatment arms in survival curves will be compared using a log-rank test.

5.7 Subgroup analyses

There are no pre-specified sub-group analyses planned

5.8 Adjusted analyses

Analysis of survival outcomes will be unadjusted. The robustness of the secondary efficacy analyses will be assessed by stratified analyses (see Section 5.9.2)

5.9 Other analyses

5.9.1 Exploratory

Best overall response (BOR) rate to immune therapy will be estimated by the proportion of patients with a complete or partial response. Treatment effects will be estimated by the difference in response rates between treatment arms and 95% CIs for the differences.

We will produce individual profile plots for each participant showing the ctDNA levels of BRAF VAF against the follow-up assessment time points (both absolute and relative profiles, with ‘relative’ plots being presented as % of screening value) to visually

assess changes in ctDNA levels of BRAF VAF over time and the time taken for the changes to occur.

Exploratory survival outcomes will be analysed using the same approach adopted for the analysis of secondary efficacy outcomes.

The analysis of the remaining exploratory outcomes, will focus on producing relevant data summaries in addition to graphical methods. Full detail of the exploratory analyses will be provided in the SAP.

5.9.2 Sensitivity

We will repeat the analyses of the secondary efficacy outcomes using a stratified log-rank test. This approach will allow comparison of the survival curves between treatment arms taking into account baseline prognostic factors (i.e., LDH, disease site, ctDNA VAF and M stage). Data will be stratified using one variable at a time. This will provide a sensitivity analysis to assess the robustness of the conclusions from the main approach.

Indication for other sensitivity analyses may come to light during the trial conduct, these will be included in the SAP prior to sign off.

5.10 Interim analysis and criteria for the premature termination of the trial

There are no formal planned interim analyses. The Trial Steering Committee (TSC) will take the role of the Independent Data Monitoring Committee (IDMC) who will review periodic reports concerning trial progress and participant safety on a regular basis and may recommend early closure of the study accordingly. This will be based on their expert opinion, there are no formal statistical criteria.

5.11 Patient population

The analysis population will include all randomised participants for whom outcomes are available regardless of their adherence to treatment. Patients will be retained and analysed in their allocated group.

5.12 Procedures to account for missing or spurious data

Survival outcomes will be censored at the observed date known the patient “to be alive” or “progression free” if the event of interest is not observed at the end of the follow up period, or patients are withdrawn or lost to follow up.

Missing values will be summarised as frequencies and percentages for each outcome measure and reported by treatment arm. Reasons for missingness may be important and these will be tabulated by treatment arm and documented as far as possible.

6 Trial

Figure 3 Trial Schema

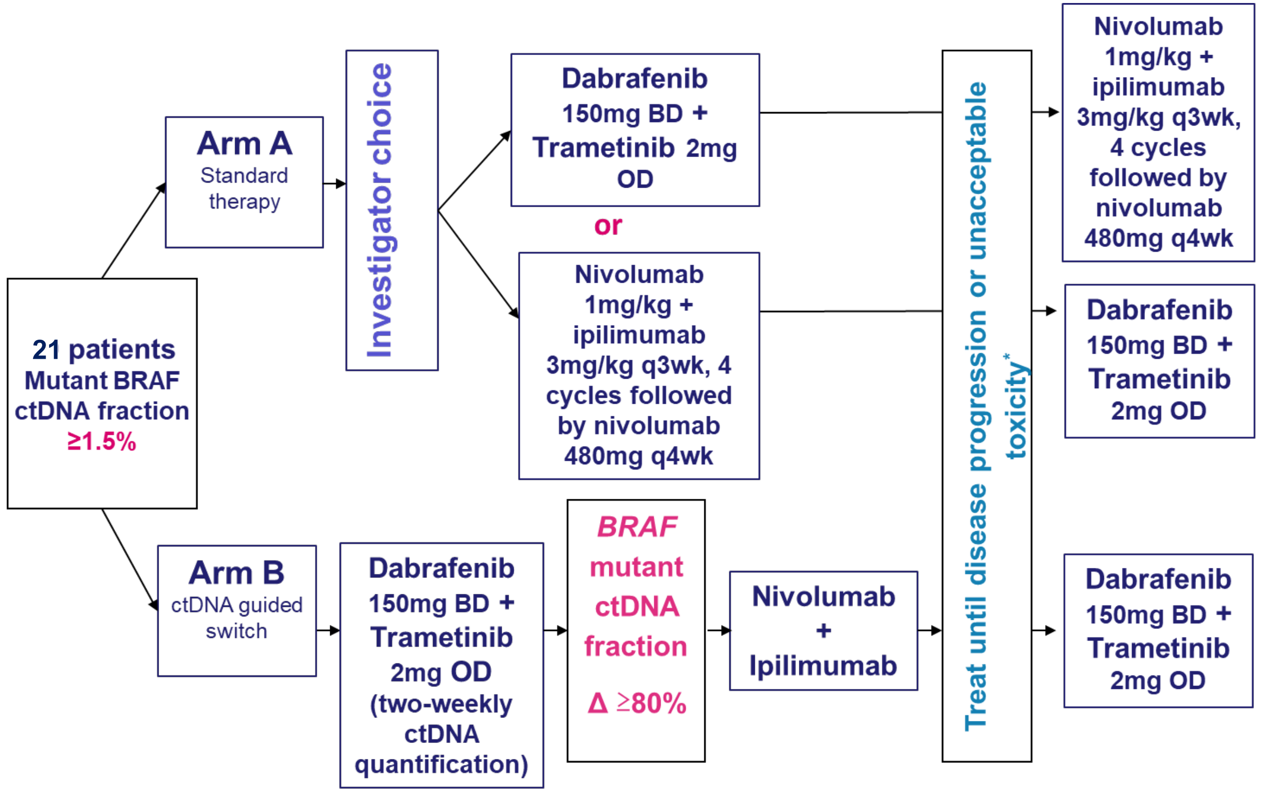

*If toxicity in the immunotherapy arm, reassess response and switch to dabrafenib + trametinib only if confirmed disease progression

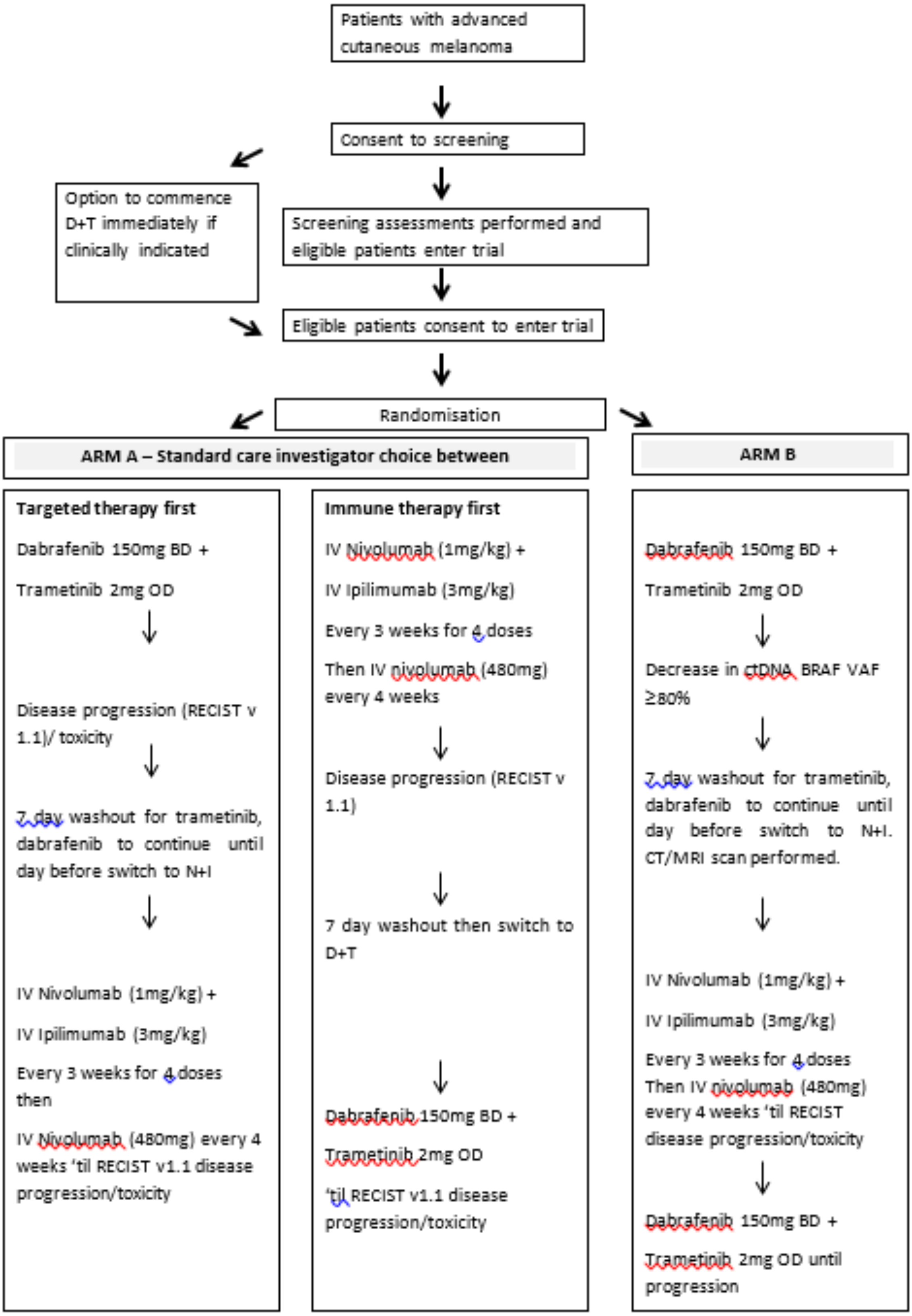

Figure 4 Patient Pathway

7 Participant eligibility

7.1 Inclusion criteria

1. Signed written informed consent
2. Patients must be willing and able to comply with scheduled visits, treatment schedule, laboratory tests and other requirements of the study.
3. Histological confirmation of cutaneous melanoma
4. ≥ 18 years
5. Stage III un-resectable/ IV disease
6. *BRAF* p.V600E/K/R mutation confirmed (exact point mutation must be provided to the investigators)
7. At least one target lesion measurable by CT or MRI as per RECIST 1.1 (see Appendix A)
8. Screening ctDNA (as defined by the mutant *BRAF* VAF in plasma) ≥1.5%
9. Adequate organ function (see table 2.)
10. ECOG performance status 0/1/2 (See Appendix B)
11. Prior radiotherapy or radiosurgery must have been completed at least 2 weeks prior to the first dose of study drug
12. Women of childbearing potential participating in the study (WOCBP see Appendix D for definition) must have a negative serum or urine pregnancy test (minimum sensitivity 25 IU/L or equivalent units of HCG) within 24 hours prior to the start of study drug.
13. WOCBP must agree to follow instructions for method(s) of contraception for the duration of treatment with study drugs plus 5 half-lives of the drugs they are taking at treatment completion (5 times the half-life = 125 days [nivolumab]; 5 times the half-life = 90 days [ipilimumab]; 5 times the half-life = 40 hours [dabrafenib]; 5 times the half-life = 50 days [trametinib]) plus 30 days (duration of ovulatory cycle).
14. Males who are sexually active with WOCBP must agree to follow instructions for method(s) of contraception for the duration of treatment plus 5 half-lives of the study drug as above plus 90 days (duration of sperm turnover).
15. Azoospermic males and WOCBP who are continuously not heterosexually active are exempt from contraceptive requirements. However, WOCBP

participating in the study who are continuously not heterosexually active must still undergo pregnancy testing as described in Appendix D.

Table 2 Laboratory values for adequate organ function

System	Laboratory Values
Haematologic	
Haemoglobin	≥ 9 g/dL
White blood count	≥ 2 $x10^9$/L
ANC	≥ 1.2 $x10^9$/L
Platelet count	≥ $75x10^9$/L
Hepatic	
Albumin	≥ 2.5 g/dL
Total bilirubin[a]	≤ 1.5 x ULN
AST and ALT	≤ 2.5 x ULN
Renal	
Calculated creatinine clearance[b]	≥ 30ml/min
Cardiac	
Left Ventricular Ejection fraction (LVEF)	≥ LLN by ECHO

Abbreviations: ALT = alanine transaminase; ANC = absolute neutrophil count; AST = aspartate aminotransferase; LLN = lower limit of normal;; ULN = upper limit of normal.

a. Except if the patient has Gilbert Syndrome or liver metastases, in which case the bilirubin must be <3 x ULN
b. Calculate creatinine clearance using standard Cockcroft-Gault formula or Wright formula (Appendix C). Creatinine clearance must be ≥50 mL/min to be eligible.

7.2 Exclusion criteria

Deviations from exclusion criteria are not allowed because they can potentially jeopardise the scientific integrity of the study, regulatory acceptability or patient safety. Therefore, adherence to the criteria as specified in the protocol is essential.

Patients meeting **any** of the following criteria must not be enrolled in the study:

1. Prior systemic anti-cancer treatment (immune therapy, targeted therapy, vaccine therapy, or investigational treatment) for Stage III or Stage IV (metastatic) melanoma.
2. Patients receiving prior adjuvant therapy should be excluded if:
    - Adjuvant therapy with combination PD-1 inhibitor plus CTLA-4 inhibitor.
    - Received prior adjuvant therapy with PD-1 inhibitor treatment, which was stopped due to grade 3 or 4 toxicity.

- Received prior adjuvant therapy with BRAF/MEK inhibitor **if** relapse occurred < 6 months from discontinuation of treatment or treatment stopped due to grade 3 or 4 toxicity.

3. Current use of a prohibited medication as described in Section 13.8
4. History of another malignancy. **Exception:** Patients who have been disease-free for 3 years, (i.e. patients with second malignancies that are indolent or definitively treated at least 3 years ago) or patients with a history of completely resected non-melanoma skin cancer. No additional therapy should be required whilst the patient is on study.
5. Any serious or unstable pre-existing medical conditions (aside from malignancy exceptions specified above), psychiatric disorders, or other conditions that could interfere with the patient's safety, obtaining informed consent, or compliance with study procedures.
6. Known Human Immunodeficiency Virus (HIV), Hepatitis B Virus (HBV), or Hepatitis C Virus (HCV) infection.
7. A history of glucose-6-phosphate dehydrogenase (G6PD) deficiency.
8. Patients with active, known or suspected autoimmune disease. Patients with type 1 diabetes mellitus, hypothyroidism only requiring hormone replacement, skin disorders (such as vitiligo, psoriasis or alopecia) not requiring systemic treatment, or conditions not expected to recur in the absence of an external trigger will be permitted to enrol.
9. Patients with a condition requiring systemic treatment with either corticosteroids (>10 mg daily prednisone equivalent) or other immunosuppressive medications within 14 days of study drug administration. Inhaled or topical steroids and adrenal replacement steroid doses > 10 mg daily prednisone equivalent are permitted in the absence of active autoimmune disease.
10. Patients with interstitial lung disease that is symptomatic or may interfere with the detection or management of suspected drug-related pulmonary toxicity.
11. Brain metastases and leptomeningeal metastases are excluded unless:
    - Asymptomatic and untreated at presentation, OR
    - Symptomatic lesions have been definitively treated with surgery or stereotactic surgery (whole-brain radiation may be given as adjuvant treatment), and do not require steroids for control of symptoms
    - Symptomatic metastases, treated or untreated, or metastases requiring

steroids to control symptoms, are excluded

12. No enzyme inducing anticonvulsants for ≥ 4 weeks prior to randomisation
13. Coronary syndromes (including myocardial infarction within 6 months or unstable angina)
14. A history or evidence of current ≥ Class II congestive heart failure as defined by the NYHA guidelines with an ejection fraction of <50%
15. Treatment refractory hypertension defined as a blood pressure of systolic> 150 mmHg and/or diastolic > 95 mm Hg on >3 occasions which cannot be controlled by anti-hypertensive therapy;
16. Known cardiac metastases;
17. Uncorrectable electrolyte abnormalities (e.g. hypokalaemia, hypomagnesaemia, hypocalcaemia), long QT syndrome or taking medicinal products known to prolong the QT interval.
18. A history or current evidence/risk of retinal vein occlusion (RVO) or central serous retinopathy (CSR) including presence of predisposing factors to RVO or CSR (e.g., uncontrolled glaucoma or ocular hypertension, uncontrolled hypertension, uncontrolled diabetes mellitus, or a history of hyperviscosity or hypercoagulability syndromes);
19. Known immediate or delayed hypersensitivity reaction or idiosyncrasy to drugs chemically related to the study treatments including monoclonal antibodies, their excipients, and/or dimethyl sulfoxide (DMSO) and/or Polysorbate-80-containing infusions.
20. Females who are pregnant or breast-feeding.
21. Prisoners or patients who are involuntarily incarcerated.
22. Patients who are compulsorily detained for treatment of either a psychiatric or physical (e.g., infectious disease) illness.

8 Trial Procedures

8.1 Patient Identification

Patients will be identified by the multi-disciplinary team as potentially eligible for the study. Patients will be selected from routine clinics within the hospital, after review of their hospital notes. An appropriately qualified investigator or study nurse will inform patients of the nature of the study and give relevant information about the objectives of the research, benefits and risks both verbally and in writing if required.

8.2 Informed Consent

In order to screen participants for the study and to determine the *BRAF* VAF in ctDNA, patients will be required to sign the main study Informed Consent Form. Once BRAF VAF mutation level is confirmed and if all eligibility criteria are met the patient can be randomised. A second translational sample consent form should be signed by the patient if they are happy to provide optional translational research samples.

Potential patients will have enough time to read the patient information sheet and decide if they would like to participate in the study. At the initial clinic visit, patients will be given telephone numbers to contact the study investigators should they have further questions regarding the study. Participants must have the capacity to provide fully informed consent.

The investigator, or a person designated by the investigator, should fully inform the patient or the patient's legally acceptable representative or legal guardian, of all pertinent aspects of the study and of any new information relevant to the patient's willingness to continue participation in the study. This communication should be documented. If verbal translation is required, a hospital or independent interpreter will be used.

An informed consent form must be signed and personally dated by the patient or the patient's legally acceptable representative and by the person who conducted the informed consent discussion. It will include all elements required by GCP; a description of the study, its purpose, medical implications, alternatives, and possible risks and

benefits. The informed consent form will adhere to the ethical principles that have their origin in the Declaration of Helsinki.

The informed consent will be revised whenever important new information becomes available that is relevant to the patient's consent.

One copy of the consent form will be signed by the patient and Investigator, or suitably qualified delegated individual, the original will be stored in the Investigator Site File and one copy made for the patient to keep and one copy to be filed in the clinical patient records.

Participation in this study is completely voluntary and will not affect the quality of care provided to participants or options to pursue alternative treatments. Participants must not participate in any therapeutic trials whilst on study treatments, however once all study treatments as specified by trial schema are completed (n.b patients may progress on more than 1 treatment within the schema), participation in further studies will not be affected. The patient will continue to be followed up in terms of survival data until they have died, withdraw consent or are lost to follow up.

The rights, safety, and well-being of the study participants are the most important considerations and should prevail over interests of science and society.

8.3 Screening Procedures

Screening logs should be completed for every patient considered for the trial. Copies of these logs should be sent to the Manchester CTU upon request. The main study is Informed Consent Form to allow screening assessments to be conducted and including ctDNA testing, and must be signed before performing any protocol-related procedures that are not part of normal patient care.

The screening visit should be performed **no more than 28 days** prior to randomisation to confirm eligibility (please see Section 9 for list of trial assessments):

8.4 Randomisation

8.4.1 Sequence generation

Randomisation will be done centrally via the King's College London (KCL) independent randomisation service. Patients will be equally randomised to either treatment arm using minimisation with a random element controlling for LDH (<ULN vs. ≥ULN), disease site (<3 sites vs. ≥3 sites), ctDNA VAF (1.5 to ≤10%, >10 to ≤20%, >20%) and M stage (stage III unresectable plus M1a, M1b, M1c and M1d).

A random trial arm allocation will be computer generated by the randomisation provider.

To randomise a patient go to:
**http://ctu.co.uk**

8.4.2 Allocation concealment mechanism and implementation of the allocation sequence.

The responsibility for enrolling and randomising participants into the trial lies with the Principal Investigator and staff at site. Eligibility decisions will be made in line with the approved protocol. Only members of the research team at each participating site who appear on the trial delegation log will have access to the online system and be able to randomise participants. The users will have to confirm the eligibility criteria before they enter the minimisation factors. Then the system will allocate the randomised treatment. Users and the Manchester CTU will then be notified via email of the assigned treatment allocation and the participant number. Allocation concealment will be ensured, as the service will not release the randomisation code until the patient has been recruited into the trial, which takes place after all baseline measurements have been completed.

Please see the CAcTUS randomisation procedure for full details of how to randomise a patient.

8.5 Blinding

Due to the nature of the intervention, trial participants, treating clinicians, pharmacists and research staff will not be blinded to treatment allocation. The trial Statistician will not be blinded either.

8.6 Treatment discontinuation

Participants may withdraw from trial treatment but remain in follow-up.

Specific reasons for discontinuing a participant from study treatment are:

- Investigator decision
- Safety reasons (For example: intolerable toxicity)
- Intercurrent illness
- Severe non-compliance to protocol as judged by the investigator and/or sponsor
  Radiological disease progression as per RECIST v.1.1 when patient has received all study treatments as specified in Appendix A.
- Protocol non-compliance with study treatments (dose reductions as specified in the protocol are acceptable)
- Incorrect enrolment i.e., the participant does not meet the required inclusion/exclusion criteria for the study
- Pregnancy
- Patient decision to discontinue study treatment (see Section 8.7)
- Completed visits up to End of Trial time point

If a participant wishes to withdraw from trial treatment, participating sites should nevertheless explain the importance of remaining on trial follow up for the purposes of continued monitoring of safety, disease status (if pre-progression) and patient status for the individual, along with any subsequent therapies.

8.7 Trial withdrawal

In consenting to the trial, participants are consenting to trial treatment, trial follow-up and data collection. Participants may withdraw from the trial at any time.

Participants may

- Completely withdraw from the trial either by their own choice or be withdrawn due to clinical factors following discussion with their treating clinician
- Be lost to follow-up

If withdrawal of consent to take part in the trial is confirmed, the participant would continue clinic visits as part of subsequent standard of care. Withdrawal from the study for any reason, including investigator decision, requires an End of Study CRF to be completed and sent in within the normal CRF return timelines. Participants do not have to give a reason for their withdrawal but sites should make a reasonable attempt to find out why. Data collected prior to withdrawal from study will be used as part of analyses.

In the unlikely event that a patient is withdrawn from the study due to toxicity during Targeted therapy and does not receive the first cycle of Immune therapy on Arm B, another patient will be randomised to account for the loss and maximise the number of patients receiving the allocated treatment. It is estimated that no more than 3 patients will be withdrawn from the study for this reason.

8.8 Withdrawing consent from ancillary studies

As providing Translational Research samples is an optional part of the trial, participants who have provided consent for this may also choose to withdraw their consent to provide these samples but continue trial treatment and remain in follow-up.

8.9 Trial closure

For the purposes of the Research Ethics Committee approval, the study end date will be declared once the sample analysis is completed. After this point, data on survival will be obtained from the participants GP or NHS Digital.

8.10 Notification of deaths

All deaths will be notified to the Manchester CTU irrespective of whether the death is related to disease progression, IMP, or an unrelated event.

8.11 Lost to follow up

If a participant is lost to follow up the Manchester CTU will request that the PI contacts the participant's General Practitioner (GP) to obtain information on the participant's survival status. Cause and date of death will be requested from the participant's GP. Lost to Follow-up would only apply where all efforts to contact the patient and their GP have failed.

9 Treatment schedules

9.1 Scheduled Visits

Clinical evaluations of participants will be carried out at the following time points. All scheduled visits should be completed within a ±/-3 day window. Please see Schedule of Assessments in Section 9.5

- Screening visit
- Baseline visit
- Arm A (Standard) – During targeted therapy treatment, assessments during D+T made at Wk2 and Wk4 and then every 4 weeks until progression. During immune therapy (N+I) treatment, assessments during N+I made on Day 1 of each cycle.
- Arm B (ctDNA Guided Switch) – Assessments during first D+T made at Wk2 and Wk4 then every 4 weeks until BRAF VAF decrease ≥80%. Assessments during N+I made on Day 1 of each cycle. If progression then 4 weekly when switched back to D+T.
- When participant reaches End of Treatment or withdraws from study
- Follow-up visits – Every 3 months for the first 3 years and then every 6 months either in clinic or via telephone call until 3 months after the last participant is randomised. Participants will therefore be followed up for varying lengths of time depending on when they entered the study. Date of disease progression is only required where participant discontinued treatment prior to progression. Subsequent treatment data and survival status to be collected.

If a patient experiences treatment related toxicity and as a result does not receive the first cycle of Immune therapy (Nivolumab and Ipilimumab combination therapy), the Chief Investigator or Trial Manager must be contacted to confirm this and the relevant completed CRFs and Adverse Event Reports must be returned to MCTU.

9.2 Screening visit

The following procedures will be performed at the participants screening visit.

- Medical history and physical examination including ECOG performance status and vital signs
- Concomitant medications recorded and checked against prohibited medications. If the patient is taking any prohibited medications these should be stopped and/or alternatives found.
- CT/dual phase MRI of chest, abdomen, pelvis (± neck as clinically indicated) and/or other RECIST v1.1 compatible imaging (see Appendix A)
- CT (+ neck if indicated)/MRI head
- Electrocardiograph (ECG)
- Blood tests
    - ctDNA
    - Full blood count
    - Renal and liver function tests
    - Urea and electrolytes
    - Thyroid function tests
    - LDH
    - CRP
- Pregnancy test where required for WOCP
- BRAF status confirmed
- ctDNA VAF $\geq$1.5% confirmed (sample taken at screening but result will be confirmed subsequent to the screening visit and prior to randomisation)
- Clinical disease assessment
- Eligibility assessment
- Additionally, a complete list of all prior anti-cancer therapies will be recorded in the CRF.
- Discussion with the patient and decision as to whether would receive targeted therapy (D+T) or immune therapy (N+I) if randomised to arm A. Decision should be made prior to randomisation.

IMPORTANT: If the patient is too unwell or wants to start targeted treatment without waiting for the screening result, then the screening ctDNA blood can be sent to CBC labs and the patient can start on targeted therapy prior to the screening tests, including the ctDNA bloods, being reported.
Screening tests and the ctDNA result must be performed and reported prior to randomisation. The patient should be counselled that they may still screen out of the study.
The day of or day before (-1 day) the start of targeted therapy is the baseline visit. It is essential that the ctDNA blood is taken on the day of this visit prior to treatment and that all the other baseline assessments (see section 9.1) should be performed within -7 days.
If the patient does not start on targeted therapy on the day the screening ctDNA blood is sent, then a baseline ctDNA blood should be sent on the day of or day before (-1 day) the patient starts treatment (baseline visit). The week 2 ctDNA test will be compared to the ctDNA taken closest to when the patient commences targeted treatment i.e. the baseline.
Blood for ctDNA MUST be taken prior to commencing targeted therapy.

If it is planned that the patient would start immune therapy if screens out of CAcTUS or if randomised to arm A then it is recommended (but not mandatory) that a chair is booked for >7 days following screening blood being taken in order to prevent delay to start of treatment. Any patient who commences immune therapy prior to randomisation will not be able to participate in the CAcTUS trial.

9.3 Baseline visit

The following procedures will be performed at the participant's baseline visit: *However, if the screening and baseline visits are done together (for participants who commence Dabrafenib and Trametinib immediately) then assessments for both visits will be performed at the same time. The baseline visit ctDNA blood MUST be taken on the day of, or day before (-1), commencing targeted therapy. Other assessments need not be repeated as long as they were performed at a screening visit within 7 days of*

starting therapy. Note – if Screening and Baseline visit is the same day, 4 x ctDNA tubes are to be taken.

- Physical examination including medical status, ECOG performance status andvital signs.
- Blood tests
    - ctDNA
    - Full blood count
    - Renal and liver function tests
    - Urea and electrolytes
    - Thyroid function tests
    - LDH
    - CRP
    - Immuno-phenotyping (4x 10ml EDTA) – Christie Site Only
    - Cytokines (1x 10ml EDTA)
- ECHO (to be performed within +/- 28 days of randomisation)
- Radiological assessment (to be performed within 28 days of randomisation)

9.4 On-Treatment Procedures

(Please also see Schedule of Assessments – Section 9.4)

The following procedures will be performed at the participant visits whilst on study treatment:

- Physical examination including ECOG performance status and vital signs
- Concomitant medication recorded and checked against prohibited medications. If patient taking any prohibited medications these should be stopped and/or alternatives found.
- CT/MRI head
- Electrocardiograph (ECG)
- Blood tests
    - ctDNA

- Full blood count
- Renal and liver function tests
- Urea and electrolytes
- Thyroid function tests
- LDH
- CRP
- Research samples (please refer to laboratory manual)

- AE and Toxicity assessment
- Pregnancy test for WOCP
- **Every 8 weeks on therapy:** CT/dual phase MRI of chest, abdomen, pelvis (± neck as clinically indicated) and/or other RECIST v1.1 compatible imaging (see Appendix A)
- Any subsequent anti-cancer therapy taken after study treatment discontinuation should also be documented until study completion/withdrawal or death.

Arm A

Adverse event/toxicity assessments

An adverse event/toxicity assessment will be completed at baseline, the 2nd week and 4 week assessment visits on D+T, every 3 weeks on combination immune therapy and every 4 weeks on nivolumab alone i.e. in line with standard of care clinical assessment.

Radiological assessments

A CT, MRI or PET scan should be completed every 8 weeks (+/- 1 week) from randomisation to assess efficacy.

ctDNA blood tests

Mandatory blood samples for ctDNA analysis will be taken at baseline, the 2nd week and 4 week assessment visits on D+T, every 3 weeks on combination immune therapy and every 4 weeks on nivolumab alone i.e. in line with standard of care clinical assessment.

Arm B

Adverse event/toxicity assessments

An adverse event/toxicity assessment is completed at 2 weeks and then repeated every 4 weeks whilst receiving targeted therapy. Upon switch to immune therapy, AEs/toxicity will be assessed at the same time as scheduled treatment (every 3 weeks for combination therapy and every 4 weeks for nivolumab alone).

Radiological assessments

A CT, MRI or PET scan should be completed every 2 months to assess efficacy whilst on D+T. Prior to the switch to immune therapy a CT, MRI or PET scan will be performed in order to subsequently assess response to immune therapy. Radiological assessment will be performed every 8 weeks (+/- 1 week)while on immune therapy. If progression is confirmed on the CT, MRI or PET CT scan then the patient will switch back to targeted therapy following a 7 day wash out from date of last infusion and remain on treatment until progression or unacceptable toxicity.

ctDNA blood tests

Mandatory blood samples for ctDNA analysis will be taken at baseline, the 2nd week and 4 week assessment visits on D+T, every 3 weeks on combination immune therapy and every 4 weeks on nivolumab alone i.e. in line with standard of care clinical assessment.

9.5 Schedule of trial treatments and assessments

Arm A and B– Pre-treatment Schedule of Assessments

	Screening [A]	Prior to randomisation	Baseline
Informed consent	X		
ctDNA bloods	X		X[B]
Immuno-phenotyping bloods (PBMCs)[C]			X
Cytokine bloods[D]			X
BRAF status confirmed	X		
ctDNA VAF ≥1.5% confirmed		X	
Medical history including comorbidities	X		
ECOG	X		X
Physical exam and vital signs	X		X
ECG	X		
Concomitant medication	X		X
Clinical disease assessment	X		X
Biochemistry [E]	X		X
Thyroid function tests	X		
Haematology	X		X
Renal Function	X		X
Eligibility check	X	X	
ECHO			X
Radiological assessment [F] to be performed within 28 days of randomisation			X
CT (+neck if indicated) and MRI Head	X		
Pregnancy test	X		X
Archival tumour block		X	
Optional microbiome samples			X
Optional research biopsy			X

A. If patients commencing treatment immediately then all the screening assessments should be performed at the same time as baseline assessments

B. x4 Streck tubes

C. Christie patients only – 4 x 10ml EDTA

D. 1 x 10ml EDTA

E. Biochemistry includes LDH and CRP, U+Es, LFTs, TFTs, calcium, **Mg, phosphate, amylase and lipase**

F. Can be either CT Head, TAP, MRI or PET-CT

Arm A (standard therapy) – on treatment schedule of assessments

Patients commencing targeted therapy first (investigator choice)

	**Dabrafenib and Trametinib - assessments made at 2 weeks then every 4 weeks until progression**						**7 day wash out**	**Nivolumab + Ipilimumab every 3 weeks for 4 cycles**				**Nivolumab every 4 weeks until 2nd disease progression[K]**					**2nd Disease progress ion**	**Grade 3/4 immune-related toxicity**	**End of treatment/ withdrawal**	**Follow -up[G]**
	**Wk 2**	**Wk 4**	**Wk 8**	**Wk 12**	**Wk 16**	**4 wkly**		**Cyc 1 day 1**	**Cyc 2 day 1**	**Cyc 3 day 1**	**Cyc 4 day 1**	**Cyc1 day1**	**Cyc 2 day 1**	**Cyc 3 day 1**	**Cyc 4 day 1**	**4 wkly**				
ctDNA bloods	X	X	X	X	X	X		X[B]	X	X	X	X	X	X	X	X	X[B]	X	X	
Immuno-phenotyping bloods (PBMCs) [A]								X[D]	X	X	X						X	X[E]	X[J]	
Cytokine bloods –	X							X[D]	X	X	X						X	X[E]	X[J]	
ECOG	X	X	X	X	X	X		X	X	X	X	X	X	X	X	X		X	X	
Physical exam and vital signs	X	X	X	X	X	X		X	X	X	X	X	X	X	X	X		X	X	
ECG		X	X	X	X	X														
Concomitant medication	X	X	X	X	X	X		X	X	X	X	X	X	X	X	X				
Clinical disease assessment if clinically indicated		X	X	X	X	X		X	X	X	X	X	X	X	X	X			X	
Biochemistry [H]	X	X	X	X	X	X		X	X	X	X	X	X	X	X	X		X	X	
Thyroid function tests								X		X		X	X	X	X	X		X	X	
Haematology	X	X	X	X	X	X		X	X	X	X	X	X	X	X	X		X	X	
Renal Function	X	X	X	X	X	X		X	X	X	X	X	X	X	X	X		X	X	
AEs and toxicity assessment	X	X	X	X	X	X		X	X	X	X	X	X	X	X	X		X	X	
ECHO (every 12 weeks on D&T)				X																
Radiological assessment CT (+neck if indicated)/MRI Head[C]	X[C]<br>X[CK]<br><br>X[CK]																			
Pregnancy test		X	X	X	X	X		X	X	X	X	X	X	X	X	X				

	Dabrafenib and Trametinib - assessments made at 2 weeks then every 4 weeks until progression						7 day wash out	Nivolumab + Ipilimumab every 3 weeks for 4 cycles				Nivolumab every 4 weeks until 2nd disease progression[K]					2nd Disease progress ion	Grade 3/4 immune-related toxicity	End of treatment/ withdrawal	Follow -up[G]
Optional ctDNA research bloods refer to lab manual for specific blood tests							X[I]													
Optional microbiome samples, colonoscopy samples (if performed for toxicity assessment)								X[D]									X	X		
Optional research biopsy								X[D]									X			
Date of disease progression on each therapy																				X[F]
Subsequent treatment data																				X
Survival status																				X

A. Christie patients only

B. x4 Streck tubes

C. To be done every 8 weeks (+/- 1 week)

D. Pre immune therapy (progression on D+T)

E. Optional – to be taken on diagnosis and 5 days post immune suppressive therapy

F. Follow-up for progression would only apply where patient discontinued all treatments prior to progression

G. Follow up every 3 months until 3 months after last patient randomised

H. Biochemistry includes LDH and CRP, U+Es, LFTs, calcium, Mg, phosphate, amylase and lipase

I. Christie only and 1x10ml streck tube alternate days during washout

J Optional

K. Nivolumab monotherapy to be administered 6 weeks after final N+I administration as per SmPC

Patients commencing immune therapy first (investigator choice)

	Nivolumab + Ipilimumab every 3 weeks for 4 cycles				Nivolumab every 4 weeks until disease progression[K]					7 day wash out	Dabrafenib and Trametinib - assessments made at 2 weeks then every 4 weeks until progression						Disease progression (1st or 2nd)	Grade 3/4 immune-related toxicity	End of treatment/ withdrawal	Follow-up[G]
	Cyc 1 day 1	Cyc 2 day 1	Cyc 3 day 1	Cyc 4 day 1	Cyc1 day1	Cyc 2 day 1	Cyc 3 day 1	Cyc 4 day 1	4 wkly		Day 0	Wk 2	Wk 4	Wk 8	Wk 12	4 wkly				
ctDNA bloods	X	X	X	X	X	X	X	X	X		X[B]	X	X	X	X	X	X[B]	X	X	
Immuno-phenotyping bloods (PBMCs) [A]	X[D]	X	X	X													X	X[E]	X[J]	
Cytokine bloods –	X[D]	X	X	X							X						X	X[E]	X[J]	
ECOG	X	X	X	X	X	X	X	X	X		X	X	X	X	X	X		X	X	
Physical exam and vital signs	X	X	X	X	X	X	X	X	X		X	X	X	X	X	X		X	X	
ECG											X	X	X	X	X	**X**				
Concomitant medication	X	X	X	X	X	X	X	X	X		X	X	X	X	X	X				
Clinical disease assessment if clinically indicated	X	X	X	X	X	X	X	X	X		X	X	X	X	X	X			X	
Biochemistry **H**	X	X	X	X	X	X	X	X	X		X	X	X	X	X	X		X	X	
Thyroid function tests	X		X		X	X	X	X	X									X	X	
Haematology	X	X	X	X	X	X	X	X	X		X	X	X	X	X	X		X	X	
Renal Function	X	X	X	X	X	X	X	X	X		X	X	X	X	X	X		X	X	
AEs and toxicity assessment	X	X	X	X	X	X	X	X	X		X	X	X	X	X	X		X	X	
ECHO (every 12 weeks on D&T)															X					
Radiological assessment CT (+neck if indicated)/MRI Head[C]											X[C]<br>X									
Pregnancy test	X	X	X	X	X	X	X	X	X		X	X	X	X	X	X				

Optional ctDNA research bloods refer to lab manual for specific blood tests										X[I]										
Optional microbiome samples, colonoscopy samples (if performed for toxicity assessment)											X[L]						X	X		
Optional research biopsy											X[L]						X			
Date of disease progression on each therapy																				X[F]
Subsequent treatment data																				X
Survival status																				X

A. Christie patients only

B. x4 Streck tubes

C. To be done every 8 weeks (+/- 1 week)

D. Pre immune therapy

E. Optional – to be taken on diagnosis and 5 days post immune suppressive therapy

F. Follow-up for progression would only apply where patient discontinued all treatments prior to progression

G. Follow up every 3 months until 3 months after last patient randomised

H. Biochemistry includes LDH and CRP, U+Es, LFTs, calcium, Mg, phosphate, amylase and lipase

I. Christie only and 1x10ml streck tube alternate days optional during washout

J Optional

K. Nivolumab monotherapy to be administered 6 weeks after final N+I administration as per SmPCL. Pre targeted therapy (progression on immune therapy)

Arm B (ctDNA guided switch) – On treatment Schedule of Assessments

Table 4 Arm B Schedule of Assessments (Part 2)

	Dabrafenib and Trametinib - assessments made at 2 weeks then every 4 weeks until *BRAF* VAF decrease ≥ 80%						7 day wash out	Nivolumab + Ipilimumab every 3 weeks for 4 cycles				Nivolumab 4 weekly till progression /toxicity then switch back to Dabrafenib and Trametinib 4 weekly if disease progression (conduct same assessments as per D+T schedule at 2 weeks then every 4 weeks)[L]					1st and 2nd Disease progression	Grade 3/4 immune-related toxicity	End of treatment / withdrawal	Follow-up[G]
	Wk 2	Wk 4	Wk 8	Wk 12	Wk 16	4 wkly		Cyc 1 day 1	Cyc 2 day 1	Cyc 3 day 1	Cyc 4 day 1	Cyc1 day1	Cyc 2 day 1	Cyc 3 day 1	Cyc 4 day 1	4 wkly				
ctDNA bloods	X	X	X	X	X	X		X[B]	X	X	X	X	X	X	X	X	X[B]	X	X	
Immuno-phenotyping bloods (PBMCs)[A]								X[D]	X	X	X						X	X[E]	X[K]	
Cytokine bloods	X							X[D]	X	X	X						X	X[E]	X[K]	
ECOG	X	X	X	X	X	X		X	X	X	X	X	X	X	X	X		X	X	
Physical exam and vital signs	X	X	X	X	X	X		X	X	X	X	X	X	X	X	X		X	X	
ECG		X	X	X	X	X														
Concomitant medication	X	X	X	X	X	X		X	X	X	X	X	X	X	X	X				
Clinical disease assessment if clinically indicated		X	X	X	X	X		X	X	X	X	X	X	X	X	X	X		X	
Biochemistry[H]	X	X	X	X	X	X		X	X	X	X	X	X	X	X	X		X	X	
Thyroid function tests								X		X		X	X	X	X	X		X	X	
Haematology	X	X	X	X	X	X		X	X	X	X	X	X	X	X	X		X	X	
Renal Function	X	X	X	X	X	X		X	X	X	X	X	X	X	X	X		X	X	
AEs and toxicity assessment	X	X	X	X	X	X		X	X	X	X	X	X	X	X	X		X	X	

	**Dabrafenib and Trametinib - assessments made at 2 weeks then every 4 weeks until *BRAF* VAF decrease ≥ 80%**						**7 day wash out**	**Nivolumab + Ipilimumab every 3 weeks for 4 cycles**				**Nivolumab 4 weekly till progression /toxicity then switch back to Dabrafenib and Trametinib 4 weekly if disease progression (conduct same assessments as per D+T schedule at 2 weeks then every 4 weeks)[L]**					**1st and 2nd Disease progression**	**Grade 3/4 immune-related toxicity**	**End of treatment / withdrawal**	**Follow-up[G]**
ECHO (every 12 weeks on D+T)				X		X														
Radiological assessment CT (+neck if indicated)/MRI Head[C]	X[C]																			
Pregnancy test		X	X	X	X	X		X	X	X	X	X	X	X	X	X				
Optional ctDNA research bloods refer to lab manual for specific blood tests							X[J]												X	
Optional microbiome samples, colonoscopy samples (if performed for toxicity assessment)								X[D]									X[I]	X[E]		
Optional research biopsy								X[D]									X			
Date of disease progression on each therapy																				X[F]
Subsequent treatment data																				X
Survival status																				X

A. Christie patients only
B. x4 Streck tubes
C. To be done every 8 weeks (+/- 1 week)
D. Pre immune therapy
E. Optional to be taken on diagnosis and 5 days post immune suppressive therapy
F. Follow-up for progression would only apply where patient discontinued all treatments prior to progression
G. Follow up every 3 months until 3 months after last patient randomised
H. Biochemistry includes LDH and CRP, U+Es, LFTs, calcium, Mg, phosphate, amylase and lipase
I. Progression on immune therapy only
J. Christie only
K. Optional
L. Nivolumab monotherapy to be administered 6 weeks after final N+I administration as per SmPC

10 Collection, processing and storage of biospecimens

10.1 Collection of biospecimens

Please refer to the Lab manual for detailed information and instructions

As well as taking blood for the ctDNA analysis, the following specimens may be collected as part of consent to the trial.

- Additional blood samples
- Archival formalin fixed paraffin embedded (FFPE) tumour specimen from a previous biopsy or surgical procedure.
- Fresh biopsy of easily accessible lesions (up to a maximum of three), at baseline prior to immune therapy and if progression on therapy. Patients may consent to any or all of these biopsies. If insufficient material is available patients will not be subjected to an additional biopsy
- If a surgical resection on study occurs for clinical reasons then provided the patient has consented this tissue should also be made available for translational research with collection as below
- Microbiome samples (stool)

10.2 Sample collection and handling

An outline for the collection, processing, storage and shipping of samples to Manchester are specified below. **Please refer to the lab manual for more detailed instructions.**

10.3 Mandatory samples

The following samples will be collected from patients:

10.3.1 Blood

Blood collected in Cell-Free DNA BCT Streck tubes for ctDNA analysis

For both arms of the study, at scheduled time points detailed in Sections 9.4 and 9.4 (On-treatment procedures and schedule of trial treatments and assessments) up to 40 ml of blood (see laboratory manual for further information) will be collected in Cell-Free DNA BCT Streck tubes (provided by trial coordinators).

Bloods collected **in streck tubes** must be sent to CBC laboratories by royal mail safe box system provided by the trial coordinator within 24 hours of blood draw– see lab manual for address.

Samples taken at The Christie NHS Foundation Trust may be sent with the Cancer Research UK (CRUK)-Manchester Institute (MI) porters for transfer to CRUK- Cancer Biomarker Centre (CBC) lab.

Sites must complete the form provided in Appendix 1 of the lab manual to send with the blood sample. The information on the tube must match the information given on the form.
To preserve patient confidentiality, samples will be allocated a study ID number and all samples entering the laboratories will be logged within the LIMS system.
Blood should be collected for cytokine and metabolon analyses in EDTA tubes (10ml). Please refer to the lab manual **NB these bloods must be processed within 1 hour of collection**.

Christie NHS Foundation Trust patients only
Bloods for PBMCs will be collected in EDTA tubes (10ml) and must be sent with the CRUK-MI porters for transfer to the CBC laboratory immediately for processing

Additional bloods taken during the washout period in Cell-Free DNA BCT Streck tubes can be sent using the CRUK-MI porters.

10.3.2 Tissue

Archival FFPE tumour tissue will be requested for all patients. Archival samples may be from either primary or metastatic tumour.

10.4 Optional samples

10.4.1 Blood

Additional samples of blood for translational analyses will be collected in Cell-Free DNA BCT Streck tubes. These should be sent with the mandatory blood samples on the day of taking – see Section 10.3.1.

10.4.2 Tissue

Fresh biopsies will be obtained according to optional patient consent, as set out in the sample schedule. Biopsies will only be acquired if the lesions are considered to be safely accessible by the interventional radiologist or surgeon. Ideally the same site of disease should be biopsied on each occasion. Please refer to the lab manual for prioritising and initial processing of tissue samples.

10.4.3 Microbiome sample collection (stool)

Patients should be supplied with sample collection container, sealable bag and collection, example instructions available at http://www.nhs.uk/chq/Pages/how-should-i-collect-and-store-a-stool-faeces-sample.aspx?CategoryID=69.

Samples to be collected at home by patient <24 hours before subsequent appointment when the sample will be dropped off. If sample taken for toxicity it should be collected at time of diagnosis or on admission to hospital.

Once collected samples should be stored at -80°C (-20°C if -80°C not available). These can be batched for sending on dry ice – see lab manual for details.

10.5 Storage and disposal of biospecimens

All collected biospecimens will be stored in designated and secure facilities within the CBC or Manchester Cancer Research Centre (MCRC) Biobank. Frozen tissue and plasma/blood pellet samples will be stored in temperature monitored, -80ºC freezers. Archival FFPE specimens will be stored within dedicated areas within the CBC laboratory. All samples will be barcoded, logged and tracked within a LIMS system.

Plasma samples for ctDNA analysis will be sent to the CRUK CBC lab using Royal Mail post. Other biological samples will be stored at site for up to 12 months before being transferred by courier to the MCRC Biobank in Manchester.

The Chief Investigator will serve as custodian of samples for the duration of this study. Samples will be stored at the CBC laboratory and Manchester Cancer Research Centre (MCRC) Biobank. Sample usage will be under the control and jurisdiction of the Chief Investigator and Principal Investigators. Samples will be stored at site as per the Lab Manual for a period of up to 12 months before being transferred by courier to relevant laboratories for analysis. The receiving laboratory will act as temporary custodian for those samples. Clinical samples will be kept for up to 10 years and any surplus to the study requirements may be used for future translational research authorised by the trial management committee.

Disposal of biospecimens will be considered under certain circumstances including but not limited to use of the entire specimen; withdrawal of patient consent for storage and analysis of samples (obtained archival samples will be returned to original pathology lab) or reduced specimen integrity. Determination of the integrity of biospecimens is at the discretion of study personnel. Disposed samples will be logged within the LIMS system (together with reason for disposal) and physically discarded according to institutional policy.

10.6 Sharing of samples with other centres

Samples may be shared with other centres for research directly in connection with the existing protocol or for future translational work. This may include transfer of samples to collaborators in other countries. Where required, additional approvals will be sought to share samples and a Material Transfer Agreement will be put in place before samples are shared. This will be managed with the assistance of our human tissue governance team and R&D office.

11 Circulating tumour DNA processing, results and clinical decision making

Please refer to the lab manual for more detailed information and instructions

11.1 Prioritisation of bloods

On receipt in the lab, bloods will be colour coded according to a “traffic light” system of priority. This does not need to be done at site:

- **Red** = on study arm A (screening sample only) and arm B: These will be patients for whom a clinical decision as to switching treatment based on ctDNA will be critical.

  Blood will be analysed, the results verified and reported to the Chief Investigator and nominated deputy within 7 working days from receipt of sample by the laboratory.

- **Orange** = on study Arm A: blood will be analysed and results verified to inform the trial objectives

- **Green** = on study Arm A or B: blood that will be processed and stored for future translational objectives.

11.2 DNA extraction and analysis using droplet digital

Circulating DNA will be extracted from plasma and analysed using ddPCR technology at the CRUK CBC lab.

11.3 Reporting of ctDNA result and clinical decision procedure

For red priority bloods, the confirmed ctDNA result will be reported by CBC lab staff to the Chief Investigator. The CI, or nominated deputy, will forward the results to the site

trial coordinator by secure email. The site trial coordinator will inform the site PI of the ctDNA result and action to be taken:

- If the ctDNA BRAF VAF has not decreased by ≥80% then the patients will continue on D+T.
- If the ctDNA BRAF VAF has decreased by ≥80% then treatment will switch to nivolumab and ipilimumab; this must be within 14 days +/- 2 days from result notification. A scan and optional research biopsy must be performed during that period. The patient should be informed that they must stop taking trametinib 7 days prior, and dabrafenib 1 day prior, to the start of immune therapy. **A further telephone call on the day of stopping dabrafenib to remind the patient is recommended.** The patient will then commence on N+I as per the trial schedule.

12 Trial Medication

12.1 Study drugs and administration

This a non CTIMP study. Dabrafenib, Trametinib, Nivolumab and Ipilimumab are commercially available and should be sourced locally as per standard practice at the investigator sites using products that have a UK or EU license. Please refer to relevant SmPC for further information.

Descriptive information for the drugs can be found in the package insert. Study treatment should be administered according to the institutional standards at each site. The drugs will be used and stored as detailed on the product label and according to manufacturer's instructions. There will be no re-imbursement to sites for the drugs.

Dabrafenib and trametinib

- Dabrafenib (Tafinlar®) will be administered orally at 150mg twice daily using 50mg or 75mg tablets and trametinib (Mekinst®) at 2mg once daily using 2mg or 0.5mg tablets unless a dose reduction is required (see Section 13).

Nivolumab and ipilimumab

- Nivolumab (Opdivo®) will be administered IV over 60 minutes at 1 mg/kg combined with ipilimumab (Yervoy®) administered IV over 90 minutes at 3 mg/kg every 3 weeks for 4 doses (Part I), and then nivolumab 480mg will be administered IV over 60 minutes every 4 weeks until progression or unacceptable toxicity, whichever comes first.
- Premedications or medications used to treat infusion-related reactions should be sourced by the investigative sites if available and permitted by local regulations. Solution used as diluent solutions (0.9% sodium chloride injection, 5% dextrose injection) should also be sourced by investigative sites if available and permitted by local regulations.
- For dosing calculations based on body weight: if the participant's weight on the day of dosing differs by > 10% from the last weight used to calculate the dose previously, the dose must be recalculated. All doses should be rounded up to the nearest milligram per institutional standard.

During combination nivolumab + ipilimumab (4 cycles), participants may be dosed no less than 19 days between doses. If dosing is delayed, both nivolumab and ipilimumab must be delayed together. If dosing is resumed after a delay, both nivolumab and ipilimumab must be resumed on the same day. If clinically appropriate and toxicity felt to be ipilimumab related then nivolumab alone may be restarted. This must be discussed with the CI.

- During nivolumab as a single agent, participants may be dosed no less than 25 days between doses. Participants may be dosed up to 3 days after the scheduled date if necessary. Subsequent dosing should be based on the actual date of administration of the previous dose of drug.

13 Management of toxicities, dose delays and modifications

All adverse events that occur during the trial should be recorded as detailed in Section 14.2.

All treatments have been approved by the FDA/EMA and investigators should refer to their guidance (including the EU summary of product characteristics) regarding management of toxicities.

13.1 Restrictions

The following restrictions apply while the patient is receiving study treatment and for the specified times before and after:

- Patients should avoid excessive sun exposure and use adequate screening protection. The use of sunbeds and tanning booths should be avoided.

13.2 Dabrafenib and trametinib

13.2.1 General Guidelines for Clinically Significant Toxicities (D+T)

General guidelines regarding management and dose reduction for adverse events that are considered by the investigator to be related to D+T treatment are provided in Table 3.

Table 3 Dose Modification Guidelines for Events Considered Related to Study Treatment (Dabrafenib and Trametinib)

CTCAE Grade	Action and Dose Modification
Grade 1 or Grade 2 tolerable	Continue study treatment at current dose level.<br>Monitor closely<br>Provide supportive care according to institutional standards

Grade 2 (intolerable) or Grade 3	Interrupt study treatment (except for cuSCC, keratoacanthoma, new primary melanoma, and basal cell carcinoma which will be treated according to local guidelines/ treating clinician discretion)<br>Monitor closely<br>Provide supportive care according to institutional standards<br>When toxicity resolves to grade 1 or baseline, restart study treatment reduced by one dose level<br>If the grade 2 (intolerable) or grade 3 toxicity recurs, interrupt study treatment<br>When toxicity resolves to grade 1 or baseline, restart study treatment reduced by another dose level<br>If the clinical investigator feels that toxicity is related to trametinib and that the patient would benefit from continuing dabrafenib alone then this is allowed.
Grade 4	Interrupt study treatment (except for cuSCC, keratoacanthoma, new primary melanoma, and basal cell carcinoma)<br>Monitor closely<br>Provide supportive care according to institutional standards<br>Restart with study treatment reduced by one dose level once toxicity resolves to grade 1 or baseline or permanently discontinue study treatment at discretion of investigator<br>If the clinical investigator feels that toxicity is related to trametinib and that the patient would benefit from continuing dabrafenib alone then this is allowed.

If there is a delay beyond 6 weeks the participant must be withdrawn from the study treatment or discussed with the CI for further guidance on whether they can remain on study treatment.

13.3 Nivolumab and Ipilimumab

13.3.1 Dose delay criteria

Once a participant has started on immune therapy the following dose delay criteria

apply for all drug-related AEs (regardless of whether or not the event is attributed to nivolumab, ipilimumab, or both). All study drugs must be delayed until treatment can resume. If there is a delay beyond 6 weeks the participant must be withdrawn from the study treatment or discussed with the CI for further guidance.

Study drug administration should be delayed for the following:

- Any Grade ≥ 2 non-skin, drug-related adverse event, with the following exceptions: Grade 2 drug-related fatigue or Grade 2 laboratory abnormalities do not require a treatment delay.
- Any Grade 3 skin, drug-related adverse event.
- Any Grade 3 drug-related laboratory abnormality, with the following exceptions for asymptomatic amylase or lipase, AST, ALT, or total bilirubin.:
    - Grade 3 amylase or lipase abnormalities that are not associated with symptoms or clinical manifestations of pancreatitis do not require a dose delay. It is recommended to consult with the Chief Investigator for Grade 3 amylase or lipase abnormalities.
    - If a participant has a baseline AST, ALT, or total bilirubin that is within normal limits, delay dosing for drug-related Grade ≥ 2 toxicity
    - If a participant has baseline AST, ALT, or total bilirubin within the Grade 1 toxicity range, delay dosing for drug-related Grade ≥ 3 toxicity.
    - Any adverse event, laboratory abnormality, or intercurrent illness which, in the judgment of the investigator, warrants delaying the dose of study medication.

13.3.2 Discontinuation Criteria

Discontinuation criteria apply for all drug-related AEs attributed to nivolumab, ipilimumab, or both. For cycles containing both nivolumab and ipilimumab they should be discontinued at the same time. Treatment should be permanently discontinued for the following:

- Any Grade 2 drug-related uveitis, eye pain, or blurred vision that does not respond to topical therapy and does not improve to Grade 1 severity within the re-treatment period OR requires systemic treatment.

- Grade 3 drug-related uveitis, pneumonitis, bronchospasm, diarrhoea, colitis, neurologic toxicity, hypersensitivity reaction, or infusion reaction of any duration requires discontinuation
- Grade 3 drug-related laboratory abnormalities do not require treatment discontinuation except:
    - Grade 3 drug-related thrombocytopenia > 7 days or associated with bleeding requires discontinuation
    - Any drug-related liver function test (LFT) abnormality that meets the following criteria require discontinuation:
        - - AST or ALT>8xULN
        - - Total bilirubin > 5 x ULN
        - - Concurrent AST or ALT > 3 x ULN and total bilirubin > 2 x ULN
- Any Grade 4 drug-related AE or laboratory abnormality, except for the following events which do not require discontinuation:
    - Grade 4 amylase or lipase abnormalities that are not associated with symptoms or clinical manifestations of pancreatitis. It is recommended to consult with the BMS Medical Monitor for Grade 4 amylase or lipase abnormalities.
    - Isolated Grade 4 electrolyte imbalances/abnormalities that are not associated with clinical sequelae and are corrected with supplementation/appropriate management within 72 hours of their onset
- Any dosing interruption lasting > 6 weeks with the following exceptions:
    - Dosing interruptions to allow for prolonged steroid tapers to manage drug-related AEs are allowed. Prior to re-initiating treatment in a participant with a dosing interruption lasting > 6 weeks, the CI must be consulted. Tumourassessments should continue as per protocol even if dosing is interrupted.
    - Dosing interruptions > 6 weeks that occur for non-drug-related reasons may be allowed if approved by the CI. Prior to re-initiating treatment in a participant with a dosing interruption lasting > 6 weeks, the CI must be consulted. Tumour assessments should continue as per protocol even if dosing is interrupted.
- Any adverse event, laboratory abnormality, or intercurrent illness which, in the

judgment of the investigator, presents a substantial clinical risk to the participant with continued nivolumab or ipilimumab dosing.

13.4 Drug supply and distribution

All drugs will be sourced from routine hospital stock and their handling and management will be subject to standard procedures of the pharmacy.

13.5 Storage and dispensing

The study drugs should be stored in accordance with the environmental conditions (temperature, light, and humidity) as determined by the manufacturer (BMS/Novartis). If concerns regarding the quality or appearance of the drugs arise, the study drug should not be dispensed and the manufacturer informed immediately.

Product documentation must be maintained that includes all processes required to ensure drug is accurately administered. This includes documentation of drug storage, administration and, as applicable, storage temperatures, reconstitution, and use of required processes (eg, required diluents, administration sets).

Please refer to the relevant SmPC for further information.

13.6 Treatment adherence

Participants will be instructed to return treatment pills at each visit. Adherence with study treatment will be assessed by querying the participant and through pill count at each visit. Adherence will be documented in the source documents and the CRF.

Interruption of study drug should be avoided. If an infusion is missed participants should be given the next one at the stipulated (pre-planned) time. The reason for any missed dose should be recorded on the CRF.
A record of the number of dabrafenib and trametinib capsules/tablets dispensed to and taken by each participant must be maintained and reconciled with study treatment and compliance records. Treatment start and stop dates, including dates of dose modifications and/or interruptions will also be recorded in the CRF. The investigator will make every effort to bring non-compliant participants into compliance.

13.7 Concomitant medications

The investigator must be informed as soon as possible about any medication taken from the time of screening until 30 days after the last dose of study drug.

Any concomitant medication(s), including dietary supplements, taken during the study will be recorded in the CRF. The minimum requirement is that drug name, dose, and the dates of administration are to be recorded.

13.8 Prohibited Medications

The following medications are prohibited within 28 days or 5 half-lives, whichever is shorter, prior to randomisation and during the study treatment period (unless to treat a drug-related adverse event):

- Any concurrent antineoplastic therapy (i.e., chemotherapy, hormonal therapy, immune therapy, non-palliative radiation therapy, or standard or investigational agents for treatment of cancer)
- Illicit drugs
- Antiretroviral drugs
- Herbal remedies (e.g., St. John's wort)

13.8.1 Medications specifically prohibited on dabrafenib and trametinib

Please see Appendix E. for full list of medications prohibited whilst on D+T.

13.8.2 Medications specifically prohibited on nivolumab and ipilimumab

Immunosuppressive agents (except to treat a drug-related adverse event). Systemic corticosteroids >10 mg daily prednisone equivalent (except as stated in Appendix E permitted therapy to treat drug-related AEs and treatment of infusion reaction)

13.9 Radiation therapy

Limited field palliative radiation therapy for bone pain due to pre-existing bone

metastasis is permitted.

Radiation for isolated progression or symptom relief must be discussed with the CI.

13.10 Surgical resection following initial response

Whilst on nivolumab and ipilimumab only, investigators may choose to resect solitary lesions in participants with unresectable or metastatic melanoma and render the subject free of macroscopic disease. Participants enrolled in this study may have lesions surgically resected only if confirmation of response is documented at least 4 weeks after previous scan and following consultation with the CI. If tumor shrinkage of the solitary lesion is noted on the re-staging assessment, it is highly encouraged that surgical resection be delayed until subsequent scans fail to demonstrate further shrinkage. Participants with a partial response who go on to have surgical resection of remaining disease will be considered a partial responder. Any tissue from surgical resections should be made available for translational research provided the patient has consented.

13.11 Permitted therapy

Corticosteroids during nivolumab and ipilimumab treatment:

- Participants are permitted the use of topical, ocular, intra-articular, intranasal and inhalation corticosteroids (with minimal system absorption). Physiologic replacement doses of systemic corticosteroids are permitted even if >10 mg daily prednisone (or equivalent). A brief course of corticosteroids for prophylaxis (eg, for contrast dye allergy) or for treatment of non-autoimmune conditions (eg, delayed-type hypersensitivity reaction caused by a contact allergen) is permitted.
- Supportive care for disease-related and drug-related symptoms (anti-emetics, anti-diarrhoeals, and analgesics) may be offered to all participants on the trial including blood and blood products.

13.12 Treatment of Study Drug Overdose

Overdoses are defined as the accidental or intentional administration of any dose of a product that is considered both excessive and medically important.

Any overdose should be reported as per Section 14 and the Investigator should contact the CI immediately and closely monitor the participant for AEs/SAEs and laboratory abnormalities.

Dabrafenib and trametinib overdose

- Dabrafenib overdose is defined as administration of more than 300 mg as a single dose or 600 mg per day (the highest dose tested in clinical studies to date),
- Trametinib overdose is defined as administration of more than 3.0 mg once daily (the maximum tolerated dose defined in the MEK111054 Study),
- There is no recommended specific treatment. The investigator will use clinical judgment to treat any overdose. Hemodialysis is not expected to enhance the elimination of either dabrafenib or trametinib as both are highly bound to plasma proteins.

Nivolumab and Ipilimumab overdose

- There is no recommended specific treatment. The investigator will use clinical judgement to treat any overdose.

14 Safety reporting and pharmacovigilance

The Principal Investigator is responsible for ensuring that all site staff involved in this trial are familiar with the content of this section. The following definitions are in accordance with the Medicines for Human Use (Clinical Trials) Regulations 2004 (SI2004/1031) (as amended) and EU Directive 2001/20/EC.

Term	Definition
**Adverse Event (AE)**	Any untoward medical occurrence in a participant to whom a medicinal product has been administered, including occurrences which are not necessarily caused by or related to that product.
**Adverse Reaction (AR)**	An untoward and unintended response in a participant to an investigational medicinal product which is related to any dose administered to that participant.<br>The phrase "response to an investigational medicinal product" means that a causal relationship between a trial medication and an AE is at least a reasonable possibility, i.e. the relationship cannot be ruled out.<br>All cases judged by either the reporting medically qualified professional or the Chief Investigator (acting on behalf of the Sponsor) as having a reasonable suspected causal relationship to the trial medication qualify as adverse reactions.
**Serious Adverse Event (SAE)**	A serious adverse event is any untoward medical occurrence that:<br>• results in death<br>• is life-threatening<br>• requires inpatient hospitalisation or prolongation of existing hospitalisation<br>• results in persistent or significant disability/incapacity<br>• consists of a congenital anomaly or birth defect<br>Other 'important medical events' may also be considered serious if they jeopardise the participant or require an intervention to prevent one of the above consequences.<br>NOTE: The term "life-threatening" in the definition of "serious" refers to an event in which the participant was at risk of death at the time of the event; it does not refer to an event which hypothetically might have caused death if it were more severe.
**Serious Adverse Reaction (SAR)**	An adverse event that is both serious and, in the opinion of the reporting Investigator, believed with reasonable probability to be due to the IMP, based on the information provided.
**Suspected Unexpected Serious**	A serious adverse reaction, the nature and severity of which is not consistent with the information about the medicinal product

Term	Definition
**Adverse Reaction (SUSAR)**	in question set out the summary of product characteristics (SmPC)

*Note: The term 'life-threatening' in the definition of serious refers to an event in which the trial participant was at risk of death at the time of the event or it is suspected that continued use of the product would result in the patient's death; it does not refer to an event which hypothetically might have caused death if it were more severe.

** Note: See exemption criteria in section 14.2.2.

*** Note: other events that may not result in death, are not life-threatening, or do not require hospitalisation, may be considered as an SAE when, based upon appropriate medical judgement, the event may jeopardise the participant and may require medical or surgical intervention to prevent one of the outcomes listed above.

**** Note: to avoid confusion or misunderstanding of the difference between the terms "serious" and "severe", the following note of clarification is provided: "Severe" is often used to describe intensity of a specific event, which may be of relatively minor medical significance. "Seriousness" is the regulatory definition supplied above.

14.1 Detecting and recording AEs and SAEs

The time period for detecting and recording AEs and SAEs will be from the beginning of treatment to 100 days post treatment for both targeted and immune therapy.

14.2 Reporting procedures

14.2.1 Adverse Events

All adverse events that occur between the beginning of treatment to 100 days post treatment in both arms of the study, must be recorded in the patient notes and in the appropriate section of the trial CRF.

AEs meeting the definition of a SAE (please see Section 14) must also be reported to the Manchester CTU as detailed below.

Pre-existing conditions do not qualify as adverse events unless they worsen.

14.2.2 Serious Adverse Events and Notable Events

All SAEs/Notable Events occurring from the beginning of treatment and up to 100 days post treatment must be submitted to the Manchester CTU by email (SAEreport_manCTU@manchester.ac.uk) immediately but within **24 hours** of observing or learning of the event, using the trial specific SAE Notification Form.

All SAEs must be reported to the CAcTUS Trial Manager at the Manchester CTU within 24 hours of becoming aware of the event
Email: SAEreport_manctu@manchester.ac.uk

As many sections of the SAE notification form should be completed as possible (with further information being provided as it becomes available) and sent to the Manchester CTU. An assessment of the seriousness and causality of the SAE must be made by the PI or another delegated doctor.

If the delegated doctor is unavailable, the initial SAE notification form without a causality assessment should be submitted to the Manchester CTU within **24 hours** of observing or learning of the event, but must be follow-up by medical assessment as soon as possible thereafter.

Any change in condition or other follow up information should be reported to the Manchester CTU as soon as it is available or at least within **24 hours** of observing or learning of the change. All SAEs must be followed-up until the event has resolved or a final outcome has been reached. SAEs are being evaluated for duration and intensity according to CTCAE version 5.

For the purposes of this study, the following will also be reported as SAEs:

- Potential drug induced liver injury (pDILI) defined as: AT (ALT or AST) elevation >3 times upper limit of normal (ULN) ***and*** total bilirubin >2 times ULN, without initial findings of cholestasis (elevated serum alkaline phosphatase) ***and*** no other immediately apparent possible causes of AT elevation and hyperbilirubinemia, including, but not limited to, viral hepatitis, pre-existing

chronic or acute liver disease, or the administration of other drug(s) known to be hepatotoxic.

- Overdose defined as the accidental or intentional administration of any dose of a product that is considered both excessive and medically important.

For the purposes of this trial the following events will not require immediate reporting

- A visit to the emergency room or other hospital department < 24 hours, that does not result in admission (unless considered an important medical or life-threatening event)
- Elective surgery, planned prior to signing consent
- Admissions as per protocol for a planned medical/surgical procedure
- Routine health assessment requiring admission for baseline/trending of health status (e.g., routine colonoscopy)
- Medical/surgical admission other than to remedy ill health and planned prior to entry into the study. Appropriate documentation is required in these cases.
- Admission encountered for another life circumstance that carries no bearing on health status and requires no medical/surgical intervention (e.g., lack of housing, economic inadequacy, caregiver respite, family circumstances, administrative reason).

- These should be completed in the participants notes and on the relevant toxicities CRF page and forwarded to the Manchester CTU in the normal timeframes for CRFs.

A Notable Event for the purpose of the trial includes:

- All Grade 3 or 4 infusion reactions

14.2.3 SAE Processing and Notable Event Reporting at the Manchester CTU

On receipt of the SAE Report Form, the CAcTUS CTM will send an acknowledgement of the SAE or Notable Event to the relevant members of the trial team at the participating site. This acknowledgement will include an SAE reference number which should be included on all future correspondence regarding the SAE.

Expectedness assessment

The CTM will liaise with the CI to evaluate the event for seriousness, causality and expectedness to determine whether or not the case qualifies for expedited reporting. The CI or delegate will review the SAE form and determine expectedness in relation to the reference safety information. This will be based on the Reference Safety Information contained in the SmPC for each of the study drugs being administered. If an SAE is suspected to be a SUSAR, regulatory time constraints apply for expedited reporting.

Expectedness of the event to the trial treatment must be determined as follows:

- **Expected** The event is listed as an expected adverse event in the SmPC
- **Unexpected** The event is not listed as an expected adverse event in the SmPC, or, the severity of the event is greater than that listed in the SmPC

Note: If a difference of opinion between the PI and CI gives rise to an event being classified as an SAE by one clinician and a SUSAR by another, the "worst-case" assessment is assumed i.e. it is classified as a SUSAR. Similarly, the causality assessment made by the PI at site cannot be downgraded by the CI as the PI is more familiar with the patient's history, clinical signs and symptoms, lab findings and other investigations. The CI may, however, upgrade the PI's assessment of causality.

Centres should respond as soon as possible but at least within the timeline stipulated to requests from the CI or designated representative (via the Manchester CTU) for further information that may be required for final assessment of the SAE.

The CTM will advise the Christie NHS Foundation Trust as Sponsor and study funder (Bristol Myers Squibb) by email of all SAEs or Notable Events within 24 hours of the Manchester CTU becoming aware of the event. The Manchester CTU will send copies of all SAE and Notable Event forms to BMS Worldwide Safety. The CTM will also keep BMS Worldwide Safety informed of the status of SAEs with regular update emails and also will provide full details of how the SAE has resolved.

14.2.4 SUSAR Processing and Reporting at the Manchester CTU

All SAEs designated as both suspected to be related to the study drug and unexpected will be classified as a SUSARs and will be subjected to expedited reporting. The CTM will inform the REC and Sponsor within the required expedited reporting timelines. SUSARs are reported to the relevant REC by the CTM using the HRA process.

If the SUSAR represents a significant safety concern, all investigators participating in that trial will be informed as soon as possible by the CTM. SUSARs will be notified to sponsors, investigators and third parties in accordance with the trial agreements. SUSARs reported to the CTM from other trials (via drug manufacturers etc.) are disseminated to the sponsor and to all sites using the same trial treatment.

14.3 Pregnancy reporting

If a participant or partner of a participant becomes pregnant, the participant must be withdrawn from the trial IMP immediately. Any pregnancy occurring in a participant or partner of a participant during treatment or within 100 days post treatment must be reported to the Manchester CTU by email (SAEreport_manCTU@manchester.ac.uk) immediately but no later than 24 hours of the site staff becoming aware of it using a Pregnancy Notification Form. Study drug will be stopped immediately. It is the Investigator's responsibility to obtain consent for follow-up from the participant. Pregnancy will be considered an SAE for the purposes of this trial and will be reported to BMS as such, however an SAE report will only be required where this meets the SAE criteria. The Manchester CTU will follow-up all pregnancies for the pregnancy outcome via the Investigator, using a Pregnancy Outcome Form. The Manchester CTU will then inform the sponsor within one working day of receipt. The Manchester CTU will work with the investigator to ensure that all relevant information is provided to the sponsor and manufacturer. Should a pregnancy occur during the trial, the Investigator should offer counselling to the participant, and discuss the risks of continuing with the pregnancy and the possible effects on the foetus. Monitoring of the participant and the baby should continue until the conclusion of the pregnancy.

14.4 Overdose reporting

The use of study medication in doses in excess of that specified in the protocol is considered to be an overdose

Investigators should be advised that any participant who receives a higher dose than that intended should be monitored closely, managed with appropriate supportive care and followed up closely.

All overdoses should be recorded as follows:

- All overdoses should be recorded on the Overdose CRF page.
- An overdose will only need to be reported as an SAE for the purpose of this trial where the event fulfils seriousness criteria.

The completed Overdose CRF page should be reported to the Manchester CTU by email (SAEreport_manCTU@manchester.ac.uk) Manchester **immediately, but no later than 24 hours** of the investigator or site staff becoming aware. The CTM will then inform the sponsor and manufacturer within one working day of receipt. The trial manager will work with the investigator to ensure that all relevant information is provided to the sponsor and manufacturer.

14.5 New Safety Findings

If a new safety finding emerges (from sources such as IMP manufacturers, data analysis, steering committee findings), the CI reviews the finding for its impact on the participants in the relevant trial(s). If there is a potential impact on trial participant's safety, the sponsor takes appropriate action in conjunction with the CTM, CI and research team. Appropriate reporting mechanisms are followed in the event of actions being taken.

14.6 Urgent Safety Measures

Where an urgent safety measure (to prevent immediate hazard to trial participant's health and safety) is necessary, prior authorisations from the HRA are not required.

Where the PI takes urgent action that is not consistent with the protocol to prevent harm to a participant on a trial, the PI must immediately inform the CI / CTM and give full details of the measures taken and the decision making process surrounding the action(s) taken. The CTM will inform the CI, sponsor, and the HRA of these measures immediately, but **no later than 3 days** of the actions being taken.

An amendment is formally submitted as soon as possible by the CI and CTM to the relevant bodies, which will be reviewed and authorised by the Sponsor before submission to HRA and REC.

Examples of issues requiring urgent safety measures may be:

- A single report of a SAR with an unexpected outcome (e.g. death)

- An increase in rate of occurrence of SAR which is judged to be clinically important
- A post-trial SUSAR occurring after the participant has left the trial
- A new event relating to the use or development of an IMP likely to affect the safety of participants e.g.:
    - an SAE that could be associated with the procedures and which could lead to modification of the trial conduct
    - lack of efficacy of an IMP used for a life-threatening disease
    - a major safety finding from a newly completed animal study

14.7 Periodic Safety Reports

Annual progress reports will be submitted to the REC, in accordance with their procedures.

This will be circulated to the study funder and the study sponsor.

14.8 Responsibilities

<u>Principal Investigators (PI):</u>

Checking for AEs and ARs when participants attend for treatment / follow-up.

1. Using medical judgement in assigning seriousness and causality using the Reference Safety Information included in Section 4.8 of the SmPC for each treatment used for the trial.
2. Ensuring that all SAEs and SARs (including SUSARs) are recorded and reported to the Manchester -CTU within 24 hours of becoming aware of the event and provide further follow-up information as soon as available. Ensuring that SAEs and SARs (including SUSARs) are chased with Manchester CTU if a record of receipt is not received within 2 working days of initial reporting.
3. Ensuring that AEs and ARs are recorded and reported to the Manchester CTU in line with the requirements of the protocol.

Chief Investigator (CI) / delegate or independent clinical reviewer:

1. Clinical oversight of the safety of patients participating in the trial, including an ongoing review of the risk / benefit.
2. Using medical judgement in confirming local PI's assessments and/or assigning seriousness and causality of SAEs where it has not been possible to obtain local medical assessment.
3. Using the approved reference safety information to assign expectedness of the event.
4. Review of specific SAEs and SARs in accordance with the protocol
5. Assigning Body System coding to all SAEs and SARs in line with the agreed mechanism stated in the protocol.

Manchester CTU:

1. Central data collection of AEs, ARs, SAEs, SARs and SUSARs according to the trial protocol
2. Reporting safety information to the CI, delegate or independent clinical reviewer for the ongoing assessment of the risk / benefit according to the trial Safety Reporting Procedures.
3. Reporting safety information to the independent oversight committee identified for the trial (Trial Steering Committee (TSC)) according to the Project Delivery Plan.
4. Expedited reporting of SUSARs to the relevant REC within required timelines.
5. Notifying Investigators and the Sponsor of SUSARs that occur within the trial.
6. Checking for (annually) and notifying PIs of updates to the Reference Safety Information for the trial.

Trial Steering Committee (TSC):

In accordance with the Terms of Reference for the TSC, periodically reviewing safety data reports to determine patterns and trends of events, or to identify safety issues, which would not be apparent on an individual case basis.

15 Data Handling

15.1 Data collection tools and source document identification

A paper CRF will be used to collect the data required for the trial. The PI is responsible for ensuring the accuracy, completeness, legibility and timely provision of the data recorded in the CRFs. Only the Investigator and authorised personnel who have signed the Delegation Log provided by the Manchester CTU should record or amend data in the CRFs.

Sites must retain all original reports, traces and images from trial investigations, measures and assessments. Sites should keep sufficient information for all participants to enable records to be linked (e.g. CRFs, hospital records and samples).

Sites will also retain copies of all CRFs submitted to the CTU/Sponsor, including participant completed records to ensure that the principal investigator or research team can provide access to the source documents to a monitor, auditor, or regulatory agency.

15.2 CRFs as Source Documents

Any data recorded directly in the CRF that will not be verifiable from other sources are considered to be source data. [E.g. Participant completed questionnaires].

15.3 Data Handling and Record Keeping

Completed CRFs, submitted to the Manchester CTU at regular intervals, will be reviewed by the designated data manager who will enter the data into the trial database. The database will be secured via appropriate access control and password protection, and subject to regular network backups. Paper records will be stored securely with access limited to authorised personnel.

Data provided to the Manchester CTU will be checked for errors, inconsistencies and omissions. If missing or questionable data are identified, the Manchester CTU will

request that the data be clarified. Pre-defined checking routines will be applied to all batches of data to ensure complete, accurate data are provided for statistical analysis and reporting.

All aspects of data collection and handling throughout the life cycle of the trial will be described in trial specific documents.

15.4 Access to Data

Direct access will be granted to authorised representatives from the Sponsor, host institution and the regulatory authorities to permit trial-related monitoring, audits and inspections.

15.5 Archiving

The TMF and ISF containing essential documents will be archived at an approved external storage facility for a minimum of 25 years. The Sponsor in collaboration with Manchester - CTU will archive the TMF. The Principal Investigator is responsible for the archiving of the ISF at site. Essential documents pertaining to the trial shall not be destroyed without permission from the Sponsor.

Trial sites will be responsible for retaining all source records, including but not limited to, participant medical notes and investigator site files.

16 Trial Monitoring and Management

16.1 Monitoring

Investigators should agree to allow trial related monitoring, including audits and regulatory inspections, by providing direct access to source data/documents as required. Patient consent for this will be obtained.

A Project Delivery Planwill be developed and agreed by the Manchester CTU trial team, including some members of the Trial Management Group (TMG) and TSC. The plan will be based on the trial risk assessment, which may include on site monitoring. The procedures and anticipated frequency for monitoring will be documented in the Project Delivery Plan.

16.1.1 Remote Monitoring

Essential documents will be requested periodically and reviewed remotely by the Clinical Trial Monitor. Details of the documents required and the frequency of the requests will be detailed in the Project Delivery Plan stored at the Manchester CTU.

16.1.2 On-Site Monitoring

On-site monitoring will be defined using a risk-based strategy and a risk assessment will be completed by the Manchester CTU as part of the site set-up process to ascertain the frequency and intensity of monitoring visits required (although additional monitoring may be conducted if necessary). This risk assessment and associated plans to monitor will be stored at the Manchester CTU.

The purpose of these visits is:

- To verify that the rights and well-being of participants are protected.
- To verify accuracy, completion and validity of reported trial data from the source documents.

- To evaluate the conduct of the trial within the institution with regard to compliance with the currently approved protocol, GCP and with the applicable regulatory requirements

16.1.3 Access to data

Direct access will be granted to authorised representatives from the Sponsor and host institution to permit trial-related monitoring, audits and inspections.

16.2 Trial committees and management

The conduct of the trial is being overseen by the following committees:

Trial Management Group (TMG): The TMG will be responsible for the day-to-day running of the trial and will meet quarterly. The TMG members will include the CI, selected co-investigators and identified collaborators, Principal Investigators (PI), , the Clinical Trial Manager,the Trial Statistician, data manager and clinical trial monitor. Notwithstanding the legal obligations of the Sponsor and CI, the TMG have operational responsibility for the conduct of the study including monitoring overall progress to ensure the protocol is adhered to and to take appropriate action to safeguard the participants and the quality of the study.

Trial Steering Committee (TSC): The TSC is the committee that provides overall supervision of the trial in order to safeguard the interests of trial participants. It will consist of an independent Chair, at least one other independent expert in the field of the study, an independent statistician, and a lay representative. The CI will be a non-independent member whilst the Trial Project Manager and Trial Statistician should be invited as observers to all meetings. In addition, and where their expertise may be relevant other research team members, will be invited to attend TSC meetings as observers or non-voting members at the discretion of the chair.

The role of the TSC is to act on behalf of the sponsor, to provide overall supervision for the trial, to ensure that it is conducted in accordance with GCP, and to provide advice to the CI, MCTU, the funder and sponsor on all aspects of the trial through its

independent chairman. The TSC will also cover the remit of an IDMC for this trial, including to recommend after each meeting whether the available accumulated data from the trial, together with results from other relevant trials, justifies continuing recruitment of further participants.

16.3 Public and patient involvement

This protocol and patient related material has been reviewed by a patient representative. Service user will be involved as part of the Trial Steering Committee.

Details of the trial will be made available to the public via the Cancer Research UK clinical trials database.

17 Ethical and regulatory considerations

17.1 Research Ethics Committee Review

Before the start of the trial, the protocol, patient information sheets, consent forms, GP letters and all other associated trial literature will be submitted to a Research Ethics Committee (REC) for review and approval. Confirmation of capacity and capability will also be sought from local Research and Development offices (R&D) before participants are recruited at that site. All amendments to study documentation will require sponsor authorisation prior to submission to HRA and REC for review. All substantial amendments to the trial must be approved by the REC responsible for the study, before the implementation of the amendments. All minor amendments will be notified to the HRA. Any amendments must be approved by the HRA and R&D depts.. before implementation. If the trial is stopped due to adverse events it will not be recommenced without reference to the REC responsible for the study.

The REC will be notified within 90 days of trial completion. If the trial is terminated early, the REC will be notified of this within 15 days. A summary of the clinical trial report will be submitted to the REC responsible for the study within one year of the completion of the last participant's final follow-up procedure. The Chief Investigator will submit an Annual Progress Report to REC on the anniversary date of the study being awarded Favourable Opinion.

17.2 Regulatory compliance

The trial will not commence until a favourable REC opinion is obtained.

The trial procedures are compliant with the Ionising Radiation (Medical Exposure) Regulations, and appropriate review by a Medical Physics Expert and Clinical Radiation Expert has been undertaken.

17.3 Data protection and patient confidentiality

All investigators and trial site staff will comply with the requirements of the Data Protection Act 2018 and General Data Protection Regulation (GDPR) 2018 with regards to the collection, storage, processing and disclosure of personal information and will uphold the Act's core principles.

Participants will be asked to consent to their full name, date of birth, hospital number, postcode and NHS number being collected prior to randomisation. This is to allow tracing through GP and NHS Digital to assist with follow up data. All personal data will be regarded as confidential and any information which would identify individual patients will not be released into the public domain.

At randomisation, patients will be allocated a unique trial identifying number, which will then be used on all trial documentation, such as CRFs. A separate log of trial IDs, patient names etc. will be kept in a locked filing cabinet at each site.

All electronic records will be kept secure by using encrypted digital files within password protected folders on NHS computers for any clinical data and the Manchester Cancer Research Centre computers for any laboratory data. Data pertaining to the laboratory results will be held on folder at the Manchester Cancer Research Centre (MCRC) which conforms to GCP standards and which has entry permissions to individuals involved in the trial and restricted read only access.

Access to patient information will be limited to the minimum number of individuals necessary for quality control, audit, and analysis.

Confidentiality of data will be preserved when the data are transmitted to sponsors and co-investigators by the use of sponsor approved, password protected, and digitally encrypted files.

The Chief Investigator has overall responsibility for the data collected as part of this trial.

17.4 Protocol compliance

Protocol deviations, non-compliances, or breaches are departures from the approved protocol.

Prospective, planned deviations or waivers to the protocol are not allowed under the Clinical Trials Regulations and must not be used e.g. it is not acceptable to enrol a patient if they do not meet the eligibility criteria or restrictions specified in the trial protocol.

Accidental protocol deviations can happen at any time. They must be adequately documented on the relevant forms and reported to the Trial Manager immediately.

Deviations from the protocol which are found to frequently recur are not acceptable, will require immediate action and could potentially be classified as a serious breach.

17.5 Notification of Serious Breaches to GCP and/or the protocol

A "serious breach" is a breach of GCP or the protocol which is likely to effect to a significant degree –

(a) the safety or physical or mental integrity of the participants of the trial; or

(b) the scientific value of the trial/ data reliability or robustness

The Manchester CTU or Sponsor will notify the REC in writing of any serious breach of the protocol or GCP.

17.6 Indemnity

The design of the research and Protocol are covered by the University of Manchester indemnity as Professor Lorigan's substantive employer. The trial sites and investigators will be covered by the NHS indemnity scheme.

18 Publication Policy

Upon completion of the trial the MCTU and CI will prepare a clinical study report based on the final data set.

In keeping with the guidance for the Research Governance Framework, the information arising from the study will be made available to the study population that it affects, the clinical community who may use the information and also to anyone who may benefit from the study's findings. In order to achieve this, the study will be submitted for presentation at scientific and clinical meetings, publication in peer-reviewed periodicals and a lay summary of results will be publicly available via website such as CancerHelp which is hosted by CRUK.

The TMG will form the basis of the writing committee and will advise on the nature of presentation and publications, subject to funder/sponsor requirements. All presentations and publications relating to the study must be authorised by the TMG, Sponsor and study drug manufacturer and must acknowledge the funders, supporting bodies (participating sites associated NHS trusts) and the Sponsor. . Individual participating PIs may not publish data concerning their participants that are directly relevant to questions posed by the trial until the submission of the clinical study report or first peer-review publication, whichever is the latter

19 References

1. Atkins MB, Larkin J. Immunotherapy Combined or Sequenced With Targeted Therapy in the Treatment of Solid Tumors : Current Perspectives. 2016;108:1–9.
2. Merlino G, Herlyn M, Fisher DE, Bastian BC, Flaherty KT, Davies MA, et al. The state of melanoma: challenges and opportunities. Pigment Cell Melanoma Res [Internet]. 2016; Available from: http://doi.wiley.com/10.1111/pcmr.12475
3. Luke JJ, Flaherty KT, Ribas A, Long G V. Targeted agents and immunotherapies: optimizing outcomes in melanoma. Nat Rev Clin Oncol [Internet]. 2017 Apr 4 [cited 2017 Jul 29];14(8):463–82. Available from: http://www.ncbi.nlm.nih.gov/pubmed/28374786
4. Song G, Gu L, Li J, Tang Z, Liu H, Chen B, et al. Serum microRNA expression profiling predict response to R-CHOP treatment in diffuse large B cell lymphoma patients. Ann Hematol [Internet]. 2014 Oct [cited 2014 Dec 14];93(10):1735–43. Available from: http://www.ncbi.nlm.nih.gov/pubmed/24858372
5. Schadendorf D, et al. Pooled analysis of long-term survival data from phase II and phase III trials of ipilimumab in metastatic or locally advanced, unresectable melanoma. ESMO Annu Meet [Internet]. 2013;Abstract 24. Available from: http://eccamsterdam2013.ecco-org.eu/Scientific-Programme/Abstract-search.aspx#
6. Robert C, Karaszewska B, Schachter J, Rutkowski P, Mackiewicz A, Stroiakovski D, et al. Improved Overall Survival in Melanoma with Combined Dabrafenib and Trametinib. N Engl J Med [Internet]. 2015;372(1):30–9. Available from: http://www.nejm.org/doi/10.1056/NEJMoa1412690
7. Ugurel S, Röhmel J, Ascierto PA, Flaherty KT, Grob JJ, Hauschild A, et al. Survival of patients with advanced metastatic melanoma: The impact of novel therapies. Eur J Cancer. 2016;53:125–34.
8. Spartalizumab plus dabrafenib and trametinib (Sparta-DabTram) in patients (pts) with previously untreated BRAF V600–mutant unresectable or metastat... | OncologyPRO [Internet]. [cited 2020 Oct 22]. Available from: https://oncologypro.esmo.org/meeting-resources/esmo-virtual-congress-2020/spartalizumab-plus-dabrafenib-and-trametinib-sparta-dabtram-in-

patients-pts-with-previously-untreated-braf-v600-mutant-unresectable-or-metastat
9. Gutzmer R, Stroyakovskiy D, Gogas H, Robert C, Lewis K, Protsenko S, et al. Atezolizumab, vemurafenib, and cobimetinib as first-line treatment for unresectable advanced BRAFV600 mutation-positive melanoma (IMspire150): primary analysis of the randomised, double-blind, placebo-controlled, phase 3 trial. Lancet [Internet]. 2020 Jun 13 [cited 2020 Jun 17];395(10240):1835–44. Available from: https://linkinghub.elsevier.com/retrieve/pii/S014067362030934X
10. First report of efficacy and safety from the phase II study SECOMBIT (SEquential COMBo Immuno and Targeted therapy study) | OncologyPRO [Internet]. [cited 2020 Dec 8]. Available from: https://oncologypro.esmo.org/meeting-resources/esmo-virtual-congress-2020/first-report-of-efficacy-and-safety-from-the-phase-ii-study-secombit-sequential-combo-immuno-and-targeted-therapy-study
11. Immunotherapy With Ipilimumab and Nivolumab Preceded or Not by a Targeted Therapy With Encorafenib and Binimetinib - Full Text View - ClinicalTrials.gov [Internet]. [cited 2020 Dec 8]. Available from: https://clinicaltrials.gov/ct2/show/NCT03235245
12. Liu C, Peng W, Xu C, Lou Y, Zhang M, Wargo JA, et al. BRAF inhibition increases tumor infiltration by T cells and enhances the antitumor activity of adoptive immunotherapy in mice. Clin Cancer Res. 2013;19(2):393–403.
13. Hu-Lieskovan S, Mok S, Homet Moreno B, Tsoi J, Robert L, Goedert L, et al. Improved antitumor activity of immunotherapy with BRAF and MEK inhibitors in BRAFV600E melanoma. Sci Transl Med [Internet]. 2015 Mar 18;7(279):279ra41-279ra41. Available from: http://stm.sciencemag.org/content/7/279/279ra41.abstract
14. Boni A, Cogdill AP, Dang P, Udayakumar D, Njauw CNJ, Sloss CM, et al. Selective BRAFV600E inhibition enhances T-cell recognition of melanoma without affecting lymphocyte function. Cancer Res. 2010;70(13):5213–9.
15. Khalili JS, Liu S, Rodr??guez-Cruz TG, Whittington M, Wardell S, Liu C, et al. Oncogenic BRAF(V600E) promotes stromal cell-mediated immunosuppression via induction of interleukin-1 in melanoma. Clin Cancer Res. 2012;18(19):5329–40.

16. Frederick DT, Piris A, Cogdill AP, Cooper ZA, Lezcano C, Ferrone CR, et al. BRAF inhibition is associated with enhanced melanoma antigen expression and a more favorable tumor microenvironment in patients with metastatic melanoma. Clin Cancer Res [Internet]. 2013 Mar 1 [cited 2017 Apr 10];19(5):1225–31. Available from: http://www.ncbi.nlm.nih.gov/pubmed/23307859
17. Tumeh PC, Harview CL, Yearley JH, Shintaku IP, Taylor EJM, Robert L, et al. PD-1 blockade induces responses by inhibiting adaptive immune resistance. Nature [Internet]. 2014 Nov 26 [cited 2014 Nov 26];515(7528):568–71. Available from: http://dx.doi.org/10.1038/nature13954
18. Jiang X, Zhou J, Giobbie-Hurder A, Wargo J, Hodi FS. The activation of MAPK in melanoma cells resistant to BRAF inhibition promotes PD-L1 expression that is reversible by MEK and PI3K inhibition. Clin Cancer Res. 2013;19(3):598–609.
19. Ott PA, Henry T, Baranda SJ, Frleta D, Manches O, Bogunovic D, et al. Inhibition of both BRAF and MEK in BRAFV600E mutant melanoma restores compromised dendritic cell (DC) function while having differential direct effects on DC properties. Cancer Immunol Immunother [Internet]. 2013;62(4):811–22. Available from: http://dx.doi.org/10.1007/s00262-012-1389-z
20. Wang T, Xiao M, Ge Y, Krepler C, Belser E, Lopez-Coral A, et al. BRAF Inhibition Stimulates Melanoma-Associated Macrophages to Drive Tumor Growth. Clin Cancer Res [Internet]. 2015 [cited 2017 Apr 10];21(7). Available from: http://clincancerres.aacrjournals.org/content/21/7/1652.long
21. Hugo W, Shi H, Sun L, Piva M, Song C, Kong X, et al. Non-genomic and Immune Evolution of Melanoma Acquiring MAPKi Resistance. Cell [Internet]. 2015 Sep 10 [cited 2016 Jul 12];162(6):1271–85. Available from: http://www.ncbi.nlm.nih.gov/pubmed/26359985
22. Larkin J, Chiarion-Sileni V, Gonzalez R, Grob JJ, Cowey CL, Lao CD, et al. Combined Nivolumab and Ipilimumab or Monotherapy in Untreated Melanoma. N Engl J Med [Internet]. 2015 May 31 [cited 2015 Jun 2];373:23–34. Available from: http://www.ncbi.nlm.nih.gov/pubmed/26027431
23. Hodi FS, O'Day SJ, McDermott DF, Weber RW, Sosman J a, Haanen JB, et al. Improved survival with ipilimumab in patients with metastatic melanoma. N Engl J Med [Internet]. 2010 Aug 19;363(8):711–23. Available from: http://www.ncbi.nlm.nih.gov/pubmed/23439861
24. Robert C, Long G V., Brady B, Dutriaux C, Maio M, Mortier L, et al. Nivolumab

in Previously Untreated Melanoma without BRAF Mutation. N Engl J Med [Internet]. 2014 Nov 16 [cited 2014 Nov 16];141116004513004. Available from: http://www.ncbi.nlm.nih.gov/pubmed/25399552

25. Diem S, Kasenda B, Spain L, Martin-Liberal J, Marconcini R, Gore M, et al. Serum lactate dehydrogenase as an early marker for outcome in patients treated with anti-PD-1 therapy in metastatic melanoma. Br J Cancer [Internet]. 2016 Feb 2 [cited 2017 Apr 10];114(3):256–61. Available from: http://www.ncbi.nlm.nih.gov/pubmed/26794281

26. Blank CU, Haanen JB, Ribas A, Schumacher TN. CANCER IMMUNOLOGY. The "cancer immunogram". Science [Internet]. 2016 May 6 [cited 2016 Jul 24];352(6286):658–60. Available from: http://www.ncbi.nlm.nih.gov/pubmed/27151852

27. Pilon-Thomas S, Kodumudi KN, El-Kenawi AE, Russell S, Weber AM, Luddy K, et al. Neutralization of Tumor Acidity Improves Antitumor Responses to Immunotherapy. Cancer Res [Internet]. 2016 Mar 15 [cited 2017 Apr 10];76(6):1381–90. Available from: http://www.ncbi.nlm.nih.gov/pubmed/26719539

28. Brand A, Singer K, Koehl GE, Kolitzus M, Schoenhammer G, Thiel A, et al. LDHA-Associated Lactic Acid Production Blunts Tumor Immunosurveillance by T and NK Cells. Cell Metab [Internet]. 2016 [cited 2017 Apr 10];24(5):657–71. Available from: http://www.sciencedirect.com/science/article/pii/S1550413116304272

29. Das Thakur M, Salangsang F, Landman AS, Sellers WR, Pryer NK, Levesque MP, et al. Modelling vemurafenib resistance in melanoma reveals a strategy to forestall drug resistance. Nature. 2013 Feb;494(7436):251–5.

30. Moriceau G, Hugo W, Hong A, Shi H, Kong X, Yu CC, et al. Tunable-combinatorial mechanisms of acquired resistance limit the efficacy of BRAF/MEK cotargeting but result in melanoma drug addiction. Cancer Cell [Internet]. 2015 Feb 9 [cited 2016 Sep 27];27(2):240–56. Available from: http://www.ncbi.nlm.nih.gov/pubmed/25600339

31. Girotti MR, Gremel G, Lee R, Galvani E, Rothwell D, Viros A, et al. Application of sequencing, liquid biopsies, and patient-derived xenografts for personalized medicine in melanoma. Cancer Discov [Internet]. 2016; Available from: http://www.scopus.com/inward/record.url?eid=2-s2.0-

84960075353&partnerID=MN8TOARS

32. Santiago-Walker A, Gagnon R, Mazumdar J, Casey M, Long G V., Schadendorf D, et al. Correlation of BRAF Mutation Status in Circulating-Free DNA and Tumor and Association with Clinical Outcome across Four BRAFi and MEKi Clinical Trials. Clin Cancer Res. 2016;22(3).

33. Shinozaki M, O'Day SJ, Kitago M, Amersi F, Kuo C, Kim J, et al. Utility of circulating B-RAF DNA mutation in serum for monitoring melanoma patients receiving biochemotherapy. Clin Cancer Res [Internet]. 2007 Apr 1 [cited 2015 Jan 10];13(7):2068–74. Available from: http://www.pubmedcentral.nih.gov/articlerender.fcgi?artid=2720029&tool=pmcentrez&rendertype=abstract

34. Hindson BJ, Ness KD, Masquelier DA, Belgrader P, Heredia NJ, Makarewicz AJ, et al. High-Throughput Droplet Digital PCR System for Absolute Quantitation of DNA Copy Number. 2011 Nov 15 [cited 2017 Feb 19];83(22):8604–10. Available from: https://pubs.acs.org/doi/pdf/10.1021/ac202028g

35. Long G V., Stroyakovskiy D, Gogas H, Levchenko E, de Braud F, Larkin J, et al. Combined BRAF and MEK Inhibition versus BRAF Inhibition Alone in Melanoma. N Engl J Med [Internet]. 2014 Nov 13 [cited 2018 Jul 28];371(20):1877–88. Available from: http://www.nejm.org/doi/10.1056/NEJMoa1406037

36. Santiago-Walker A, Gagnon R, Mazumdar J, Casey M, Long G V., Schadendorf D, et al. Correlation of BRAF mutation status in circulating-free DNA and tumor and association with clinical outcome across four BRAFi and MEKi clinical trials. Clin Cancer Res [Internet]. 2015;1–9. Available from: http://clincancerres.aacrjournals.org/cgi/doi/10.1158/1078-0432.CCR-15-0321

20 Appendices

Appendix A. Response criteria (Recist v1.1)

For full RECIST version 1.1 criteria please refer to:

https://ctep.cancer.gov/protocoldevelopment/docs/recist_guideline.pdf

For participants who have measurable disease at baseline, Table 4 provides a summary of the overall response status calculation at each time point.

Table 4 Time Point Response - Participants With Target (Non-target) Disease

Target lesions	Non-Target lesions	New lesions	Overall response
CR	CR	No	CR
CR	Non-CR/non-PD	No	PR
CR	Not evaluated	No	PR
PR	Non-PD or not all evaluated	No	PR
SD	Non-PD or not all evaluated	No	SD
Not all evaluated	Non-PD	No	NE
PD	Any	Yes or No	PD
Any	PD	Yes or No	PD
Any	Any	Yes	PD

CR = Complete Response, PR = Partial Response, SD = Stable Disease, PD = Progressive Disease, NE = Not Evaluable

Missing Assessments and Not Evaluable Designation

When no imaging/measurement is done at all at a particular time point, the participant is not evaluable (NE) at that time point. If only a subset of lesion measurements are made at an assessment, the case is also considered NE at that time point, unless a convincing argument can be made that the contribution of the individual missing lesion(s) would not have changed the assigned time-point response.

Appendix B. ECOG performance status

Table 5 ECOG performance status

Grade	ECOG status
0	Fully active, able to carry on all pre-disease performance without restriction
1	Restricted in physically strenuous activity but ambulatory and able to carry out work of a light or sedentary nature, e.g., light house work, office work
2	Ambulatory and capable of all selfcare but unable to carry out any work activities. Up and about more than 50% of waking hours
3	Capable of only limited selfcare, confined to bed or chair more than 50% of waking hours
4	Completely disabled. Cannot carry on any selfcare. Totally confined to bed or chair
5	Dead

Appendix C. Cockcroft Gault Formula and Wright Formula

Cockroft Gault Formula

C_{Cr}={((140–age) x weight)/(72xS_{Cr})} x 0.85 (if female)

Wright Formula

GFR={(6550-(38.8 x age)) x 0.832 x BSA}/SCr (if female)

Abbreviations/ Units

C_{Cr} (creatinine clearance) = mL/minute

Age = years

Weight = kg

SCr (serum creatinine) = mg/dL

GFR (glomerular filtration rate) = ml/min

BSA (body surface area) = Du Bois formula (0.007184 x weight$^{0.425}$ x height$^{0.725}$)

Appendix D. WOCBP and male participants sexually active with WOCBP

Definition of WOCBP

Defined as any female who has experienced menarche and who has not undergone surgical sterilization (hysterectomy or bilateral oophorectomy) and is not post-menopausal. Menopause is defined as 12 months of amenorrhea in a woman over age 45 years in the absence of other biological or physiological causes. In addition, females under the age of 55 years must have a serum follicle stimulating hormone, (FSH) level > 40mIU/mL to confirm menopause).

Females treated with hormone replacement therapy (HRT) are likely to have artificially suppressed FSH levels and may require a washout period in order to obtain a physiologic FSH level. The duration of the washout period is a function of the type of HRT used. The duration of the washout period below are suggested guidelines and the investigators should use their judgement in checking serum FSH levels. If the serum FSH level is >40 mIU/mL at any time during the washout period, the woman can be considered postmenopausal:

- 1 week minimum for vaginal hormonal products (rings, creams, gels)
- 4 week minimum for transdermal products
- 8 week minimum for oral products
- Other parenteral products may require washout periods as long as 6 months.

Management of WOCBP and male participants sexually active with WOCBP

Investigators shall counsel WOCBP and male participants who are sexually active with WOCBP on the importance of pregnancy prevention and the implications of an unexpected pregnancy. Investigators shall advise WOCBP and male participants who are sexually active with WOCBP on the use of highly effective methods of contraception. Highly effective methods of contraception have a failure rate of < 1% when used consistently and correctly.

At a minimum, participants must agree to the use of two methods of contraception, with one method being highly effective and the other method being either highly effective or less effective as listed below:

HIGHLY EFFECTIVE METHODS OF CONTRACEPTION

Hormonal methods of contraception including:

combined oral contraceptive pills,

vaginal ring,

injectables,

implants and intrauterine devices (IUDs) such as Mirena® by WOCBP participants or male participant's WOCBP partner. Female partners of male participants in the study may use hormone-based contraceptives as one of the acceptable methods of contraception since they will not be receiving study drug.

Nonhormonal IUDs, such as ParaGard®

Tubal ligation

Vasectomy

Complete abstinence

NOTE: Complete abstinence is defined as complete avoidance of heterosexual intercourse and is an acceptable form of contraception for all study drugs. Participants who choose complete abstinence are not required to use a second method of contraception, but female participants must continue to have pregnancy tests.

Acceptable alternate methods of highly effective contraception must be discussed in the event that the participant chooses to forego complete abstinence.

LESS EFFECTIVE METHODS OF CONTRACEPTION

Diaphragm with spermicide

Cervical cap with spermicide

Vaginal sponge

Male condom without spermicide

Progestin only pills by WOCBP participant or male participant's WOCBP partner

Female condom

Note: A male and female condom must not be used together

Appendix E. Medications specifically prohibited on dabrafenib and trametinib

- Dabrafenib is metabolized primarily by Cytochrome P450 (CYP) 2C8 and CYP3A4. Drugs that are strong inhibitors or inducers of CYP3A or CYP2C8 (see list in Table 7) may increase or decrease, respectively, dabrafenib concentrations and therapeutic substitutions for these medications should be undertaken.
- Drugs that are strong inhibitors or inducers of CYP3A and CYP2C8 (see list in Table 7) may only be used under special circumstances (e.g. as a single use for a procedure) while treatment with study drug is interrupted as they may alter dabrafenib concentrations; consider therapeutic substitutions for these medications. Approval from the CI is required in these situations. These include but are not limited to those listed in Table 7.

Table 7 Prohibited Medications on dabrafenib and trametinib

**Strong inducers of CYP3A or CYP2C8, since concentrations of dabrafenib may be decreased**	
**Class/Therapeutic Area**	**Drugs/Agents**
Antibiotics	Rifamycin class agents (e.g., rifampin, rifabutin, rifapentine),
Anticonvulsant	Carbamazepine, oxcarbazepine phenobarbital, phenytoin, s-mephenytoin
Miscellaneous	bosentan, St. John's wort
**Strong inhibitors of CYP3A, or CYP2C8 since concentrations of dabrafenib may be increased**	
**Class/Therapeutic Area**	**Drugs/Agents**
Antibiotics	Clarithromycin, telithromycin, troleandomycin
Antidepressant	Nefazodone
Antifungals	Itraconazole, ketoconazole, posaconazole, voriconazole
Hyperlipidemia	Gemfibrozil
Antiretroviral	ritonavir, saquinavir, atazanavir
Miscellaneous	Conivaptan

Medications to be used with caution on dabrafenib and trametinib:

The following medications should be used with caution as their concentrations may be altered by dabrafenib or they may alter dabrafenib concentrations:

- Drugs that are moderate inhibitors or inducers of CYP3A and CYP2C8 as they may alter concentrations of dabrafenib.
- Dabrafenib has been shown to induce CYP3A4 and CYP2C9 in vivo using midazaolam (CYP3A4 substrate) and S-warfarin (CYP2C9 substrate). Dabrafenib is an in vitro inducer CYP2B6 and other enzymes such as CYP2C8, CYP2C19, UDP-glucuronyl transferases. Transporters may also be affected. Co-administration of dabrafenib and medications which are affected by the induction of these enzymes (including warfarin) and transporters may result in loss of efficacy. If co-administration of these medications is necessary, investigators should monitor subjects for loss of efficacy or consider substitutions of these medications. A partial list of these medications is provided in Table 8.
- Therapeutic level dosing of warfarin can be used as long as close monitoring of PT/INR is performed by the site. Warfarin exposure has been shown to decrease (37% decrease) due to dabrafenib-mediated enzyme induction. Conversely, if dabrafenib dosing is reduced, interrupted, or discontinued, warfarin exposure may be increased. Thus, warfarin dosing may need to be adjusted based on PT/INR during and after treatment with dabrafenib. Prophylactic low dose warfarin may be given to maintain central catheter patency.
- Dabrafenib solubility is pH-dependent with decreased solubility at higher pH. Drugs such as proton pump inhibitors that inhibit gastric acid secretion to elevate gastric pH may decrease the solubility of dabrafenib and reduce its bioavailability therefore should be used with caution.

Table 8 Medications to be used with caution on dabrafenib and trametinib

**Moderate inhibitors of CYP3A, or CYP2C8 since concentrations of dabrafenib may be increased**	
**Class/Therapeutic Area**	**Drug**
Antiarrhythmics	Diltiazem, verapamil
Antibiotic	Erythromycin
Antifungal	Fluconazole
Miscellaneous	Aprepitant
**Co-administration of these drugs with study treatment may result in loss of efficacy. Monitor subjects for loss of efficacy or substitute with another medication.**	
**Class/Therapeutic Area**	**Drug**
Analgesics	Alfentanil, buprenorphine, celecoxib, codeine, fentanyl, methadone, oxycodone
Antiarrhythmics	Disopyramide, dronedarone, mexiletine, propafenone, quinidine
Antibiotics	Chloramphenicol, doxycycline, erythromycin, moxifloxacin
Anticoagulants/ Antiplatelets	Cilostazole, warfarin
Anticonvulsants	Divalproex, lamotrigine, valproate, zonisamide
Antidepressants and Antipsychotics	Aripiprazole, bupropion, buspirone, desipramine, haloperidol, mirtazapine, pimozide, quetiapine, trazodone, amitriptyline, clomipramine, imipramine
Antidiabetics	Glyburide, saxagliptin, tolbutamide, nateglinide, pioglitazone, repaglinide, rosiglitazone
Antifungals	Caspofungin, fluconazole, terbinafine
Antihistamines	Astemizole, chlorpheniramine, ebastine
Antihypertensives	Amlodipine, diltiazem, felodipine, nifedipine, nilvadipine, nisoldipine, verapamil
Antimigraine Agents	Diergotamine, eletriptan, ergotamine
Corticosteroids	Dexamethasone, methylprednisolone, oral budesonide
Erectile Dysfunction Agents	Sildenafil, tadalafil, vardenafil
HMG-CoA Reductase Inhibitors	Atorvastatin, lovastatin, simvastatin, rosuvastatin, pravastatin
Hypnotics and Sedatives	Alprazolam, brotizolam, diazepam, estazolam, midazolam, triazolam, zolpidem, zopiclone
Immunosuppressants	Everolimus, sirolimus, tacrolimus
Miscellaneous	Aprepitant, cisapride, darifenacin, digoxin, disopyramide, leflunomide, methohexital, oral contraceptives, quinine, ranitidine, solifenacin, sulfasalazine, tramadol, tolvaptan, chloroquine,
Selective Aldosterone Blockers	Eplerenone
**Co-administration of drugs that increase gastric pH should be used with caution**	
pH altering agents	dexlansoprazole. esomeprazole, famotidine, ilaprazole, lansoprazole, omeprazole, pantoprazole, rabeprazole, ranitidine

Abbreviations: ; CYP = cytochrome P450;
HMG-CoA = 3-hydroxy-3-methylglutaryl-coenzyme A;

Appendix F. Management of immune related adverse events

Below are standardised algorithms for the management of immune related AEs. Further guidance can be obtained from the CI or the CTM.

It is important to identify immune-relates AEs early in order to initiate treatment as soon as possible, which results in decreased morbidity to the patient. In addition, differential diagnoses should be diligently evaluated according to standard medical practice. Non-inflammatory etiologies should be considered and appropriately treated.

Corticosteroids are a primary therapy for immuno-oncology drug-related adverse events. The oral equivalent of the recommended IV doses may be considered for ambulatory patients with low-grade toxicity. The lower bioavailability of oral corticosteroids should be taken into account when switching to the equivalent dose of oral corticosteroids.

Consultation with a medical or surgical specialist, especially prior to an invasive diagnostic or therapeutic procedure, is recommended.

Neurological Adverse Event Management Algorithm

Rule out non-inflammatory causes. If non-inflammatory cause, treat accordingly and continue I-O therapy.

Grade of Neurological Toxicity (NCI CTCAE v4)	Management	Follow-up
**Grade 1**<br>Asymptomatic or mild symptoms; Intervention not indicated	• Continue I-O therapy per protocol	Continue to monitor the patient.<br>If worsens:<br>• Treat as Grade 2 or 3-4
**Grade 2**<br>Moderate symptoms; Limiting instrumental ADL	• Delay I-O therapy per protocol<br>• Treat symptoms per local guidelines<br>• Consider 0.5 to 1.0 mg/kg/day methylprednisolone IV or PO equivalent	If improves to baseline:<br>•Resume I-O therapy per protocol when improved to baseline<br>If worsens:<br>• Treat as Grade 3-4
**Grade 3-4**<br>Severe symptoms; Limiting self-care ADL; Life-threatening	• Discontinue I-O therapy per protocol<br>• Obtain neurology consult<br>• Treat symptoms per local guidelines<br>• 1.0-2.0 mg/kg/day IV methylprednisolone IV or IV equivalent<br>• Add prophylactic antibiotics for opportunistic infections	If improves to Grade 2:<br>• Taper steroids over at least 1 month<br><br>If worsens or atypical presentation:<br>• Consider IVIG or other immunosuppressive therapies per local guidelines

Patients on IV steroids may be switched to an equivalent dose of oral corticosteroids (e.g. prednisone) at start of tapering or earlier, once sustained clinical improvement is observed. Lower bioavailability of oral corticosteroids should be taken into account when switching to the equivalent dose of oral corticosteroids.

Endocrinopathy Management Algorithm

Rule out non-inflammatory causes. If non-inflammatory cause, treat accordingly and continue I-O therapy. Consider visual field testing, endocrinology consultation, and imaging.

Asymptomatic thyroid stimulating hormone (TSH) elevation

- Continue I-O therapy per protocol
- If TSH < 0.5 x lower limit of normal (LLN), or TSH > 2 x ULN, or consistently out of range in 2 subsequent measurements: include free thyroxine (fT4) at subsequent cycles as clinically indicated; consider endocrinology consult

Symptomatic endocrinopathy

- Evaluate endocrine function
- Consider pituitary scan

Symptomatic with abnormal lab/pituitary scan:
- Delay I-O therapy per protocol
- 1-2 mg/kg/day methylprednisolone IV or by mouth (PO) equivalent
- Initiate appropriate hormone therapy

No abnormal lab/pituitary MRI scan but symptoms persist:
- Repeat labs in 1-3 weeks /MRI in 1 month

If improves (with or without hormone replacement):
- Taper steroids over at least 1 month and consider prophylactic antibiotics for opportunistic infections
- Resume I-O therapy per protocol
- Patients with adrenal insufficiency may need to continue steroids with mineralocorticoid component

Suspicion of adrenal crisis (e.g. severe dehydration, hypotension, shock out of proportion to current illness

- Delay or discontinue I-O therapy per protocol
- Rule out sepsis
- Stress dose of IV steroids with mineralocorticoid activity
- IV fluids
- Consult endocrinologist
- If adrenal crisis ruled out, then treat as above for symptomatic endocrinopathy

Patients on IV steroids may be switched to an equivalent dose of oral corticosteroids (e.g. prednisone) at start of tapering or earlier, once sustained clinical improvement is observed. Lower bioavailability of oral corticosteroids should be taken into account when switching to the equivalent dose of oral corticosteroids.

GI Adverse Event Management Algorithm

Rule out non-inflammatory causes. If non-inflammatory cause is identified, treat accordingly and continue I-O therapy. Opiates/narcotics may mask symptoms of perforation. Infliximab should not be used in cases of perforation or sepsis.

Grade of Diarrhea/ Colitis (NCI CTCAE v4)	Management	Follow-up
**Grade 1**<br>Diarrhea: < 4 stools/day over baseline;<br>Colitis: asymptomatic	• Continue I-O therapy per protocol<br>• Symptomatic treatment	• Close monitoring for worsening symptoms.<br>• Educate patient to report worsening immediately<br>If worsens:<br>• Treat as Grade (G) 2 or 3/4
**Grade 2**<br>Diarrhea: 4-6 stools per day over baseline; IV fluids indicated <24 hours (hrs); not interfering with ADL<br>Colitis: abdominal pain; blood in stool	• Delay I-O therapy per protocol<br>• Symptomatic treatment	If improves to grade 1:<br>• Resume I-O therapy per protocol<br>If persists > 5-7 days or recur:<br>• 0.5-1.0 mg/kg/day methylprednisolone or oral equivalent<br>• When symptoms improve to grade 1, taper steroids over at least 1 month, consider prophylactic antibiotics for opportunistic infections, and resume I-O therapy per protocol.<br>If worsens or persists > 3-5 days with oral steroids:<br>• Treat as grade 3/4
**Grade 3-4**<br>Diarrhea (G3): ≥7 stools per day over baseline; incontinence; IV fluids ≥24 hrs; interfering with activities of daily living (ADL)<br>Colitis (G3): severe abdominal pain, medical intervention indicated, peritoneal signs<br>G4: life-threatening, perforation	• Discontinue I-O therapy per protocol<br>• 1.0 to 2.0 mg/kg/day methylprednisolone IV or IV equivalent<br>• Add prophylactic antibiotics for opportunistic infections<br>• Consider lower endoscopy	If improves:<br>• Continue steroids until grade 1, then taper over at least 1 month<br>If persists > 3-5 days, or recurs after improvement:<br>• Add infliximab 5 mg/kg (if no contraindication). Note: Infliximab should not be used in cases of perforation or sepsis

Patients on IV steroids may be switched to an equivalent dose of oral corticosteroids (e.g. prednisone) at start of tapering or earlier, once sustained clinical improvement is observed. Lower bioavailability of oral corticosteroids should be taken into account when switching to the equivalent dose of oral corticosteroids.

Renal Adverse Event Management Algorithm

Rule out non-inflammatory causes. If non-inflammatory cause, treat accordingly and continue I-O therapy

Grade of Creatinine Elevation (NCI CTCAE v4)	Management	Follow-up
**Grade 1**<br>Creatinine > upper limit of normal (ULN) and > than baseline but ≤ 1.5x baseline	• Continue I-O therapy per protocol<br>• Monitor creatinine weekly	If returns to baseline:<br>•Resume routine creatinine monitoring per protocol<br>If worsens:<br>•Treat as Grade 2 or 3/4
**Grade 2-3**<br>Creatinine > 1.5x baseline to ≤ 6x ULN	• Delay I-O therapy per protocol<br>• Monitor creatinine every 2-3 days<br>• 0.5 to 1.0 mg/kg/day methylprednisolone IV or oral equivalent<br>• Consider renal biopsy	If returns to Grade 1:<br>•Taper steroids over at least 1 month, consider prophylactic antibiotics for opportunistic infections, and resume I-O therapy and routine creatinine monitoring per protocol<br><br>If elevations persist > 7 days or worsen:<br>•Treat as Grade 4
**Grade 4**<br>Creatinine > 6x ULN	• Discontinue I-O therapy per protocol<br>• Monitor creatinine daily<br>• 1.0-2.0 mg/kg/day methylprednisolone IV or IV equivalent<br>• Consult nephrologist<br>• Consider renal biopsy	If returns to Grade 1 : Taper steroids over at least 1 month and add prophylactic antibiotics for opportunistic infections

Hepatic Adverse Event Management Algorithm

Rule out non-inflammatory causes. If non-inflammatory cause, treat accordingly and continue I-O therapy. Consider imaging for obstruction.

Grade of Liver Test Elevation (NCI CTCAE v4)	Management	Follow-up
**Grade 1**<br>AST or ALT > ULN to 3.0 x ULN <u>and/or</u> Total bilirubin (T. bili) > ULN - 1.5 x ULN	• Continue I-O therapy per protocol	• Continue liver function tests (LFT) monitoring per protocol<br><u>If worsens:</u><br>• Treat as Grade 2 or 3-4
**Grade 2**<br>AST or ALT > 3.0 to ≤ 5 x ULN <u>and/or</u> T. bili > 1.5 to ≤ 3 x ULN	• Delay I-O therapy per protocol<br>• Increase frequency of monitoring to every 3 days	<u>If returns to baseline</u>:<br>•Resume routine monitoring, resume I-O therapy per protocol<br><br><u>If elevations persist > 5-7 days or worsen :</u><br>• 0.5-1 mg/kg/day methylprednisolone or oral equivalent and when LFT returns to grade 1 or baseline, taper steroids over at least 1 month, consider prophylactic antibiotics for opportunistic infections, and resume I-O therapy per protocol
**Grade 3-4**<br>AST or ALT > 5 x ULN <u>and /or</u> T.bili >3 x ULN	• Discontinue I-O therapy*<br>• Increase frequency of monitoring to every 1-2 days<br>• 1.0 to 2.0 mg/kg/day methylprednisolone IV or IV equivalent**<br>• Add prophylactic antibiotics for opportunistic infections<br>• Consult gastroenterologist	<u>If returns to grade 2</u>:<br>• Taper steroids over at least 1 month<br><br><u>If does not improve in >3-5 days, worsens or rebounds:</u><br>• Add mycophenolate mofetil 1 gram (g) twice daily (BID)<br>• If no response within an additional 3-5 days, consider other immunosuppressants per local guidelines

Patients on IV steroids may be switched to an equivalent dose of oral corticosteroids (e.g. prednisone) at start of tapering or earlier, once sustained clinical improvement is observed. Lower bioavailability of oral corticosteroids should be taken into account when switching to the equivalent dose of oral corticosteroids.

***I-O therapy may be delayed rather than discontinued if AST/ALT ≤ 8 x ULN and T.bili ≤ 5 x ULN.**

**The recommended starting dose for grade 4 hepatitis is 2 mg/kg/day methylprednisolone IV.

Skin Adverse Event Management Algorithm

Rule out non-inflammatory causes. If non-inflammatory cause, treat accordingly and continue I-O therapy.

Grade of Rash (NCI CTCAE v4)	Management	Follow-up
**Grade 1-2**<br>Covering ≤ 30% body surface area (BSA)*	• Symptomatic therapy (e.g. antihistamines, topical steroids)<br>• Continue I-O therapy per protocol	<u>If persists > 1-2 weeks or recurs:</u><br>• Consider skin biopsy<br>• Delay I-O therapy per protocol<br>• Consider 0.5-1.0 mg/kg/day methylprednisolone IV or oral equivalent. Once improving , taper steroids over at least 1 month, consider prophylactic antibiotics for opportunistic infections, and resume I-O therapy per protocol<br><u>If worsens:</u><br>• Treat as Grade 3-4
**Grade 3-4**<br>Covering >30% BSA; Life threatening consequences*	• Delay or discontinue I-O therapy per protocol<br>• Consider skin biopsy<br>• Dermatology consult<br>• 1.0-2.0 mg/kg/day IV methylprednisolone IV or IV equivalent	<u>If improves to Grade 1:</u><br>• Taper steroids over at least 1 month and add prophylactic antibiotics for opportunistic infections<br>• Resume I-O therapy per protocol

Patients on IV steroids may be switched to an equivalent dose of oral corticosteroids (e.g. prednisone) at start of tapering or earlier, once sustained clinical improvement is observed. Lower bioavailability of oral corticosteroids should be taken into account when switching to the equivalent dose of oral corticosteroids.
*Refer to NCI CTCAE v4 for term-specific grading criteria.

Pulmonary Adverse Event Management Algorithm

Rule out non-inflammatory causes. If non-inflammatory cause, treat accordingly and continue I-O therapy. Evaluate with imaging and pulmonary consultation.

Grade of Pneumonitis (NCI CTCAE v4)	Management	Follow-up
**Grade 1**<br>Radiographic changes only	• Consider delay of I-O therapy<br>• Monitor for symptoms every 2-3 days<br>• Consider Pulmonary and Infectious Disease (ID) consults	• Re-image at least every 3 weeks<br>If worsens:<br>• Treat as Grade 2 or 3-4
**Grade 2**<br>Mild to moderate new symptoms	• Delay I-O therapy per protocol<br>• Pulmonary and ID consults<br>• Monitor symptoms daily, consider hospitalization<br>• 1.0 mg/kg/day methyl-prednisolone IV or oral equivalent<br>• Consider bronchoscopy, lung biopsy	• Re-image every 1-3 days<br>If improves:<br>• When symptoms return to near baseline, taper steroids over at least 1 month and then resume I-O therapy per protocol and consider prophylactic antibiotics<br>If not improving after 2 weeks or worsening:<br>• Treat as Grade 3-4
**Grade 3-4**<br>Severe new symptoms; New/worsening hypoxia; Life-threatening	• Discontinue I-O therapy per protocol<br>• Hospitalize<br>• Pulmonary and ID consults<br>• 2-4 mg/kg/day methylprednisolone IV or IV equivalent<br>• Add prophylactic antibiotics for opportunistic infections<br>• Consider bronchoscopy, lung biopsy	If improves to baseline:<br>• Taper steroids over at least 6 weeks<br>If not improving after 48 hours or worsening:<br>• Add additional immunosuppression (e.g. infliximab, cyclophosphamide, intravenous immunoglobulin (IVIG), or mycophenolate mofetil)

Patients on IV steroids may be switched to an equivalent dose of oral corticosteroids (e.g. prednisone) at start of tapering or earlier, once sustained clinical improvement is observed. Lower bioavailability of oral corticosteroids should be taken into account when switching to the equivalent dose of oral corticosteroids.

CRUK Manchester Institute
Clinical and Experimental Pharmacology

**Project Code**	**CEP-0359**
**Short Title**	Validation of ddPCR BRAF V600 Assay

Project Report

Project Long Title:

Validation of droplet digital PCR (ddPCR) BRAF V600 assay for the CAcTUS trial

Test Facility

Clinical and Experimental Pharmacology Group
Cancer Research UK Manchester Institute
The University of Manchester, Alderley Park
Macclesfield, SK10 4TG, United Kingdom

**Author**	**Owner**
Dominic Rothwell	Nigel Smith

A record of all approvers, revision history and active date is maintained within Q-Pulse and can be accessed from the document record.

CRUK Manchester Institute
Clinical and Experimental Pharmacology

**Project Code**	**CEP-0359**
**Short Title**	Validation of ddPCR BRAF V600 Assay

1. Project Overview

**Project Type**	Clinical Trial/ Experimental Medicine	☐	Equipment Evaluation	☐	Method Development/ Validation	☒
	Other	☐	Specify:			
**Validation Level** (If Applicable)	Proof of principle	☐	Fit for purpose	☐	Fully Qualified	☒
	3° Endpoint	☐	2° Endpoint	☐	1° Endpoint	☒
**Validation Outcome** (If Applicable)	Successful	☒	Unsuccessful	☐		
**Report Type**	Interim	☐	Final	☒		

2. Project and Personnel Details

**Date of first experiment**	22 January 2018
**Date of last experiment**	16 March 2018
**Project Deadline Date** **(Date work MUST be completed by to facilitate other work or sample analysis)**	May 2018
**Project Manager**	Nigel Smith
**Deputy Project Manager**	Mahmood Ayub
**Analyst(s)**	Nigel Smith, Mahmood Ayub
**QA Representative**	Tony Price

Project Code	CEP-0359
Short Title	Validation of ddPCR BRAF V600 Assay

3. Glossary

%VAF	Percentage Variant Allele Frequency
cfDNA	Circulating Free DNA
ctDNA	Circulating Tumour DNA
ddPCR	droplet digital Polymerase Chain Reaction
DNA	Deoxyribonucleic acid
FAM	Carboxyfluorescein (Absorption 494nm, Emission 518nm)
HEX	Hexachlorofluorescein (Absorption 530nm, Emission 556nm)
HNV	Healthy Normal Volunteer
IQ	Installation Qualification
LLOD	Lower Limit of Detection
LLOQ	Lower Limit of Quantitation
LOB	Limit of Blank
MBCF	Molecular Biology Core Facility
NTC	No Template Control
OQ	Operational Qualification
PQ	Performance Qualification
VAF	Variant Allele Frequency
WT	Wild Type
Generated Droplets	Refers to the total droplets generated by the droplet generator prior to thermal cycling and analysis
Signal Positive Droplets	Refers to the number of droplets measured on the droplet reader that contain amplified material (Mutant and wild type)
Signal negative Droplets	Refers to the number of droplets measured on the droplet reader that contain no amplified material ('empty' droplets)
Mutant positive droplets	Refers to the number of droplets measured on the droplet reader that contain mutant positive signal
Wild Type positive droplets	Refers to the number of droplets measured on the droplet reader that contain wild type positive signal

4. Introduction

This project was intended to validate the detection of three common BRAF V600 mutations (V600E, K & R) at low frequency in ctDNA derived from cancer patient plasma for use in the CAcTUS trial. Analysis of ctDNA from patient plasma using ddPCR will allow the real time quantitation of the variant allele frequency (VAF; the frequency of a variant allele in relationship to the germ-line allele) of specific mutations giving the opportunity for the data to be used to make clinical decisions for the benefit of the patient (See appendix 1 for the sponsors clinical assessment of the risks and benefits to the patient in using the data from this assay). In line with the clinical protocol a reduction of ≥80% in

Project Code	CEP-0359
Short Title	Validation of ddPCR BRAF V600 Assay

the mutant VAF of a time point sample relative to the baseline value will trigger change of treatment. Each of the BRAF V600 mutations was detected by specific singleplex assays that were validated individually as part of this programme of work. In addition, experiments with a three-plex assay performed to allow for future expansion and development of the ddPCR assay. Each assay also contained WT control primers to enable quantitation of both the mutant and WT copies in the sample, allowing the measurement of the VAF.

The scope of the validation was to assess the ddPCR assay of cfDNA and control DNA as suitable for use in the CAcTUS trial. The CAcTUS clinical protocol designated this assay as a primary endpoint due to the fact that the data influences the scheduling the switch of patient treatment. Therefore the assay has been validated for use in primary endpoint analysis.

The methods by which the DNA was isolated were out of scope of the validation.

5. Overview of ddPCR technology

Droplet Digital PCR technology is a digital PCR method utilising a water-oil emulsion droplet system. Droplets are formed in a water-oil emulsion to form the partitions that separate the template DNA molecules. In traditional PCR, a single sample offers only a single measurement, but in ddPCR, the sample is partitioned into 20,000 nanoliter-sized droplets. This partitioning enables the measurement of thousands of independent amplification events within a single sample.

Bio-Rad's QX200 ddPCR System consists of two instruments, the QX200 Droplet Generator and the QX200 Droplet Reader, plus their associated software and consumables. The QX200 Droplet Generator partitions samples (20 µl into 20,000 nanoliter-sized droplets) for PCR amplification. Following amplification using a thermal cycler, droplets from each sample are analysed individually on the QX200 Droplet Reader, where PCR-positive and PCR-negative droplets are counted to provide absolute quantification of target DNA in digital form.

The Process can be divided in 5 steps:

- Step 1: Prepare PCR-Ready Samples prior to Starting ddPCR
- Step 2: Droplet Generation
- Step 3: PCR Amplification of Droplets
- Step 4: Droplet Reading
- Step 5: Analyse Results

Positive droplets, containing at least one copy of the target, exhibit increased fluorescence over negative droplets. In ddPCR, the QuantaSoft software

**Project Code**	**CEP-0359**
**Short Title**	Validation of ddPCR BRAF V600 Assay

measures the numbers of droplets that are positive and negative for each fluorophore (FAM and HEX) in a sample.

To calculate %VAF, two targets are PCR amplified in the same reaction. One is for mutant template (FAM fluorescence) and the other for wild type template (HEX fluorescence). These are plotted on a 2-D plot and the droplets are clustered into four quadrants as shown in **Figure 1**.

Figure 1: 2-D Scatter plot of ddPCR analysis

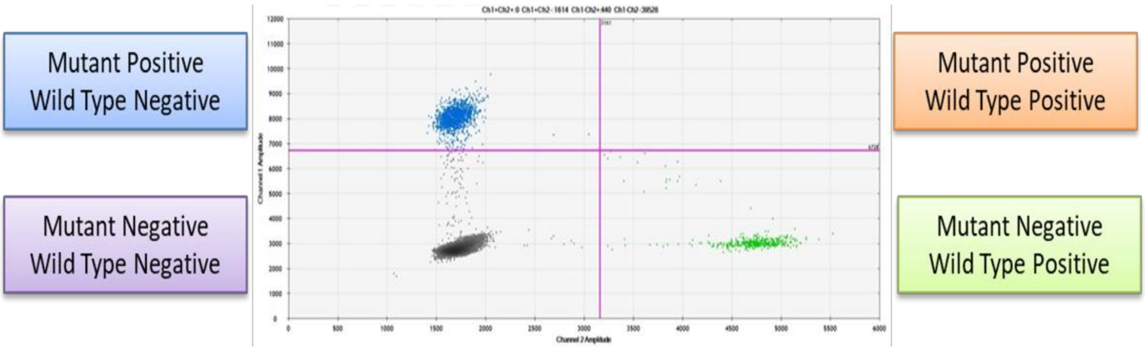

The data from the 4 quadrants is exported and analysed in a bespoke document controlled Excel Spread Sheet and the %VAF [(number of mutant positive droplets / number of signal positive droplets) x 100] reported.

Positive droplets, which contain at least one copy of the target DNA molecule, exhibit increased fluorescence compared to negative droplets (Refer to pages 4-5 of the Droplet Digital PCR applications guide). Combined with the high specificity of the primers, samples with a single mutant positive droplet can be confidently reported as a positive result however, the accuracy at which a %VAF is determined is reliant upon the total number of signal positive droplets.

6. Objectives

To verify that the ddPCR assay can accurately and reproducibly detect VAF levels ≥5% when ≥1ng of DNA is added to the assay, reducing to ≥1% when ≥5ng of DNA is used. We also aimed to demonstrate that each assay was specific for an individual mutation and had a false positive rate <0.2%.

To demonstrate that each ddPCR assay performs within specified acceptance criteria in the following areas:

1) Specificity, sensitivity and reproducibility of ddPCR assays
2) The LOB
3) The LLOD and LLOQ
4) Reproducibility and repeatability of assay QCs

Project Code	CEP-0359
Short Title	Validation of ddPCR BRAF V600 Assay

5) Quantification of VAF in clinical samples
6) IQ/OQ/PQ of the ddPCR equipment

7. Materials and Methods

Table 1. Equipment used for validation

Equipment	Catalogue No.	Serial Number	Reference
QX200 Automated Droplet Generator	1864002	773BR1672	EQUIP-367
QX200 Droplet Reader	1864003	773BR2983	EQUIP-370
C1000 Touch Thermal Cycler with 96-deep well reaction module	1851197	CT025660	EQUIP-369
PX1 PCR Plate sealer	1814000	770BR3615	EQUIP-368

The list of equipment in **Table 1** is assay specific; Pipettes and centrifuges used in the validation were recorded within laboratory notebook CN/782. All equipment was qualified and within servicing and calibration at the time of use.

Table 2. Reagents and consumables used for validation

Item	Supplier	Catalogue Number	Lot Number	Legacy Chemlog Reference	LIMS Chemlog Reference (s)
ddPCR Supermix for Probes (No dUTP)	Bio-Rad	186-3024	64130190	AP0007	CHEM007036
20x ddPCR assay (primer-Probe mix) for BRAF p.V600E c.1799T>A	Bio-Rad	10049550 Assay ID: dHsaMDV2010027	351951	AP0031	CHEM007060
20x ddPCR assay (primer-Probe mix) for BRAF p.V600K c.1798_1799GT>AA	Bio-Rad	10049550 Assay ID: dHsaMDV2010035	349193	AP0030	CHEM007059
20x ddPCR assay (primer-Probe mix) for BRAF p.V600R c.1798_1799GT>AG	Bio-Rad	10049550 Assay ID: dHsaMDV2010037	351968	AP0032	CHEM007061
Auto DG Droplet Generation Oil for Probes	Bio-Rad	186-4110	64086320<br>64110150	AP0005<br>AP0040	CHEM007034<br>CHEM007069
ddPCR™ Droplet Reader Oil	Bio-Rad	1863004	64086321<br>64109506	AP0006<br>AP0039	CHEM007035<br>CHEM007068
2x ddPCR Buffer Control for Probes	Bio-Rad	1863052	64037633	AP0038	CHEM007067
Nuclease Free Water	Qiagen	157014451	129114	Not Applicable	CHEM005658
Low EDTA TE	Invitrogen	12092-015	1851100	Not Applicable	CHEM005582
DG32 Automated Droplet Generator	Bio-Rad	1864108	It is assumed that these consumables are not lot dependant and therefore the		

Project Code	CEP-0359
Short Title	Validation of ddPCR BRAF V600 Assay

Item	Supplier	Catalogue Number	Lot Number	Legacy Chemlog Reference	LIMS Chemlog Reference (s)
cartridges			lot numbers were not recorded in this validation.		
PCR Plate Heat Seal, foil, pierceable	Bio-Rad	1814040			
ddPCR 96-Well Plates	Bio-Rad	12001925			

Table 3. Related documents

Reference	Title
PLN-51	Validation of ddPCR BRAF V600 Assay
SOP-77	Isolation of cfDNA using the QIAamp circulating nucleic acid kit
FRM-66	Isolation of cfDNA from plasma or serum samples using a QIAamp circulating nucleic acid kit
SOP-27	Extraction of DNA using the QIAamp Blood Mini Kit
FRM-62	Extraction of DNA using the QIAamp Blood Mini Kit
SOP-44	Use and Maintenance of Nanodrop Spectrophotometers
MISC-16	Nanodrop 2000 Plate Planner
MISC-19	Bioruptor Guide
RSP-10	Agarose Gel Electrophoresis
FRM-92	Agarose Gel Electrophoresis
SOP-82	RNase P qPCR Quantitation Assay
FRM-68	RNase P qPCR RNase P qPCR Quantitation Assay
MISC-14	RNase P qPCR Quantitation Assay
Copy in project folder (GCLP Drive:\Projects\CEP359\Project Related Documents\Manuals)	Droplet Digital PCR applications guide (Bulletin 6407 RevA)
Copy in project folder (GCLP Drive:\Projects\CEP359\Project Related Documents\Manuals)	ddPCR Mutation Detection Assays, Validated Instruction Manual (Version: 10033487 Ver C)
Copy in project folder (GCLP Drive:\Projects\CEP359\Project Related Documents\Manuals)	Automated Droplet Generator Instruction Manual (QX200) (Version: 10043138 RevB)
Copy in project folder (GCLP Drive:\Projects\CEP359\Project Related Documents\Manuals)	QX200 Droplet reader and Quantasoft software instruction manual (Version: 10031906 Rev D)
Copy in project folder (GCLP Drive:\CEP359\LIMS Linked Documents	Bio bank approval letter for patient control samples: Mechanisms of resistance to vemurafenib in metastatic melanoma (13_RIMA_01)
SOP-105	Use and Maintenance of the Bio-Rad QX200 Auto DG Droplet Digital PCR System
RSP-52	Determination of Variant Allele Frequency using Droplet Digital PCR
FRM-160	Determination of Variant Allele Frequency using Droplet Digital PCR
MISC-98	Determination of Variant Allele Frequency using Droplet Digital PCR

Project Code	CEP-0359
Short Title	Validation of ddPCR BRAF V600 Assay

8. Deviations from the Validation Plan

For ease of reference a summary of the deviations from the validation plan is given in **Table 4**. These deviations are detailed and discussed in the relevant parts of the Experimental Results Analysis section (**Section 9**).

Table 4. Summary of the deviations

Deviation / Comment	Applicable Section(s) In the Plan	Applicable Section(s) In the report
Sections of the plan refer to a %VAF±X% or <X%. This variation was interpreted as a percentage of %VAF rather than '%VAF±X' (I.E. define a range of %VAF-X to %VAF+X).	2.1<br>2.4<br>2.6	9.1<br>9.4<br>9.6
Some sections of the plan could be interpreted slightly differently than was intended. Therefore these sections have been re-phrased to clarify the original intention and avoid ambiguity.	2.1<br>2.2<br>2.4	9.1<br>9.2<br>9.4
An assumption that the theoretical numbers of copies would match the measured number of droplets in the assay was found to be incorrect. This section of the plan has been re-evaluated based on this information.	2.3	9.3

9. Experimental Results Analysis

Note: References below tables and figures refer to the filename of the source data and the location within that file. These are stored in the validation project folder as described in ***section 11.***

9.1. Specificity, reproducibility and repeatability of ddPCR Assays

This refers to section 2.1 of the validation plan

Aim: The aim is to demonstrate the specificity, reproducibility and repeatability of each set of primers in individual and plexed ddPCR reactions.

Project Code	CEP-0359
Short Title	Validation of ddPCR BRAF V600 Assay

Acceptance Criteria:

Table 5. Specificity acceptance criteria of %VAF of the controls measured in each ddPCR assay

	%VAF Acceptance Criteria			
	BRAF V600E Control (A375)	BRAF V600K Control (RM44)	BRAF V600R Control (M381)	BRAF V600WT Control (RM59)
BRAF V600E ddPCR	>90%	<1%	<1%	<1%
BRAF V600K ddPCR	<1%	>75%, <85%	<1%	<1%
BRAF V600R ddPCR	<1%	<1%	>28%, <38%	<1%
BRAF V600plex ddPCR	>90%	>75%, <85%	>28%, <38%	<2%

Specificity will be demonstrated by positive results seen for each control cell-line DNA in their respective ddPCR's in line with the acceptance levels shown above.
Reproducibility will be considered acceptable if the intra-assay CV (variation between wells in the same experiment) is <5%.
Repeatability will be considered acceptable if the inter-assay CV (variation between experiments) is <5%.

Deviation from plan:

During the validation it was identified that the acceptance criteria for Reproducibility and Repeatability was incorrectly described as <5% rather than a %VAF ±5. To demonstrate specificity, the acceptance criteria shown in **Table 5** was a range of 10 (equivalent to an expected value ±5 or >90 for values between 90-100 %VAF) for positive controls. To fulfil this, the reproducibility and repeatability acceptance criteria for positive controls would be ±5 of their %VAF (%VAF of the determined inter-assay QC shown in **Table 7B**). For the purposes of transparency the results have been compared to both sets of acceptance criteria and are shown below in **Tables 7A & 7B**.

Similarly, reproducibility and repeatability acceptance criteria for negative controls would be < 1 %VAF, based on the specificity acceptance of <1 %VAF and are shown below in **Table 6**.

Results:

The mean %VAF of the control DNA was measured in three independent experiments to demonstrate specificity, reproducibility and repeatability.
Related experiments: 359782009, 359782010 & 359782011

Project Code	CEP-0359
Short Title	Validation of ddPCR BRAF V600 Assay

Table 6. Summary of specificity results

ddPCR Assay	Control Cell Line	A375	RM44	M381	RM59
	Mutation Status	(BRAF V600E)	(BRAF V600K)	(BRAF V600R)	(BRAF V600WT)
BRAF V600E	Determined %VAF	100.00	0.00	0.00	0.00
	SD %VAF	0.00	0.00	0.00	0.00
	Acceptance Range	>90%	<1%	<1%	<1%
	Outcome	Pass	Pass	Pass	Pass
BRAF V600K	Determined %VAF	0.00	78.77	0.00	0.00
	SD %VAF	0.00	0.54	0.00	0.00
	Acceptance Range	<1%	>75%, <85%	<1%	<1%
	Outcome	Pass	Pass	Pass	Pass
BRAF V600R	Determined %VAF	0.00	0.00	33.48	0.00
	SD %VAF	0.00	0.00	1.76	0.00
	Acceptance Range	<1%	<1%	>28%, <38%	<1%
	Outcome	Pass	Pass	Pass	Pass
BRAF V600plex (E + K +R)	Determined %VAF	100.00	78.39	32.15	0.00
	SD %VAF	0.00	0.85	1.78	0.00
	Acceptance Range	>90%	>75%, <85%	>28%, <38%	<2%
	Outcome	Pass	Pass	Pass	Pass

(Table location: '2.1 Figures' Tab. Filename: 'Validation data 2.1')

Table 7a. Summary of reproducibility and repeatability results with acceptance criteria from the plan

Sample	ddPCR Assay		Experiment Number			Inter-assay %VAF
			359782009	359782010	359782011	
A375 (BRAF V600E)	BRAF V600E	Determined %VAF	100.00	100.00	100.00	100.00
		Standard Deviation %VAF	0.00	0.00	0.00	0.00
		%CV	0.00	0.00	0.00	0.00
		Acceptance Criteria (%CV)	<5%	<5%	<5%	<5%
		Outcome	Pass	Pass	Pass	Pass
RM44 (BRAF V600K)	BRAF V600K	Determined %VAF	78.50	79.39	78.43	78.77
		Standard Deviation %VAF	2.29	1.55	0.45	0.54
		%CV	2.92	1.95	0.57	0.69
		Acceptance Criteria (%CV)	<5%	<5%	<5%	<5%
		Outcome	Pass	Pass	Pass	Pass
M381 (BRAF V600R)	BRAF V600R	Determined %VAF	33.51	31.70	35.22	33.48
		Standard Deviation %VAF	2.99	0.78	0.46	1.76
		%CV	8.92	2.46	1.31	5.26
		Acceptance Criteria (%CV)	<5%	<5%	<5%	<5%
		Outcome	FAIL	Pass	Pass	Pass
A375 (BRAF V600E)	BRAF V600plex (E + K +R)	Determined %VAF	100.00	100.00	100.00	100.00
		Standard Deviation %VAF	0.00	0.00	0.00	0.00
		%CV	0.00	0.00	0.00	0.00
		Acceptance Criteria (%CV)	<5%	<5%	<5%	<5%
		Outcome	Pass	Pass	Pass	Pass
RM44 (BRAF V600K)	BRAF V600plex (E + K +R)	Determined %VAF	78.10	79.35	77.72	78.39
		Standard Deviation %VAF	0.63	1.69	1.57	0.85
		%CV	0.81	2.13	2.02	1.09
		Acceptance Criteria (%CV)	<5%	<5%	<5%	<5%
		Outcome	Pass	Pass	Pass	Pass
M381 (BRAF V600R)	BRAF V600plex (E + K +R)	Determined %VAF	32.97	33.37	30.11	32.15
		Standard Deviation %VAF	3.13	2.88	2.42	1.78
		%CV	9.49	8.64	8.03	5.54
		Acceptance Criteria (%CV)	<5%	<5%	<5%	<5%
		Outcome	FAIL	FAIL	FAIL	FAIL

(Table location: '2.1 Figures' Tab. Filename: 'Validation data 2.1')

Project Code	CEP-0359
Short Title	Validation of ddPCR BRAF V600 Assay

Table 7b. Summary of reproducibility and repeatability results with the clarified acceptance criteria

Sample	ddPCR Assay		Experiment Number			Inter-assay %VAF
			359782009	359782010	359782011	
A375 (BRAF V600E)	BRAF V600E	Determined %VAF	100.00	100.00	100.00	100.00
		Measured Range	100.00 to 100.00	100.00 to 100.00	100.00 to 100.00	100.00 to 100.00
		Acceptance Range*	95.00 - 100.00	95.00 - 100.00	95.00 - 100.00	95.00 - 100.00
		Outcome	Pass	Pass	Pass	Pass
RM44 (BRAF V600K)	BRAF V600K	Determined %VAF	78.50	79.39	78.43	78.77
		Measured Range	75.89 to 80.21	77.67 to 80.66	77.91 to 78.72	78.43 to 79.38
		Acceptance Range*	73.77 - 83.77	73.77 - 83.77	73.77 - 83.77	73.77 - 83.77
		Outcome	Pass	Pass	Pass	Pass
M381 (BRAF V600R)	BRAF V600R	Determined %VAF	33.51	31.70	35.22	33.48
		Measured Range	30.06 to 35.26	30.84 to 32.36	34.73 to 35.64	31.70 to 35.22
		Acceptance Range*	28.48 - 38.48	28.48 - 38.48	28.48 - 38.48	28.48 - 38.48
		Outcome	Pass	Pass	Pass	Pass
A375 (BRAF V600E)	BRAF V600plex (E + K +R)	Determined %VAF	100.00	100.00	100.00	100.00
		Measured Range	100.00 to 100.00	100.00 to 100.00	100.00 to 100.00	100.00 to 100.00
		Acceptance Range*	95.00 - 100.00	95.00 - 100.00	95.00 - 100.00	95.00 - 100.00
		Outcome	Pass	Pass	Pass	Pass
RM44 (BRAF V600K)	BRAF V600plex (E + K +R)	Determined %VAF	78.10	79.35	77.72	78.39
		Measured Range	77.45 to 78.72	77.84 to 81.18	76.24 to 79.36	77.72 to 79.35
		Acceptance Range*	73.77 - 83.77	73.77 - 83.77	73.77 - 83.77	73.77 - 83.77
		Outcome	Pass	Pass	Pass	Pass
M381 (BRAF V600R)	BRAF V600plex (E + K +R)	Determined %VAF	32.97	33.37	30.11	32.15
		Measured Range	29.37 to 35	30.38 to 36.13	27.38 to 31.97	30.11 to 33.37
		Acceptance Range*	28.48 - 38.48	28.48 - 38.48	28.48 - 38.48	28.48 - 38.48
		Outcome	Pass	Pass	Pass	Pass
*Acceptance Range is ±5 of the determined inter-assay %VAF for that QC / Assay						

(Table location: '2.1 Figures' Tab. Filename: 'Validation data 2.1')

- All the positive controls for each assay met their specificity acceptance criteria. The %VAF for the Wild type and negative controls was 0.00% in all assays, meeting the acceptance criteria of <1% (**Table 6**).
- The reproducibility acceptance criteria of %VAF values between replicate wells in the same experiment being within ±5 of the determined %VAF was met in all assays **(Table 7b)**.
- The repeatability acceptance criteria of the %VAF values between experiments being within ±5 of the determined %VAF was met in all assays **(Table 7b)**.
- The reproducibility (inter-assay CV) acceptance criteria of <5% as mistakenly set in the validation plan was met in 8 out of 9 experiments for single plex assays and 6 out of 9 experiments for multiplex assays **(Table 7a)**.
- The repeatability (intra-assay CV) acceptance criteria of <5% as mistakenly set in the validation plan was met in all 3 singleplex assays and 2 out of 3 multiplex assays **(Table 7a)**.

Project Code	CEP-0359
Short Title	Validation of ddPCR BRAF V600 Assay

Conclusions:

Each assay is specific to their respective mutation with no evidence of cross reactivity seen in the single plex or multiplex assays.
The data shows that the Inter-assay CV and Intra-assay CV increases at lower %VAF as expected, resulting in one single plex assay failing to meet its acceptance criteria at 33% VAF. The use of the '±5' as the acceptance criteria better reflects the technology and type of analysis, considering the mechanics of population analysis and each discrete droplet containing a high level of fluorescence (See **Section 5**). The standard deviations determined from the singleplex assays indicate a variation within ±3 of the determined %VAF, well within the ±5 acceptance criteria. The implications are discussed in further detail in **Section 10.1**.
The data from the multiplex assay indicates the variation may be raised compared to single plex assays and further work would be required to draw specific conclusions regarding the suitability of the multiplex assay for use in routine analysis.

9.2. Limit of Blank (LOB)

This refers to section 2.2 of the validation plan

Aim: To demonstrate the LOB for the assay which is the maximum number of positive droplets detected in a negative control sample.

Acceptance Criteria:

(i) LOB for a NTC / Blank: ≤2 positive droplets per 1515 molecules (<0.2%)
(ii) LOB for a negative control sample: ≤2 positive droplets per 1515 molecules (<0.2%)
(iii) LOB for a negative HNV sample: ≤2 positive droplets per 1515 molecules (<0.2%)

Deviation from Plan:

It was not the intention to determine the LOB, but to demonstrate that it does not exceed acceptable levels outlined in the acceptance criteria.
The plan states '≤2 positive droplets/1515 molecules (0.2%)' in (i), (ii) & (iii). This has been re-phrased to clarify the original intention and avoid ambiguity in the report.

(i) LOB for a NTC / Blank: ≤2 signal positive droplets per 1515 signal negative droplets
(ii) LOB for a negative control sample: ≤2 mutant positive droplets per 1515 signal positive droplets

Project Code	CEP-0359
Short Title	Validation of ddPCR BRAF V600 Assay

(iii) LOB for a negative HNV sample: ≤2 mutant positive droplets per 1515 signal positive droplets

Given that each individual droplet contains a high fluorescent signal, even 1 signal positive droplet can be detected leads to the conclusion that ≤1 per 757 is equivalent to ≤2 per 1515 (See **Section 5** for justification).

Results:

(i) LOB for NTC

The LOB for a blank was demonstrated by including a no template control (NTC) in 5 validation experiments.
Related experiments:
359782019, 359782020, 359782023, 359782024 & 359782025

Table 8. Summary of LOB NTC results

		BRAF V600 E		BRAF V600 K		BRAF V600 R	
Experiment Number	Experiment Number	Total Number of signal Positive Droplets	Total Number of signal Negative Droplets	Total Number of signal Positive Droplets	Total Number of signal Negative Droplets	Total Number of signal Positive Droplets	Total Number of signal Negative Droplets
359782019	Well 1	0	8367	0	9752	0	10435
	Well 2	0	7660	0	8293	0	9601
359782020	Well 1	0	12143	0	15545	0	12920
	Well 2	0	13571	0	15364	0	13062
359782023	Well 1	0	15963	0	13871	0	14888
	Well 2	0	15721	0	15003	0	15865
359782024	Well 1	No Data*		0	11705	0	12123
	Well 2			0	12780	0	11121
359782025	Well 1	0	15561	0	15771	0	15888
	Well 2	0	15071	0	15882	0	15139
359782022*	Well 1	0	9140	N/A	N/A	N/A	N/A
	Well 2	0	11907				
	Outcome	Pass	Pass	Pass	Pass	Pass	Pass

*The 'No Data' Result was a technical failure with those wells and did not compromise the assay validity. The samples were repeated in experiment 359782022 (wells H01 & H02) to acheive 5 sets of data in accordance with the plan.

(Table location: 'LOB(i)_LOB(ii) Figures' Tab. Filename: 'Validation data 2.4')

No signal positive droplets were measured in any of the 30 NTC reaction wells. This was in a background of between 7,660 and 15,963 signal negative droplets, with a mean value of 13,004 **(Table 8)**. This demonstrates the acceptance criteria of ≤2 signal positive droplets per 1515 signal negative droplets for the LOB for NTC was met.

(ii) LOB for negative controls

The LOB for a negative control sample was demonstrated using a cell line derived wild type BRAF DNA in 5 validation experiments.
Related experiments:
359782019, 359782020, 359782023, 359782024 & 359782025

Table 9. Summary of negative control results

	BRAF V600 E		BRAF V600 K		BRAF V600 R	
Experiment Number	Total Number of mutant Positive Droplets	Total Number of signal positive Droplets	Total Number of mutant Positive Droplets	Total Number of signal positive Droplets	Total Number of mutant Positive Droplets	Total Number of signal positive Droplets
359782019	0	1039	0	981	0	1274
359782020	0	1786	0	1939	0	1762
359782023	0	1681	0	1826	0	1859
359782024	0	1483	0	1700	0	1163
359782025	0	1788	0	1635	0	1892
Outcome	Pass	Pass	Pass	Pass	Pass	Pass

(Table location: 'LOB(i)_LOB(ii) Figures' Tab. Filename: 'Validation data 2.4')

The wild type control samples gave no mutant positive droplets for all 3 single plex assays from all 5 experiments (**Table 9**). 10 of the 15 results gave no mutant positive droplets in >1515 wild type positive droplets (≤2 per 1515) and 5 of the 15 results gave no mutant positive droplets in >757 but <1515 wild type positive droplets (≤1 per 757). This demonstrates the acceptance criteria of ≤2 mutant positive droplets per 1515 signal positive droplets for LOB for negative control samples was met.

(iii) LOB for HNV samples

The LOB for HNV control samples was demonstrated using cfDNA isolated from 3 HNV donors in duplicate validation experiments.
Related experiments: 359782031 & 359782032

Table 10. Summary of LOB for a negative HNV sample results

Assay Details		Experiment: 359782031				Experiment: 359782032			
Sample	Assay	Total Number of Mutant Positive Droplets	Total Number of Signal Positive Droplets	%VAF	≤2 mutant positive droplets per 1515 signal positive droplets Pass / Fail	Total Number of Mutant Positive Droplets	Total Number of Signal Positive Droplets	%VAF	≤2 mutant positive droplets per 1515 signal positive droplets Pass / Fail
359001	V600 E	0	1065	0.00	Pass*	0	1094	0.00	Pass*
359002	V600 E	0	2045	0.00	Pass	0	2211	0.00	Pass
359003	V600 E	0	1721	0.00	Pass	0	1845	0.00	Pass
359001	V600 K	0	909	0.00	Pass*	0	1099	0.00	Pass*
359002	V600 K	0	1833	0.00	Pass	0	2603	0.00	Pass
359003	V600 K	0	1959	0.00	Pass	0	2039	0.00	Pass
359001	V600 R	0	989	0.00	Pass*	0	1376	0.00	Pass*
359002	V600 R	0	780	0.00	Pass*	0	1286	0.00	Pass*
359003	V600 R	0	2113	0.00	Pass	0	2474	0.00	Pass
359003	Plex	0	2160	0.00	Pass	0	2504	0.00	Pass
*These samples pass based on ≤1 mutant positive droplets per 757 signal positive droplets									

(Table location: '2.2 Figures' Tab. Filename: 'Validation data 2.2')

Project Code	CEP-0359
Short Title	Validation of ddPCR BRAF V600 Assay

cfDNA from 3 HNV donors gave no mutant positive droplets for all 3 single plex assays in 2 experiments **(Table 10)**. 10 of the 18 results gave no mutant positive droplets in >1515 signal positive droplets (≤2 per 1515) and 8 of the 18 results gave no mutant positive droplets in >757 but <1515 signal positive droplets (≤1 per 757).

2 out of 2 results using the plex assay also gave no mutant positive droplets in >1515 signal positive droplets (≤2 per 1515).

This data demonstrates the acceptance criteria of ≤2 mutant positive droplets per 1515 signal positive droplets for LOB with HNV donors was met.

Conclusions:

No false positive droplets were observed in all validation experiments, meeting the acceptance criteria and leading to the conclusion that the assays have a very low false positive rate (**Table 11**).

Table 11. Maximum False Positive Rate

	Number of false positive droplets	Total number of droplets	% False Positives
NTC	≤1	>7660	<0.013
Negative Controls	≤1	>757	<0.13
HNV cfDNA	≤1	>757	<0.13

The data supports the assertion that samples which gives 3 or more mutant positive droplets gives a valid positive result.

The use of an NTC in each assay is not necessary providing a suitable negative control sample is used.

9.3. Lower Limit of Detection (LLOD) and Quantification (LLOQ)

This refers to section 2.3 of the validation plan

Aim: The VAF LLOD and LLOQ will differ with varying amounts of template DNA input (Table 2, [in the plan]). Therefore, a LLOD of ≥3 mutant positive drops and LLOQ of 10 (approximately x3 above LLOD) mutant positive drops will be set for the assay.

Table 12. Theoretical VAF LLOD and LLOQ values, figures based on 1 ng DNA = 303 genome copies

		5.0 ng input	2.0 ng input	1.0 ng input	0.5 ng input
	Theoretical total number of positive droplets	1515	606	303	151
LLOD	Minimum number of mutant positive droplets	3	3	3	3
	VAF %	0.2	0.5	1.0	2.0
LLOQ	Minimum number of mutant positive droplets	10	10	10	10
	VAF %	0.66	1.65	3.3	6.6

Acceptance Criteria:

The justification for these values is described in section 3 [in the plan]. The VAF LLOD and LLOQ will vary according to sample input (**Table 12**). The acceptance criteria for the LLOD and LLOQ for each input level will be ±10% of expected VAF or ±3 positive droplets at lower VAF frequencies:

Table 13. Acceptance criteria for LLOD and LLOQ for ddPCR assays

		Expected Variant Allele Frequency (VAF)							
	Sample Input	20%	10%	5%	1%	0.50%	0.25%	0%	NTC
LLOD	5ng	20% ±2%	10% ±1%	5% ±0.5%	1% ±0.2%	0.5% ±0.2%	0.25% ±0.2%	0±0.2%	0±0.2%
	2ng	20% ±2%	10% ±1%	5% ±0.5%	1% ±0.2%	0.5% ±0.2%	NA	0±0.2%	0±0.2%
	1ng	20% ±2%	10% ±1%	5% ±0.5%	1% ±0.2%	NA	NA	0±0.2%	0±0.2%
LLOQ	5ng	20% ±2%	10% ±1%	5% ±0.5%	1% ±0.2%	NA	NA	0±0.2%	0±0.2%
	2ng	20% ±2%	10% ±1%	5% ±0.5%	NA	NA	NA	0±0.2%	0±0.2%
	1ng	20% ±2%	10% ±1%	5% ±0.5%	NA	NA	NA	0±0.2%	0±0.2%

Deviation from Plan:

The acceptance criteria for the validation deviates from that described in the validation plan as the LLOD and LLOQ was initially stated as %VAF ± a set range and based on a theoretical 100% efficiency of droplet generation. It became evident that the number of signal positive droplets achieved in an assay was significantly lower than the predicted number of copies, which formed the basis of the acceptance criteria in the plan. In addition, the %VAF acceptance range was found not to be feasible at lower cfDNA inputs and VAF frequencies, as the number of mutant positive droplets being counted was very low. In these situations, a single droplet can affect the %VAF much more than at high input and high VAF frequencies. These issues made it very difficult to come up with realistic acceptance criteria based on %VAF that are suitable across all input concentrations and VAF frequencies.

Therefore, to make the acceptance criteria relevant to the assay as a whole and facilitate the acceptance of the widest range of clinical samples, the acceptance

criteria were modified to '*minimum number of mutant positive droplets*'. This modification does not change the sensitivity, repeatability or reproducibility of the assay, but means the assay can be applied across more inputs and VAF frequencies.

Within a ddPCR assay the %VAF LLOD and LLOQ is dependent on the template DNA input, with lower inputs having higher LLOD and LLOQ. Due to the high specificity of the ddPCR assay a LLOD of ≥3 mutant positive droplets and LLOQ of ≥10 mutant positive droplets was set and tested in the validation. This approach reduces the requirement to test many assay input concentrations at various VAF frequencies.

The CACTUS clinical protocol stipulates that the minimum baseline VAF required for inclusion in the trial is 5%. Also, analysis of serial samples in the trial needs to detect an 80% decrease in %VAF, which equates to a minimum VAF of 1% based on a 5% baseline VAF. Therefore, we determined the minimum DNA input required for the detection of ≥3 mutant positive droplets and ≥10 mutant positive droplets using the 5% VAF control sample and 1% VAF control sample.

10ng input was included in the planned experiments to facilitate the interpretation of the change in acceptance criteria.

Modified Acceptance Criteria:

- All Negative control samples must have ≤2 mutant positive droplets with DNA input of up to 10 ng per reaction
- Known mutant positive samples must have ≥3 mutant positive droplets with 5ng and 10 ng DNA inputs per reaction
- cfDNA input requirement must be ≤5 ng to achieve ≥10 mutant positive droplets with 5% VAF

Results:

To demonstrate the VAF LLOD and LLOQ, mutant positive cell-line DNA was mixed with wild-type cell-line DNA in a serial dilution experiment to generate five samples of varying levels of mutant %VAF (20%, 10%, 5%, 1% and 0%). Each of these controls was then assayed at four input concentrations (10ng, 5ng, 2ng and 1ng).
From this data (Summarised in: 'Combined data' Tab. Filename: 'Validation data 2.3') the acceptance criteria set in the initial validation plan was found to fail for every experimental sample due to the issues discussed previously. Utilising the modified acceptance criteria the number of mutant positive droplets was measured for each input level and is tabled below (**Table 14**).

Project Code	CEP-0359
Short Title	Validation of ddPCR BRAF V600 Assay

This data shows that at all cfDNA inputs tested, every negative control ('0% VAF') had no mutant positive droplets, with mutant positive droplets only ever detected in samples known to be positive.
Related experiments: 359782021 & 359782022

Table 14. Number of mutant positive droplets generated for known VAF controls at various cfDNA inputs.

	Number of Mutant Positive Droplets at various cfDNA input concentrations			
% VAF	10 ng	5 ng	2 ng	1 ng
20%	161	91	36	15
10%	73	43	22	8
5%	42	21	10	5
1%	7	5	2	1
0%	0	0	0	0
Values in green are ≥10 Mutant Positive Droplets				
Values in Orange are ≥3 but <10 Mutant Positive Droplets				
Values in Red are <3 Mutant Positive Droplets				

(Table location: '2.3 Figures' Tab. Filename: 'Validation data 2.3')

To calculate the absolute minimum DNA input required for the detection of ≥3 mutant positive droplets and ≥10 mutant positive droplets at a 5% VAF and 1% VAF, the 5% and 1% VAF data were utilised to generate a standard curve for each VAF control (**Figure 2**).

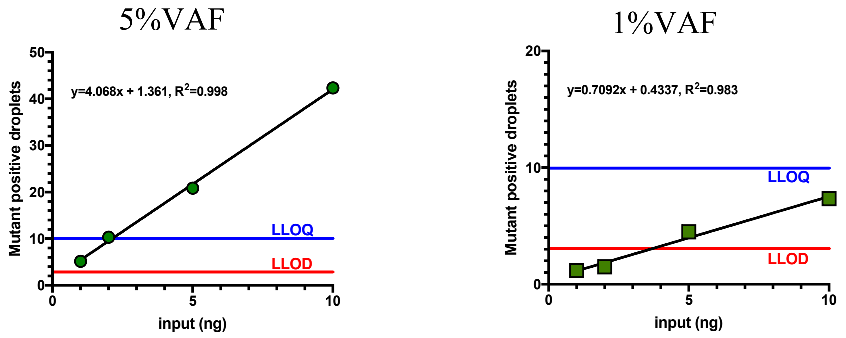

Figure 2. Standard curves of number of mutant droplets generated at various DNA inputs at 5% and 1% VAF. LLOQ and LLOD are indicated on each graph with blue and red lines respectively. The equation of the line of best fit is shown for each graph and was used to extrapolate input amounts required for ≥3 and ≥10 mutant positive droplets at each VAF%.

(Analysis performed in Graphpad, Filename: 'Validation data 2.3.pzfx')

The standard curve for the 5% VAF control was found to be reliable with a R^2 value = 0.998 (**Figure 2**). Using this data, the lowest input for the assay to detect ≥3 mutant positive droplets (LLOD) was shown to be 0.40ng and 2.12ng to detect ≥10 mutant positive drop (LLOQ) as shown in **Table 15**.

Project Code	CEP-0359
Short Title	Validation of ddPCR BRAF V600 Assay

Table 15. Calculation of DNA input LLOD and LLOQ for 5% and 1% VAF.

		LLOD		LLOQ	
		no. droplets	cfDNA Input (ng)	no. droplets	cfDNA Input (ng)
5% VAF	y=4.068x + 1.361	3	0.40	10	2.12
1% VAF	y=0.7092x + 0.4337	3	3.62	10	13.49

When reducing the %VAF down to 1% the estimated LLOD and LLOQ for DNA input increases to 3.62ng and 13.49ng respectively (**Table 15**).

- The assay met the acceptance criteria that all negative control samples must have ≤2 mutant positive droplets with DNA input of up to 10 ng per reaction

- The assay met the acceptance criteria that known mutant positive samples should have ≥3 mutant positive droplets with 5ng and 10 ng DNA inputs per reaction

- The assay met the acceptance criteria cfDNA input requirement must be ≤5 ng to achieve ≥10 mutant positive droplets with 5% VAF (DNA input LLOQ = 2.12ng for 10 droplets at 5%VAF).

Conclusions:

The data demonstrates that a minimum cfDNA input of 2.12ng is required to quantify the requirement of 5% VAF in baseline samples. This confirms that a planned default cfDNA input of 5ng for CAcTUS baseline samples will generate a quantifiable result.
In order to determine an 80% drop in %VAF from a 5% VAF baseline sample, a minimum cfDNA input of 13.49ng is required to quantify the requirement of 1% VAF in serial samples.
The LLOD and LLOQ were not determined as originally indicated in the plan. However, justifying a fixed number of droplets to define the LLOD and LLOQ has demonstrated the requirements of the practical application of the assay to the CAcTUS trial. The implications of this information for its use in the CAcTUS trial are discussed in **Sections 10.1 (3) and 10.2**.

9.4. Validation of mutant and wild type QC controls

This refers to section 2.4 of the validation plan

Aim: To demonstrate the accuracy, reproducibility and repeatability of a mutant and wild type controls for each ddPCR V600 assay.

Project Code	CEP-0359
Short Title	Validation of ddPCR BRAF V600 Assay

Acceptance Criteria:

- Mutant / Wild type VAF for BRAF V600E must be 10% ± 1.0%
- Mutant / Wild type VAF for BRAF V600K must be 10% ± 1.0%
- Mutant / Wild type VAF for BRAF V600R must be 10% ± 1.0%
- Wild type QC will be mutant negative. ≤2 copies per 1515 (<0.2%)

Deviation from Plan:

The Acceptance Criteria for determining the QC %VAF were re-phrased to clarify the original intention and avoid ambiguity in the report. It was intended to mean 10% ± 1, inferring a target range of 9.0 to 11.0

In addition, the term '%' from the acceptance range was removed as discussed previously in **Section 9.1**.

Re-phrased acceptance criteria:

- The determined %VAF for the BRAF V600E QC must be between 9.0 and 11.0
- The determined %VAF for the BRAF V600K QC must be between 9.0 and 11.0
- The determined %VAF for the BRAF V600R QC must be between 9.0 and 11.0
- Wild type QC will be mutant negative, giving ≤2 mutant positive droplets per 1515 signal positive droplets

The acceptance criteria for Reproducibility and Repeatability were not defined in the plan within this section. However, those described in section 9.1 were used to assess this data.
The acceptance criteria for mutant positive QCs would be ±5 of their determined %VAF for both Reproducibility and Repeatability.
Acceptance for the Wild Type QC (negative control) would be <1 %VAF for both Reproducibility and Repeatability, based on the specificity acceptance of <1 %VAF.

Results:

Four fragmented cell-line DNA samples (Mutant variants, BRAF V600E, BRAF V600K & BRAF V600R and the WT allele) were used to generate mutant and wild type controls for each ddPCR V600 assay.
4 wells of 5ng input of each QC was used to determine a %VAF and demonstrate the reproducibility and repeatability of the QC's for their respective assays, across 5 independent experiments.

Related experiments:
359782019, 359782020, 359782023, 359782024 & 359782025

Project Code	CEP-0359
Short Title	Validation of ddPCR BRAF V600 Assay

Table 16. %VAF determined for the mutant and wild type QC:

ddPCR Assay		Positive Control	Results	Negative Control	Results
BRAF V600E	Determined %VAF	A375 (BRAF V600E)	9.15	RM59 (BRAF V600 WT)	0.00
	Plan Acceptance Range		9.00 - 11-00		0.00 - 0.20
	Outcome		**Pass**		**Pass**
BRAF V600K	Determined %VAF	RM44 (BRAF V600K)	10.95	RM59 (BRAF V600 WT)	0.00
	Plan Acceptance Range		9.00 - 11-00		0.00 - 0.20
	Outcome		**Pass**		**Pass**
BRAF V600R	Determined %VAF	M381 (BRAF V600R)	9.04	RM59 (BRAF V600 WT)	0.00
	Plan Acceptance Range		9.00 - 11-00		0.00 - 0.20
	Outcome		**Pass**		**Pass**

(Table location: '2.4 Figures' Tab. Filename: 'Validation data 2.4')

Table 17. Reproducibility and Repeatability of the mutant and wild type QCs:

Sample	ddPCR Assay		Experiment Number					Inter-assay
			359782019	359782020	359782023	359782024	359782015	%VAF
%VAF BRAF V600 Mutant	BRAF V600E	Determined %VAF	8.91	9.18	8.81	9.87	9.00	9.15
		Measured Range	7.16 to 10.52	7.97 to 10.10	7.85 to 10.50	9.06 to 11.56	8.07 to 9.81	8.81 to 9.87
		Acceptance Range*	3.91 to 13.91	4.18 to 14.18	3.81 to 13.81	4.87 to 14.87	4.00 to 14.00	4.15 to 14.15
		Outcome	**Pass**	**Pass**	**Pass**	**Pass**	**Pass**	**Pass**
	BRAF V600K	Determined %VAF	11.06	11.22	11.25	10.55	10.66	10.95
		Measured Range	10.62 to 11.63	10.84 to 11.63	10.56 to 12.50	8.98 to 11.20	10.02 to 11.60	10.55 to 11.22
		Acceptance Range*	6.06 to 16.06	6.22 to 16.22	6.25 to 16.25	5.55 to 15.55	5.66 to 15.66	5.95 to 15.95
		Outcome	**Pass**	**Pass**	**Pass**	**Pass**	**Pass**	**Pass**
	BRAF V600R	Determined %VAF	9.29	8.96	8.37	8.72	9.87	9.04
		Measured Range	8.11 to 10.52	8.03 to 9.41	4.18 to 11.46	7.29 to 10.43	8.50 to 10.98	8.37 to 9.87
		Acceptance Range*	4.29 to 14.29	3.96 to 13.96	3.37 to 13.37	3.72 to 13.72	4.87 to 14.87	4.04 to 14.04
		Outcome	**Pass**	**Pass**	**Pass**	**Pass**	**Pass**	**Pass**
%VAF BRAF V600 Wild Type	BRAF V600 WT (V600E)	Determined %VAF	0.00	0.00	0.00	0.00	0.00	0.00
		Measured Range	0.00 to 0.00	0.00 to 0.00	0.00 to 0.00	0.00 to 0.00	0.00 to 0.00	0.00 to 0.00
		Acceptance Range*	0.00 to 0.20	0.00 to 0.20	0.00 to 0.20	0.00 to 0.20	0.00 to 0.20	0.00 to 0.20
		Outcome	**Pass**	**Pass**	**Pass**	**Pass**	**Pass**	**Pass**
	BRAF V600 WT (V600K)	Determined %VAF	0.00	0.00	0.00	0.00	0.00	0.00
		Measured Range	0.00 to 0.00	0.00 to 0.00	0.00 to 0.00	0.00 to 0.00	0.00 to 0.00	0.00 to 0.00
		Acceptance Range*	0.00 to 0.20	0.00 to 0.20	0.00 to 0.20	0.00 to 0.20	0.00 to 0.20	0.00 to 0.20
		Outcome	**Pass**	**Pass**	**Pass**	**Pass**	**Pass**	**Pass**
	BRAF V600 WT (V600R)	Determined %VAF	0.00	0.00	0.00	0.00	0.00	0.00
		Measured Range	0.00 to 0.00	0.00 to 0.00	0.00 to 0.00	0.00 to 0.00	0.00 to 0.00	0.00 to 0.00
		Acceptance Range*	0.00 to 0.20	0.00 to 0.20	0.00 to 0.20	0.00 to 0.20	0.00 to 0.20	0.00 to 0.20
		Outcome	**Pass**	**Pass**	**Pass**	**Pass**	**Pass**	**Pass**

*Acceptance Range is ±5 of the determined inter-assay %VAF for that QC / Assay

(Table location: '2.4 Figures' Tab. Filename: 'Validation data 2.4')

The intention of this section was to determine an acceptance range for the controls suitable for use in the CAcTUS trial. The experimentally determined %VAF for the 3 positive controls from 5 experiments was within the acceptance range of 9% - 11% [10%±1] (**Table 16**).

The ranges of the experimentally determined values were 8.81-9.87 for BRAF V600E, 10.55-11.25 for BRAF V600K and 8.37-9.87 for BRAF V600R. Each range was within ±1 of their respective means and the deviation of the mean from the target 10% is due to technical variation associated with generation of the QC material.

No mutant positive droplets were measured in 30 reaction wells (duplicate wells, three assays, 5 experiments); meeting the acceptance criteria of ≤2 mutant positive droplets per 1515 signal positive droplets (**Table 17**).

Project Code	CEP-0359
Short Title	Validation of ddPCR BRAF V600 Assay

All 4 reaction wells gave a %VAF within the range of ±5 of the determined %VAF (**Table 17**), which infers the use of the controls is reproducible.

In addition, the values for each replicate experiment gave %VAFs within ±5 of the inter assay %VAF (**Table 17**), demonstrating the use of the controls is repeatable.

Conclusions:

The data demonstrates an acceptable mean %VAF and suitable reproducibility and repeatability such that the QCs are considered valid for use in future analysis.

The determined %VAF for each assay was within ±1 of the inter-assay %VAF and as a result the mutant and wild type QC acceptance criteria was set to ±1 of the inter-assay %VAF for use in future ddPCR assays (**Table 18**).

Table 18. Acceptance Criteria of the mutant and wild type QCs for future assays:

ddPCR Assay	QC Type	QC CHEMLOG Reference	Acceptance Range
BRAFV600E	Positive Control	7092	8.15 - 10.15
BRAFV600K	Positive Control	7093	9.95 - 11.95
BRAFV600R	Positive Control	7094	8.04 - 10.04
BRAF V600 WT (V600E)	Negative Control	7095	0.00 to 0.20
BRAF V600 WT (V600K)	Negative Control	7095	0.00 to 0.20
BRAF V600 WT (V600R)	Negative Control	7095	0.00 to 0.20

(Table location: '2.4 Figures' Tab. Filename: 'Validation data 2.4')

9.5. Verification of ending thermal cycling at 12°C and 4°C

This refers to section 2.5 of the validation plan.

Aim: To demonstrate that ending the thermal cycling at either 12°C or 4°C will not affect the data generated.

Acceptance Criteria:
The mean VAF for the two sets of data must be within 5% of each other.

Results:

The temperature at which the thermal cycling is ended will be qualified using 5 experiments. 3 experiments will be ended at the 12°C hold and 2 experiments will be ended at 4°C
Related experiments:
359782019, 359782020, 359782023, 359782024, 359782025

Table 19. Verification of ending thermal cycling at 12°C and 4°C

Experiment Number	Thermal Cycler end	%VAF BRAF V600 Mutant					
		V600 E	V600 K	V600 R	V600 WT (V600E)	V600 WT (V600K)	V600 WT (V600R)
359782019	12°C	8.91	11.06	9.29	0.00	0.00	0.00
359782020	12°C	9.18	11.22	8.96	0.00	0.00	0.00
359782024	12°C	8.81	11.25	8.37	0.00	0.00	0.00
359782023	4°C	9.87	10.55	8.72	0.00	0.00	0.00
359782025	4°C	9.00	10.66	9.87	0.00	0.00	0.00
Mean of 12°C experiments		8.96	11.18	8.87	0.00	0.00	0.00
Mean of 4°C experiments		9.43	10.61	9.30	0.00	0.00	0.00
Difference between %VAF (12°C-4°C)		0.47	0.57	0.43	0.00	0.00	0.00
Percentage Difference in %VAF		4.98	-5.39	4.59	0.00	0.00	0.00
%VAF difference <5% (Pass/Fail)		Pass	FAIL	Pass	Pass	Pass	Pass
SD of 4°C experiments		0.61	0.08	0.81	0.00	0.00	0.00
SD of 12°C experiments		0.19	0.10	0.47	0.00	0.00	0.00

(Table location: '2.5 Figures' Tab. Filename: 'Validation data 2.4')

The BRAF V600E assay gave a difference in the mean %VAF between 12°C and 4°C of 4.98%, meeting the acceptance criteria of <5%.

The BRAF V600K assay gave a difference in the mean %VAF between 12°C and 4°C of 5.39%, failing to meet the acceptance criteria of <5%.

The BRAF V600R assay gave a difference in the mean %VAF between 12°C and 4°C of 4.59%, meeting the acceptance criteria of <5%.

Conclusions:

Though one data set failed to meet the acceptance criteria, as discussed previously the use of %VAF ±% is inappropriate for these assays. The use of a '±' range is more suitable to the practical use of the assay as described previously in section 9.1. The thermal cycling of 3 experiments ended with a 12°C hold and 2 experiments ended with a 4°C hold gave %VAF values that were within ±0.6 of each other (12°C %VAF - 4°C %VAF). The data demonstrates results within a range of ±1, which is consistent with data generated using the QCs (**Section 9.4**) and below the ±5 range established for the reproducibility and repeatability (**Section 9.1**).

The use of 12°C or 4°C as the holding temperature is considered not to adversely affect the measured %VAF. Therefore, use of either holding temperature is considered acceptable.

9.6. Quantification of VAF in clinical samples

This refers to section 2.6 of the validation plan.

Project Code	CEP-0359
Short Title	Validation of ddPCR BRAF V600 Assay

Aim: To demonstrate the accuracy and repeatability of quantification of mutant VAF in a panel of 10 patient derived test samples.

Acceptance Criteria:

The VAF values determined from the duplicate experiments must be the same ±10% VAF. Also, the mean VAF determined in the duplicate experiments must be ±7% of expected VAF (or ±3 positive droplets at lower VAF frequencies) of the previously measured values *(The VAF's for each sample has previously been determined by ddPCR and NGS and will be accessed after the analysis of the raw data and generation of the VAF values).*

Deviation from Plan:

The acceptance criteria in the plan were re-phrased to clarify the original intention and avoid ambiguity as discussed previously in section 9.1, with the removal of the term '%' from the acceptance range.
It was intended to state:

- The VAF values determined from the duplicate experiments must be within ±10 of each other.
- The mean VAF determined in the duplicate experiments must be within ±7 of previously determined %VAF.

Results:

Ten patient derived cfDNA samples for BRAF V600E (x3), BRAF V600K (x3) and BRAF V600R (x4) had their VAF determined in two independent experiments. Reproducibility and accuracy of the %VAF was demonstrated using the data from between experiments and the %VAF compared to previously determined values respectively.
Related experiments: 359782027 & 359782028

Table 20. Quantification of VAF in clinical samples

cfDNA barcode	V600 Mutation	EXP: 359782027	EXP: 359782028	Difference	%VAF from 2 experiments within ±10 of each other (Pass / Fail)	Measured %VAF	Previously Determined %VAF	Difference	Measured %VAF within ±7 of the Previously Determined %VAF (Pass / Fail)
CEP00025005	V600R	11.56	10.47	-1.1	Pass	11.02	9.04	-2.0	Pass
CEP00025006	V600R	64.98	64.36	-0.6	Pass	64.67	60.63	-4.0	Pass
CEP00025007	V600R	1.56	1.47	-0.1	Pass	1.52	1.38	-0.1	Pass
CEP00025008	V600R	0.00	0.04	0.0	Pass	0.02	0.00	0.0	Pass
CEP00025009	V600K	2.17	2.03	-0.1	Pass	2.10	1.78	-0.3	Pass
CEP00025010	V600K	8.30	8.42	0.1	Pass	8.36	6.46	-1.9	Pass
CEP00025011	V600K	0.00	0.00	0.0	Pass	0.00	0.00	0.0	Pass
CEP00025012	V600E	0.00	0.00	0.0	Pass	0.00	0.00	0.0	Pass
CEP00025013	V600E	44.03	44.77	0.7	Pass	44.40	44.50	0.1	Pass
CEP00025014	V600E	0.43	0.71	0.3	Pass	0.57	1.18	0.6	Pass

(Table location: '2.6 Figures' Tab. Filename: 'Validation data 2.6')

Project Code	CEP-0359
Short Title	Validation of ddPCR BRAF V600 Assay

The difference in the measured %VAF of the duplicate experiments was within ±2 of each other in all 10 patients, meeting the acceptance criteria of ±10 (**Table 20**).

The difference in the mean %VAF of the duplicate experiments was within ±5 of the reported %VAF in all 10 patients, meeting the acceptance criteria of ±7 (**Table 20**).

Conclusion:

As discussed in **Section 9.1**, the use of a '±' range is more suitable to the practical use of the assay. An acceptable range of ±1 would be consistent with the data generated using the QCs (**Section 9.4**) and below the ±5 range for the reproducibility and repeatability (**Section 9.1**).

Duplicate experiments were within ±1 for 9 of the 10 patient samples, one sample giving a difference of 1.1. The difference of 1.1 is still meets the ±1 acceptance criteria as it rounds to 1.

All 10 patient samples gave mean %VAF within ±5 of the reported value indicating the assay in our hands is comparable to external data. The accuracy of repeat analysis would be dependent upon many factors which include inter-platform variation, site-to-site variation, sample handling control and sample storage. Cross comparison with other sites is not a requirement for the CAcTUS trial.

9.7. IQ/OQ/PQ of the ddPCR equipment

This refers to section 2.7 of the validation plan

Aim: To qualify the ddPCR equipment as part of the validation

Acceptance Criteria (based on manufactures guidance):

The **OQ** for the Droplet generator: Total number of **droplets created must be ≥8,000**

The **OQ** for the thermal cycler:
Mutant / Wild type VAF for BRAF V600E = pass acceptance set in **section 2.4** [of the plan]
Mutant / Wild type VAF for BRAF V600K = pass acceptance set in **section 2.4** [of the plan]
Mutant / Wild type VAF for BRAF V600R = pass acceptance set in **section 2.4** [of the plan]
Wild type QC will be mutant negative **<5 copies per 1000 (0.5%)**

Project Code	CEP-0359
Short Title	Validation of ddPCR BRAF V600 Assay

The **OQ** for the Droplet reader:
Be able to read the plate successfully and save a data file.
Be able to analyse the data and generate concentrations and VAF percentage as described for the thermal cycler.

The **OQ** for the plate sealer consist of a visual inspection and the ability to seal the plates according to the manufacturer's instructions.

Results:

All IQ/OQ/PQ records of the equipment listed in **Table 1** (section 7) are documented in the respective asset records in Q-Pulse.

Installation qualification did not form part of the plan acceptance criteria, but was performed by an engineer from the manufacturer prior to OQ/PQ.

OQ/PQ was achieved by a combination of work by the service engineer, the asset owner and data contained in this validation report. The outcomes are summarised in **Table 21** and recorded in the asset records in Q-Pulse.

Table 21. OQ/PQ of the ddPCR Equipment

Operational / Performance Qualification	Acceptance criteria	Data Source / Evidence	Pass / Fail
The **OQ/PQ** for the Droplet generator:	Total number of **droplets created must be ≥8,000**	Veryfying droplet event count was performed as part of the IQ bt the service engineer and recorded in the Q-Pulse asset record	Pass
The **OQ/PQ** for the thermal cycler:	Mutant/Wild type VAF for BRAF V600E = pass acceptance set in section 2.4 of the validation plan	Section 9.4 of the validation report	Pass
	Mutant/Wild type VAF for BRAF V600K = pass acceptance set in section 2.4 of the validation plan	Section 9.4 of the validation report	Pass
	Mutant/Wild type VAF for BRAF V600R = pass acceptance set in section 2.4 of the validation plan	Section 9.4 of the validation report	Pass
	Wild type QC will be mutant negative **<5 copies per 1000 (0.5%):**	Section 9.2 of the validation report	Pass
The **OQ/PQ** for the Droplet reader:	Be able to read the plate successfully and save a data file	Performed following the IQ and recorded in the Q-Pulse asset record	Pass
	Be able to analyse the data and generate concentrations and percentage VAF	Performed following the IQ and recorded in the Q-Pulse asset record	Pass
The **OQ/PQ** for the plate sealer:	A visual inspection and the ability to seal the plates according to the manufacturer's instructions	Performed following the IQ and recorded in the Q-Pulse asset record	Pass

(Table location: 'sheet 1' Tab. Filename: 'Validation data 2.7')

Project Code	CEP-0359
Short Title	Validation of ddPCR BRAF V600 Assay

Conclusion:

Installation Qualification of the ddPCR equipment was performed prior to validation on the 19/20-Dec-2017 by a Bio-Rad service engineer and all tests passed their acceptance criteria specified by the manufacturer (IQ documentation is stored within the asset record in Q-Pulse).

Operational Qualification / Performance qualification of the Droplet Generator was performed prior to validation on the 19-Dec-2017 by a Bio-Rad service engineer and passed its acceptance criteria as set out in the validation plan (**Table 21**).

Operational Qualification / Performance qualification of the Droplet Reader was performed prior to validation on the 21-Dec-2017 by asset owner and passed its acceptance criteria as set out in the validation plan (**Table 21**).

Operational Qualification / Performance qualification of the Thermal Cycler was performed as part of the validation and passed its acceptance criteria as set out in the validation plan (**Table 21**). Note: The acceptance criteria for the OQ/PQ of 'Wild type QC will be mutant negative <5 copies per 1000 (0.5%)' for the Thermal Cycler is based on the manufacturers recommendations and that the validation of these assays requires ≤2 copies per 1000 (0.2%). The data used (from section 9.2 of this report) also passes this more stringent acceptance criterion.

Operational Qualification / Performance qualification of the Plate Sealer was performed prior to validation on the 21-Dec-2017 by asset owner and passed its acceptance criteria as set out in the validation plan (**Table 21**).

10. Summary and Conclusions

The main objective of this validation was to demonstrate the suitability of the BRAF V600 ddPCR assay for use in the CAcTUS trial. Most of the acceptance criteria set out in the validation plan were met. Those components that did not fully meet their acceptance criteria were either due to a lack of clarity in the original validation plan or unforeseen technical issues with the assay that became apparent during the validation. The impact of the failed criteria on the use of the assays is discussed and any modifications justified.

10.1. Summary of the Component Objectives

1) Specificity, sensitivity and reproducibility of ddPCR assays (**Section 9.1**)

   All assays met the acceptance criteria for specificity (**Table 6**) demonstrating that each ddPCR assay only amplifies material for which it is intended.
   All assays did not meet the acceptance criteria of <5% for reproducibility and repeatability (**Table 7**), however the CV was demonstrated to be <10%

in both cases. The mechanics of population analysis on individual droplets containing high levels of fluorescent signal indicate that the standard deviation is a better indicator of variability of the assay. This shows a maximum variation of ±3 of the mean %VAF for single plex assays.

Table 22. Predicted ranges of experimentally determined %VAF for various %VAFs

Sample %VAF	Predicted Range for Experimentally Determined %VAF
50%	47% to 53%
25%	22% to 28%
10%	7% to 13%
7.5%	4.5% to 10.5%
5%	2% to 8%
2%	0% to 5%
1.5%	0% to 4.5%
1%	0% to 4%

Using the variation ±3, **Table 22** shows the anticipated range in an experimental result for a given %VAF. Monitoring an 80% drop in %VAF from a baseline result would require a baseline value >7.5% in order to distinguish the %VAF that gives an 80% drop (from 7.5% to 1.5%) without any overlap.
A baseline VAF of 5% gives the possibility that the 80% drop may not be observed due to an overlap with the 1% VAF range. Assuming a normal distribution and an average standard deviation of 1.9, 15% of the determined %VAF would overlap. Therefore a baseline %VAF between 7.5% and 5.0% has a 15% maximum risk of not determining the 80% drop accurately. The risk of not determining the 80% drop accurately increases as the baseline Sample % VAF decreases (7.5% VAF = 0% risk, 6.2% VAF = 5% risk, 5% VAF = 15% risk; reference: *Normal Distribution.ppt*)

2) <u>The LOB (**Section 9.2**)</u>

No false positive droplets were observed in all validation experiments, meeting the acceptance criteria and leading to the conclusion that the assays have a very low % false positive rate. This was demonstrated to be <0.13% for negative controls and HNV samples (**Table 11**).

The data supports the assertion that samples which gives 3 or more mutant positive droplets (the LLOD for this assay) provides a valid positive result.

The data has demonstrated the false positive rate for NTCs to be tenfold lower that negative controls and HNV samples. Therefore NTC wells are not required in an assay where a suitable negative control sample is used.

Project Code	CEP-0359
Short Title	Validation of ddPCR BRAF V600 Assay

3) The LLOD and LLOQ (**Section 9.3**)

Due to the unforeseen discrepancy between the measured number of amplified droplets and the predicted copy number for various DNA inputs, the acceptance criteria for LLOD and LLOQ was not met. However, the purpose of this validation was to demonstrate that the assay is fit for use in the CAcTUS trial, so the data has been assessed based on the rationale discussed in **Section 9.3**.

The data demonstrated:

- A minimum DNA input of 2.12ng is required to quantify the requirement of 5% VAF in baseline samples
- A minimum DNA input of 13.49ng is required to quantify the requirement of 1% VAF in serial samples
- Negative control samples gave no mutant positive droplets

As stated, the acceptance criteria described in the validation plan were not met; however, the rationale discussed demonstrates that the LLOD and LLOQ applied using the minimum number of droplets ensured the assay is valid for use within the DNA input and %VAF constraints documented in **Section 10.2** (**Table 23**).

4) Reproducibility and repeatability of assay QCs (**Section 9.4**)

The data using the positive and negative QC controls for the ddPCR assays (**Section 9.4**) met all their acceptance criteria and is therefore valid for use.

5) Verification of ending thermal cycling at the 12˚C and 4˚C (**Section 9.5**)

Comparing the data of the two end thermal cycling settings demonstrated that there was no significant influence on the end result of the assay and both are therefore suitable for use.

6) Quantification of VAF in clinical samples (**Section 9.6**)

Analysis of the clinical samples in consecutive experiments and comparison to the unblinded %VAF provided by the sponsor gave reproducible and repeatable data that met the acceptance criteria.

7) IQ/OQ/PQ of the ddPCR equipment (**Section 9.7**)

The ddPCR equipment was deemed fully qualified and fit for use in clinical trials.

Project Code	CEP-0359
Short Title	Validation of ddPCR BRAF V600 Assay

8) Multiplex ddPCR

Experiments using the three-plex ddPCR assay were performed in some experiments and reported in **Section 9**. However, the data indicates the variation in the assay may be raised compared to single plex assays and further work would be required to draw specific conclusions regarding the suitability of the multiplex assay for use in routine analysis.

10.2. Summary of the Overall Objectives

The objective of detecting an 80% drop in %VAF in serial patient samples from a baseline of ≥5% VAF using an acceptance of 1ng DNA input to detect ≥5% VAF and 5ng DNA input for 1% VAF was not met. However, by modifying the sample input for samples likely to give low %VAF values the assay can be used successfully.

Section 10.1 summarises a rationale that shows suitable parameters in which the 80% drop in %VAF can be accurately determined.

The acceptance criteria for use in the CAcTUS trial **(outlined in Section 10.3)** is considered to have achieved the overall objective and is therefore valid for use.

10.3. Acceptance Criteria for use in the CAcTUS Trial

Table 23 outlines the acceptance criteria for use in the CAcTUS Trial when following the method used for the validation (RSP-52, to be implemented as SOP-106 post validation, for routine sample analysis).

Table 23. Acceptance Criteria for BRAF V600 single plex Assays

Parameter	Acceptance Criteria	Comments
The Negative control for the assay	<0.13% Mutant VAF	Failure makes Assay Invalid
The Positive control for the assay	Must be within the acceptance range logged for that QC	Failure makes Assay Invalid
Sample result below the minimum number of signal positive droplets	>100 signal positive droplets	%VAF may be reported, but must be flagged to highlight that the accuracy of the result may be compromised due to low numbers of signal positive droplets
Sample result below the droplet LLOD	≤2 mutant positive droplets	Sample reported as below the LLOD

Project Code	CEP-0359
Short Title	Validation of ddPCR BRAF V600 Assay

Parameter	Acceptance Criteria	Comments
		(No %VAF Value given)
Sample result between the droplet LLOD and droplet LLOQ	Between 3 and 9 mutant positive droplets	Sample reported as below the LLOQ (with %VAF value given)
Sample result above the droplet LLOQ	≥10 mutant positive droplets	Valid %VAF result reported
Minimum DNA input quantity	There is no minimum limit for DNA input	Validity is based on droplet numbers not DNA input
Varying DNA input quantity	Suitable levels are outlined in **Table 24**	DNA input levels will be recorded but not reported
Duplicate experiments of Baseline samples	Subtraction of the first %VAF from the second %VAF must be ±2	The Mean and SD %VAF of the 2 experiments is reported<br>For subtraction results >±2 the result must be flagged with a comment to aid interpretation, stating the raised difference*.
Baseline Values with a determined %VAF between 5.0% and 7.5%	No associated Acceptance criteria<br>The ability to detect an 80% drop may be compromised	Serial sample data must be reported with a clarification comment to aid interpretation*.
Total number of accepted droplets generated by the droplet generator	>8,000 per reaction well	The individual well is invalid<br>This may be reported as a research result, but must be flagged as an invalid result
*The wording of the comments to aid interpretation will be agreed prior to commencement of the trial and documented in the trial project plan		

When the determined %VAF of a patient baseline sample falls below 10%, analysis of the serial samples may involve increasing the DNA input level to compensate for the higher LLOQ at lower %VAF (assuming enough material is available).

The validity of the assay is not compromised providing a maximum of 5ng of DNA is used in individual reactions (per well). Merging multiple wells containing 5ng per well will increase the chances of meeting the requirement for the LLOQ and maintain validity.

Suggested guidelines are given in **Table 24**.

Project Code	CEP-0359
Short Title	Validation of ddPCR BRAF V600 Assay

Table 24. Strategy for modifying DNA input levels

Predicted %VAF	DNA Input LLOD (ng)	DNA Input LLOQ (ng)	Strategy
≥5%	0.4 (For 5%)	2.12 (For 5%)	If available, add 5ng in up to 4 wells
>2.5% to <5%	N/A	N/A	If available, add 5ng in up to 4 wells
>1.5% to <2.5%	N/A	N/A	If available, add 5ng per well, up to 15ng in total.
>1% to <1.5%	3.62 (For 1%)	13.49 (For 1%)	Add as much sample as possible. If available, add 5ng per well, up to 20ng in total.
≤1%	N/A	N/A	Add as much sample as possible. Up to 5ng per well, multiple wells

10.4. Overall conclusion and statement of validity

The data in this validation report supports the conclusion that it is sufficiently robust within the defined parameters to consider it is valid for use as a primary endpoint assay for the CAcTUS trial.

It may also be valid for additional trials providing a suitable assessment is performed to demonstrate that the same parameters are applicable.

- Validation of single plex ddPCR assay BRAF V600E is considered successful.
- Validation of single plex ddPCR assay BRAF V600K is considered successful.
- Validation of single plex ddPCR assay BRAF V600R is considered successful.
- The data using multiplex ddPCR assay (BRAF V600E/K/R) is considered to be insufficient to warrant validity for use in routine GCP analysis.
- The ddPCR equipment is considered to be fully qualified for use to GCP.

11. Storage of Data

All validation experiments were recorded in laboratory notebook **CN/782**.

All raw data generated are stored in GCLP directory ‘GCLP\Projects\CEP359\Raw Data (read only)’.

All analysis files generated are stored in GCLP directory ‘GCLP\Projects\CEP359\Analysis’

All the interpretation files used to compare data and generate figures for the report are stored in GCLP directory ‘GCLP\Projects\CEP359\Analysis’.

12. Archiving of Data

12.1. All paper documents relating to this study will be held in a GCP compliant archive for a period of at least 5 years.

12.2. All electronic files relating to this study will be stored securely in CEP-359 project folder within the GCLP Drive of the CRUK-MI IT Network for a period of at least 5 years.

12.3. Disposal of documents (paper or electronic) will occur following contact and consent of the study sponsor either (i) at the end of the archiving period or (ii) when the method is no longer required, whichever is longer.

13. Compliance Statement for Data Audit Trail

13.1. Data generated as part of this validation were audit trailed throughout the whole process from sample receipt to this final report to ensure the study has been conducted in compliance to GCP and an intact data audit trail can be demonstrated. This has been conducted by the QA Team of CEP.

13.2. An audit trail compliance statement can be sent to the trial sponsor under separate cover.

14. Report Distribution List

14.1. Internal Copies

An internal copy will be maintained in the document module of Q-Pulse

14.2. External Copies

None

Project Code	CEP-0359
Short Title	Validation of ddPCR BRAF V600 Assay

Appendix 1. Sponsors clinical assessment of the risks and benefits to the patient in using the data from this assay

CAcTUS TRIAL NOTES AND CLINICAL PERSPECTIVE

Dr Rebecca Lee, Clinical Oncologist

Who has already reviewed and approved the CAcTUS trial?

The trial has been formally peer reviewed by 2 external reviewers both of which gave an extremely favourable opinion. One of the reviewers was the chair of the NCRI Skin CSG at the time of reviewing. In addition Prof. Fiona Blackhall has read the protocol and provided feedback. As the trial is open at 3 other sites the clinicans from those have reviewed the trial. Furthermore we have had informal feedback from key opinion leaders in melanoma (Antoni Ribas and Keith Flaherty). The Chair and Lead clinical pharmacist of NHS England (David Thompson and Peter Clarke) have also reviewed the trial and given special permission for the drug schedule to be funded by the NHS. Finally, all core trial documents have been reviewed by a patient representative.

What the potential patient benefits of the CAcTUS trial?

The potential benefits to patients are that this schedule of treatment results in, a deeper response to immune therapy (nivolumab and ipilimumab), with associated improvement of symptoms, a longer duration of response and improved survival.

What are the patient risks associated with a false ddPCR readout?

There are two scenarios of a false ddPCR readout:

1. The >80% decrease is falsely called resulting in the patient switching treatment earlier. At this stage the patient would still be responding to treatment but it may not be at maximal response. The hypothesis of the trial of an improved response to immune therapy through switching in response to targeted therapy rather than resistance would still hold. The patient will still be re-treated with targeted therapy if they fail immune therapy and therefore they will still access drug, just at a later time. There is data to suggest that this approach in itself could improve outcomes on targeted therapy and therefore we do not see it as a risk. It is important to point out that often patients being treated as standard of care are switched before they become resistant to drug due to toxicity or patient choice and therefore the trial mirrors what can sometimes happens in clinic anyway.

2. The >80% decrease is not called at that timepoint. This would result in a delay in the patient switching treatment. This theoretically would be detrimental to the trial hypothesis, but not to the patient, as current standard of care would be to continue targeted therapy until resistance and therefore the patient would be treated as we would normally do.
