# Supplementary material for: Use of circulating tumour DNA to prospectively guide a switch from targeted to immune therapy in BRAF mutant advanced melanoma: the randomised phase II CAcTUS trial
Source: Nat Commun. 2026 May 21;17:6850. doi: 10.1038/s41467-026-72735-8 (PMC13389006; doi:10.1038/s41467-026-72735-8)
Supplement: Supplementary file 2 — Description of Additional Supplementary Information [file 41467_2026_72735_MOESM2_ESM.pdf]

Description of Additional Supplementary Files

File Name: Supplementary Data 1
Description: Healthy normal volunteer (n=3) negative controls.

File Name: Supplementary Data 2
Description: Summary of ddPCR data for the screening samples (n=44) including those used to determine the threshold to reduce the inclusion criteria.
