# Supplementary material for: Use of circulating tumour DNA to prospectively guide a switch from targeted to immune therapy in BRAF mutant advanced melanoma: the randomised phase II CAcTUS trial
Source: Nat Commun. 2026 May 21;17:6850. doi: 10.1038/s41467-026-72735-8 (PMC13389006; doi:10.1038/s41467-026-72735-8)
Supplement: Supplementary file 4 — Reporting Summary [file 41467_2026_72735_MOESM4_ESM.pdf]

nature portfolio

Corresponding author(s): Rebecca Lee, Paul Lorigan

Last updated by author(s): Rebecca Lee 23/04/26

Reporting Summary

Nature Portfolio wishes to improve the reproducibility of the work that we publish. This form provides structure for consistency and transparency in reporting. For further information on Nature Portfolio policies, see our Editorial Policies and the Editorial Policy Checklist.

Statistics

For all statistical analyses, confirm that the following items are present in the figure legend, table legend, main text, or Methods section.

n/a	Confirmed	
☐	☒	The exact sample size (*n*) for each experimental group/condition, given as a discrete number and unit of measurement
☐	☒	A statement on whether measurements were taken from distinct samples or whether the same sample was measured repeatedly
☐	☒	The statistical test(s) used AND whether they are one- or two-sided<br>*Only common tests should be described solely by name; describe more complex techniques in the Methods section.*
☒	☐	A description of all covariates tested
☒	☐	A description of any assumptions or corrections, such as tests of normality and adjustment for multiple comparisons
☐	☒	A full description of the statistical parameters including central tendency (e.g. means) or other basic estimates (e.g. regression coefficient) AND variation (e.g. standard deviation) or associated estimates of uncertainty (e.g. confidence intervals)
☐	☒	For null hypothesis testing, the test statistic (e.g. *F*, *t*, *r*) with confidence intervals, effect sizes, degrees of freedom and *P* value noted<br>*Give P values as exact values whenever suitable.*
☒	☐	For Bayesian analysis, information on the choice of priors and Markov chain Monte Carlo settings
☒	☐	For hierarchical and complex designs, identification of the appropriate level for tests and full reporting of outcomes
☒	☐	Estimates of effect sizes (e.g. Cohen's *d*, Pearson's *r*), indicating how they were calculated

Our web collection on statistics for biologists contains articles on many of the points above.

Software and code

Policy information about availability of computer code

Data collection	Data was collected using the RedCap Cloud database https://www.redcapcloud.com/
Data analysis	R packages tableone V0.13.2, tidyverse v2.0.0, lubridate v1.9.2, ggplot2 v3.4.2. In addition Quantist v1.0.1, Prism v10.2.2, Quantasoft v1.7.4

For manuscripts utilizing custom algorithms or software that are central to the research but not yet described in published literature, software must be made available to editors and reviewers. We strongly encourage code deposition in a community repository (e.g. GitHub). See the Nature Portfolio guidelines for submitting code & software for further information.

Data

Policy information about availability of data

All manuscripts must include a data availability statement. This statement should provide the following information, where applicable:

- Accession codes, unique identifiers, or web links for publicly available datasets
- A description of any restrictions on data availability
- For clinical datasets or third party data, please ensure that the statement adheres to our policy

Sharing of additional data not within the manuscript or in supplemental will be considered upon request to the Sponsor and Chief Investigator

Research involving human participants, their data, or biological material

Policy information about studies with human participants or human data. See also policy information about sex, gender (identity/presentation), and sexual orientation and race, ethnicity and racism.

Reporting on sex and gender	Reported in Table 1: Biological sex Arm A male=6, female=4; Arm B male=6, female=5. Sex was self-reported. Data on reported gender were not collected.
Reporting on race, ethnicity, or other socially relevant groupings	Ethnicity data was not collected.
Population characteristics	Population characteristics are described in Table 1
Recruitment	Patients were recruited when presenting with advanced melanoma to 12 UK trial sites.
Ethics oversight	North West Greater Manchester Central Research Ethics Committee REF: 19/NW/0046. Translational analysis approval was obtained through the Manchester Cancer Research Centre Biobank ethics application 23_RELE_01 and approval for the specific project under MCRC Biobank Access Committee REF 22/NW/0237

Note that full information on the approval of the study protocol must also be provided in the manuscript.

Field-specific reporting

Please select the one below that is the best fit for your research. If you are not sure, read the appropriate sections before making your selection.

☒ Life sciences ☐ Behavioural & social sciences ☐ Ecological, evolutionary & environmental sciences

For a reference copy of the document with all sections, see nature.com/documents/nr-reporting-summary-flat.pdf

Life sciences study design

All studies must disclose on these points even when the disclosure is negative.

Sample size	The original study sample size target was 40 patients (20 in each arm) based on true feasibility sample size parameters of a i. 95% (or greater) return rate of critical clinical decision dependent blood samples ii. 80% (or greater) success rate of participants on targeted therapy achieving ≥80% BRAF VAF decrease in ctDNA levels enabling estimation of i. the return rate with a two-sided 95% confidence interval of width no more than 12% points (i.e. 87% to 99%) assuming at least 60 critical red blood samples (one sample from each of the 20 Arm A participants and an average of at least two samples from each of the 20 Arm B participants). ii. the success rate of participants on targeted therapy achieving ≥80% BRAF VAF decrease in ctDNA levels with a two-sided 95% confidence interval of width equal to 30% points (i.e., 62%, 92%), assuming that 25% of the participants in Arm A will receive D+T as first line treatment (i.e. 25 participants across arms; 5 in Arm A and 20 in Arm B). On interim review of the return rate, there was good evidence of a high return rate and hence a 95% confidence interval of similar width (14%) could be obtained from a sample of 21 or more participants (resulting in at least 32 critical clinical decision dependent samples, assuming one sample from Arm A participants and at least two samples from Arm B participants). Moreover, 32 samples provided 80% confidence that the lower confidence limit was at least 90% if the true return rate was at least 98%. Likewise, with a sample size of 17 participants with ctDNA reduction data, we could be 80% confident that at least 80% of participants would achieve a reduction in ctDNA levels of at least 80% from baseline, assuming that the true ‘success rate’ percentage was 95% (or greater).
Data exclusions	No data excluded
Replication	Assay reproducibility (%VAF difference between wells of the same experiment) and repeatability (between 3 different experiments) was demonstrated to be within ±5% of the determined %VAF (see Supp. Table 2&3) for all assays.
Randomization	Randomisation was performed centrally via the King’s College London independent randomisation service. Patients were equally randomised to either treatment arm using minimisation with a random element controlling for LDH (<ULN vs. ≥ULN), disease site (<3 sites vs. ≥3 sites), ctDNA VAF (1.5 to ≤10%, >10 to ≤20%, >20%) and M stage (stage III unresectable plus M1a, M1b, M1c and M1d).
Blinding	Blinding was not possible due to the different modalities of treatment given at different times i.e. tablets vs. intravenous infusion.

Reporting for specific materials, systems and methods

We require information from authors about some types of materials, experimental systems and methods used in many studies. Here, indicate whether each material, system or method listed is relevant to your study. If you are not sure if a list item applies to your research, read the appropriate section before selecting a response.

Materials & experimental systems

n/a	Involved in the study	
☒	☐	Antibodies
☒	☐	Eukaryotic cell lines
☒	☐	Palaeontology and archaeology
☒	☐	Animals and other organisms
☐	☒	Clinical data
☒	☐	Dual use research of concern
☒	☐	Plants

Methods

n/a	Involved in the study	
☒	☐	ChIP-seq
☒	☐	Flow cytometry
☒	☐	MRI-based neuroimaging

Clinical data

Policy information about clinical studies

All manuscripts should comply with the ICMJE guidelines for publication of clinical research and a completed CONSORT checklist must be included with all submissions.

Clinical trial registration

NCT03808441

Study protocol

Protocol is provided as supplementary information

Data collection

We randomised 21 patients to the trial between 2nd May 2019 and 30th May 2022, at 12 UK specialist melanoma clinics.

Outcomes

Please refer to the protocol provided for more information.
The study main feasibility outcomes are:

• ctDNA result critical (red) blood sample returned within 7 working days of samples being received in the laboratory.

• Decrease in ctDNA levels of mutant BRAF ≥80% on targeted therapy.

i. Progression free survival (PFS) at 52 (+2) weeks, defined as the time interval from randomisation until confirmed first disease progression according to RECIST v1.1 or death, whichever occurs first. Survival times will be censored at the 52 (+2) weeks trial visit if patients have not progressed, and at the observed time known to be progression-free if participants have a follow up period of less than 52 (+2) weeks, are withdrawn or are lost to follow up.

ii. First Progression Free Survival (PFS), defined as the time interval from randomisation until confirmed first disease progression according to RECIST v1.1 or death, whichever occurs first.

iii. Second Progression Free Survival (PFS) defined as the time interval from randomisation until confirmed second disease progression according to RECIST v1.1 or death, whichever occurs first.

iv. Overall survival (OS), defined as the time interval from randomisation until the documented date of death due any cause.

Plants

Seed stocks

Report on the source of all seed stocks or other plant material used. If applicable, state the seed stock centre and catalogue number. If plant specimens were collected from the field, describe the collection location, date and sampling procedures.

Novel plant genotypes

Describe the methods by which all novel plant genotypes were produced. This includes those generated by transgenic approaches, gene editing, chemical/radiation-based mutagenesis and hybridization. For transgenic lines, describe the transformation method, the number of independent lines analyzed and the generation upon which experiments were performed. For gene-edited lines, describe the editor used, the endogenous sequence targeted for editing, the targeting guide RNA sequence (if applicable) and how the editor was applied.

Authentication

Describe any authentication procedures for each seed stock used or novel genotype generated. Describe any experiments used to assess the effect of a mutation and, where applicable, how potential secondary effects (e.g. second site T-DNA insertions, mosiacism, off-target gene editing) were examined.
