# Supplementary material for: Use of circulating tumour DNA to prospectively guide a switch from targeted to immune therapy in BRAF mutant advanced melanoma: the randomised phase II CAcTUS trial
Source: Nat Commun. 2026 May 21;17:6850. doi: 10.1038/s41467-026-72735-8 (PMC13389006; doi:10.1038/s41467-026-72735-8)
Supplement: Supplementary file 5 — Transparent Peer Review File [file 41467_2026_72735_MOESM5_ESM.pdf]

nature portfolio

Peer Review File

Use of circulating tumour DNA to prospectively guide a switch from targeted to immune therapy in BRAF mutant advanced melanoma: the randomised phase II CAcTUS trial

Corresponding Author: Dr Rebecca Lee

This manuscript has been previously reviewed at another journal. This document only contains information relating to versions considered at Nature Communications.

This file contains all reviewer reports in order by version, followed by all author rebuttals in order by version.

Version 0:

Reviewer comments:

Reviewer #3

(Remarks to the Author)
The authors have provided satisfactory responses to all the reviewers comments.
Nevertheless, my main concerns remain related to the study design and the small number of patients included. While the manuscript is clearly written and well presented, in my view, it may be more appropriate for a more specialized, technique-oriented journal focusing on molecular and liquid biopsy approaches.

Reviewer #4

(Remarks to the Author)
General Comments:

This is a very interesting study, examining the technical efficiency and utility of a ctDNA test. The rationale for the study is well justified in the background, and the methods and results support future examination of this guided treatment pathway/approach.

Minor Comments:

In lines 157-160, when making statements about higher/lower values between groups, a p-value should be provided to demonstrate whether the magnitude of the difference is likely chance or something systematic.

In the statistical analysis section, it is mentioned that the Wilcoxon Rank Sum test is used to compared between groups and time-points. Comparisons between time-points would be considered paired data and the Signed Rank test would be appropriate. This should be clarified.

Major Comments:

Any statements on the clinical interpretation of the results should be very lightly stated. The study was designed around technical endpoints, and is not set-up in terms of design nor sample size to make meaningful correlations with patient outcomes.

Version 1:

Reviewer comments:

Reviewer #5

(Remarks to the Author)
This manuscript reports a randomized trial with a clearly defined clinical question and transparent reporting of study procedures.

From a statistical perspective, however, the primary intent of this study is feasibility rather than efficacy. Framed as a pilot/feasibility study, the focus is appropriately on the practical implementation of a ctDNA-guided treatment strategy, and in this context a small sample size is acceptable. However, this also implies that efficacy-related questions were not a primary objective at the design stage, and the study is not adequate to support statements regarding treatment benefit or comparative effectiveness.

In particular, the heterogeneity of the control arm further reduces comparability between arms and effectively diminishes the usable sample size for any between-group interpretation. In addition, the manuscript presents a large number of exploratory analyses based on very small numbers of patients, in some cases only a few individuals. Some analyses rely on formal hypothesis testing despite very low power, while others are descriptive but are discussed in a manner that may imply more certainty than the data can support. Taken together, this substantially increases the risk of false-positive findings.

From my perspective, the current trial provides only very limited evidence regarding efficacy, and several statements in the Results and Discussion appear to overstate the strength of the observed signals. I would encourage the authors to more carefully balance signal generation against statistical uncertainty, and to explicitly frame these findings as exploratory and hypothesis-generating rather than indicative of clinical benefit.

Minor comments
1. The abstract would benefit from clearer presentation of the randomized trial structure, including the experimental and control arms being compared, as well as explicit reporting of the key feasibility outcomes with relevant numerical results and confidence intervals, rather than focusing predominantly on exploratory biological or efficacy-related findings.
2. For readability, the authors may consider using descriptive labels for the two trial arms (e.g. “control arm” and “experimental arm”) instead of Arm A and Arm B throughout the manuscript. In addition, for Arm B, while the protocol clearly specifies management once an ≥80% reduction in ctDNA is achieved, it is not clear how patients are managed if this threshold is not reached. Clarification of the decision pathway in this scenario would improve the logical completeness of the ctDNA-guided strategy.
3. The feasibility criteria described in the protocol (e.g. ≥95% return of critical results within 7 days and ≥80% of patients achieving an ≥80% reduction in BRAF VAF on targeted therapy) should be clearly described in the main text and explicitly linked to the corresponding results.
4. Where p-values are reported, the issue of multiple testing and false discovery should be addressed. In addition, the Discussion would benefit from more explicit caution regarding the interpretation of statistically significant findings derived from very small sample sizes.

Version 2:

Reviewer comments:

Reviewer #5

(Remarks to the Author)
Thank you for your detailed revisions and clarifications. The manuscript now more clearly frames the study as feasibility-focused and exploratory in nature, and the added statements regarding the interpretation of biomarker and immunotherapy-related analyses improve transparency.

I remain somewhat concerned that the immune-correlative and immunotherapy response–associated analyses are based on very small patient numbers and therefore carry a substantial degree of statistical uncertainty. However, as the exploratory nature and limitations of these findings are now explicitly acknowledged, I do not have further requests for revision.

Open Access This Peer Review File is licensed under a Creative Commons Attribution 4.0 International License, which permits use, sharing, adaptation, distribution and reproduction in any medium or format, as long as you give appropriate credit to the original author(s) and the source, provide a link to the Creative Commons license, and indicate if changes were made.
In cases where reviewers are anonymous, credit should be given to 'Anonymous Referee' and the source.
The images or other third party material in this Peer Review File are included in the article's Creative Commons license, unless indicated otherwise in a credit line to the material. If material is not included in the article's Creative Commons license and your intended use is not permitted by statutory regulation or exceeds the permitted use, you will need to obtain permission directly from the copyright holder.
To view a copy of this license, visit https://creativecommons.org/licenses/by/4.0/

Reviewer comments

Please find below our response to the reviewers

Reviewer #3 (Remarks to the Author):

Comment #1: The authors have provided satisfactory responses to all the reviewers comments. Nevertheless, my main concerns remain related to the study design and the small number of patients included. While the manuscript is clearly written and well presented, in my view, it may be more appropriate for a more specialized, technique-oriented journal focusing on molecular and liquid biopsy approaches.

Response: We are glad that the reviewer is satisfied that we have addressed all of the comments. We disagree that this manuscript should be limited to a more specialised audience. There is considerable interest across many tumour types as to how ctDNA can be best utilised in the clinic, with calls from clinicians for more prospective studies. We have specifically discussed how CAcTUS can be used to inform future clinical trial designs in melanoma and have provided data on aspects ranging from the assay optimisation (in supplemental) to defining inclusion criterion and relevance to RECIST. CAcTUS shows how ctDNA could be potentially used not just to inform when patients should be switched from one treatment to another in resistance, but also the concept that it could be used to define a switch at maximal treatment response. It will therefore add significantly to the discussion as to the different ways in which ctDNA can inform scheduling of treatments.

Reviewer #4 (Remarks to the Author):

General Comments:

Comment #2: This is a very interesting study, examining the technical efficiency and utility of a ctDNA test. The rationale for the study is well justified in the background, and the methods and results support future examination of this guided treatment pathway/approach.

Response: We thank the reviewer for their enthusiasm for our study and for highlighting that it supports further examination of ctDNA guided treatment in melanoma.

Minor Comments:

Comment #3: In lines 157-160, when making statements about higher/lower values between groups, a p-value should be provided to demonstrate whether the magnitude of the difference is likely chance or something systematic.

Response: We thank the reviewer for identifying this oversight and have added a p-value.

Comment #4: In the statistical analysis section, it is mentioned that the Wilcoxon Rank Sum test is used to compared between groups and time-points. Comparisons between time-points would be considered paired data and the Signed Rank test would be appropriate. This should be clarified.

Response: We have ensured that all comparisons between timepoints have been compared using the Wilcoxon signed rank test. We have added "or a Wilcoxon signed rank test when values could be paired." to the methods section.

Major Comments:

Comment #5: Any statements on the clinical interpretation of the results should be very lightly stated. The study was designed around technical endpoints, and is not set-up in terms of design nor sample size to make meaningful correlations with patient outcomes.

Response: Thank you for this comment - in response we have reviewed the discussion and changed can to may in line 314:

“This suggests that a short induction treatment with TT **may** result in rapid tumour control, modifying immunosuppressive features and potentially providing symptomatic benefit.”

In addition, we have altered the final sentence of the paragraph referring to clinical interpretation to “Thus, although CAcTUS was designed around technical endpoints, it highlights the utility of ctDNA to optimise scheduling treatment strategies before expanding to larger cohorts where correlations with patient outcomes can be tested.”

Reviewer comments

Please find below our response to the reviewers

Reviewer #5 (Remarks to the Author):

Comment #1: This manuscript reports a randomized trial with a clearly defined clinical question and transparent reporting of study procedures.

From a statistical perspective, however, the primary intent of this study is feasibility rather than efficacy. Framed as a pilot/feasibility study, the focus is appropriately on the practical implementation of a ctDNA-guided treatment strategy, and in this context a small sample size is acceptable. However, this also implies that efficacy-related questions were not a primary objective at the design stage, and the study is not adequate to support statements regarding treatment benefit or comparative effectiveness.

In particular, the heterogeneity of the control arm further reduces comparability between arms and effectively diminishes the usable sample size for any between-group interpretation. In addition, the manuscript presents a large number of exploratory analyses based on very small numbers of patients, in some cases only a few individuals. Some analyses rely on formal hypothesis testing despite very low power, while others are descriptive but are discussed in a manner that may imply more certainty than the data can support. Taken together, this substantially increases the risk of false-positive findings.

From my perspective, the current trial provides only very limited evidence regarding efficacy, and several statements in the Results and Discussion appear to overstate the strength of the observed signals. I would encourage the authors to more carefully balance signal generation against statistical uncertainty, and to explicitly frame these findings as exploratory and hypothesis-generating rather than indicative of clinical benefit.

Response: We thank the reviewer for their comments. We understand the reviewer's concerns about the heterogeneity of the control arm, however at the time of designing the study, both treatments were given as first line therapy depending on clinical features such as disease burden and location. Arm A as investigator choice was therefore felt to be more representative of standard of care and enabled better recruitment. The reviewer makes an important point about the exploratory nature of the study and therefore we have added further clarification into the discussion:

"Given the small sample size and exploratory nature of these analyses, statistically significant findings should be interpreted cautiously. These results are intended to inform hypothesis generation for future validation, rather than to support confirmatory inference."

Minor comments

Comment #2: The abstract would benefit from clearer presentation of the randomized trial structure, including the experimental and control arms being compared, as well as explicit reporting of the key feasibility outcomes with relevant numerical results and confidence intervals, rather than focusing predominantly on exploratory biological or efficacy-related findings.

Response: We thank the reviewer for their comment and have added "Patients were randomised to receive standard of care investigators choice of TT or CPI, switching to the alternative upon progression, or commencing TT and switching to CPI upon an ≥80% reduction of *BRAF VAF.* The study achieved the primary feasibility endpoints with 100% (95% CI: 94-100%) of critical results provided within 7 days to inform a clinical decision to switch and 100% of patients commencing TT achieving an ≥80% reduction of *BRAF* VAF (95% CI: 80-100%)*."*

Comment #3: For readability, the authors may consider using descriptive labels for the two trial arms (e.g. "control arm" and "experimental arm") instead of Arm A and Arm B throughout the manuscript. In addition, for Arm B, while the protocol clearly specifies management once an ≥80% reduction in ctDNA is achieved, it is not clear how patients are managed if this threshold is not reached. Clarification of the decision pathway in this scenario would improve the logical completeness of the ctDNA-guided strategy.

Response: We have removed Arm A+B throughout and replaced with control or experimental arm. We have added "If the ≥80% decrease in *BRAF* VAF was not achieved, patients would continue on TT."

Comment #4: The feasibility criteria described in the protocol (e.g. ≥95% return of critical results within 7 days and ≥80% of patients achieving an ≥80% reduction in BRAF VAF on targeted therapy) should be clearly described in the main text and explicitly linked to the corresponding results.

Response: We thank the reviewer for highlighting this.

We have added to the results: "A key primary endpoint for feasibility was therefore to assess whether ≥95% of samples could be processed and reported following quality assurance approval within 7 working days."

We have already linked the ctDNA reduction criteria to the results. "The primary endpoint of CAcTUS was to assess whether we would observe an ≥80% decrease in mutant *BRAF* VAF. All the patients who initially received TT across both arms (Arm A; n=6, Arm B; n=11), were observed to have an ≥80% reduction in % *BRAF* VAF from baseline, taking a median of 15 days (range 13-54 days) to reach the 80% reduction threshold (Fig. 1C)."

To the methods we have added "The primary feasibility endpoints of the study were i. ≥95% return of critical (red) ctDNA results returned within 7 working days of samples being received in the laboratory ii. ≥80% of patients with a decrease in ctDNA level of mutant BRAF≥80% on targeted therapy."

To the discussion we have added "meeting our feasibility criteria (≥95% return of critical results within 7 days)" and "We showed that all patients who initially received TT across both arms (n=17) had an ≥80% reduction of *BRAF* VAF, thus achieving the protocol-specified feasibility criteria."

Comment #5: Where p-values are reported, the issue of multiple testing and false discovery should be addressed. In addition, the Discussion would benefit from more explicit caution regarding the interpretation of statistically significant findings derived from very small sample sizes.

Response: We thank the reviewer for raising this important point. We agree that multiple testing can increase the risk of false positive findings, particularly when p-values are interpreted in a confirmatory framework. However, the analyses reporting p-values in this study are explicitly exploratory and

hypothesis-generating in nature, consistent with the feasibility-focused objectives of this small, randomised trial.

Given the limited sample size, formal multiplicity adjustment (e.g. Bonferroni or false discovery rate control) would be overly conservative and would substantially increase the risk of false negative findings, potentially obscuring biologically or clinically meaningful signals that are intended to inform prioritisation for future studies rather than to support definitive inference. Accordingly, p-values are presented descriptively and are not used as a basis for confirmatory claims.

In response to the reviewer's suggestion to address this more explicitly, we have clarified in the Methods and Discussion that p-values are unadjusted, that all biomarker and exploratory efficacy analyses should be interpreted with caution, and that any statistically significant findings should be considered preliminary and subject to validation in larger, adequately powered studies with prespecified multiplicity control.

Methods: "All biomarker and exploratory efficacy analyses were hypothesis-generating. Unadjusted p-values are reported descriptively alongside effect sizes and confidence intervals; no formal adjustment for multiple testing was applied."

Discussion: "Given the small sample size and exploratory nature of these analyses, statistically significant findings should be interpreted cautiously. These results are intended to inform hypothesis generation for future validation, rather than to support confirmatory inference."

Please find below our response to the reviewer

Reviewer #5
Thank you for your detailed revisions and clarifications. The manuscript now more clearly frames the study as feasibility-focused and exploratory in nature, and the added statements regarding the interpretation of biomarker and immunotherapy-related analyses improve transparency.

I remain somewhat concerned that the immune-correlative and immunotherapy response–associated analyses are based on very small patient numbers and therefore carry a substantial degree of statistical uncertainty. However, as the exploratory nature and limitations of these findings are now explicitly acknowledged, I do not have further requests for revision.

Response: Thank you for your time and comments that the transparency of the analysis has been improved. We have no further changes to make.
